# Monitoring childbirth and immediate postpartum care quality: A scoping review of indicators in high-income countries

**DOI:** 10.1371/journal.pone.0350665

**Published:** 2026-09-15

**Authors:** M. Allison Warren, Dayana Hamparsoumian, Elsa Lorthe, Gilles Cattani, Francisca Barcos Munoz, David Baud, Caroline Daelemans, Pauline De Vries, Manuella Epiney, Emanuela Gerhard, Markus Hodel, Marzia Loghi, Begoña Martinez De Tejada, Raegan MacSTravic, Beatrice Mosimann, Marianne Philibert, Tonia A. Rihs, Anne Steiner, Laurent Gaucher, Thomas Desplanches

**Affiliations:** 1 Geneva School of Health Sciences, HES-SO University of Applied Sciences and Arts Western Switzerland, Geneva, Switzerland; 2 Université Paris Cité and Université Sorbonne Paris Nord, Inserm, INRAE, Centre for Research in Epidemiology and Statistics, Paris, France; 3 Division of Neonatal and Pediatric Intensive Care of Women, Children and Adolescents, University Hospitals of Geneva, Geneva, Switzerland; 4 Obstetric Service, Woman-Mother-Child Department, Lausanne University Hospital (CHUV) and University of Lausanne (UNIL), Lausanne, Switzerland; 5 Faculty of Medicine, University of Geneva, Geneva Switzerland; 6 Department of the Woman, the Child and the Teenager, Geneva University Hospitals, Geneva, Switzerland; 7 Fédération Suisse des sages-femmes, Bern, Switzerland; 8 Department of Obstetrics and fetal-maternal Medicine Kanton Spital, Lucerne, Switzerland; 9 University of Lucerne, Health Department, Lucerne, Switzerland; 10 ISTAT, SWC Servizio Sistema integrato salute, assistenza e previdenza, DCSW Direzione Centrale per le Statistiche Sociali e il Welfare, DISD - Dipartimento per le Statistiche Sociali e Demografiche, Viale Liegi Roma, Italy; 11 Digital Workplace Squared, IT Skills and Solutions, Geneva, Switzerland; 12 Department of Obstetrics and Perinatal Medicine, Women’s Clinic, University Hospital Basel, Switzerland; 13 Federal Statistical Office, FSO, Neuchâtel, Switzerland; 14 Research on Healthcare Performance (RESHAPE), Université Claude Bernard Lyon 1, INSERM U1290, Lyon, France; University of Lausanne: Universite de Lausanne, SWITZERLAND

## Abstract

**Background:**

Most maternal and neonatal morbidity and mortality occur during childbirth and the immediate postnatal period. In high-income countries, where most births occur in maternity units, systematic monitoring of care quality is essential to improve safety and reduce preventable adverse outcomes.

**Objective:**

To identify maternal and neonatal indicators of quality-of-care at childbirth and immediate postnatal period in high-income countries, and to assess their level of validity.

**Methods:**

This scoping review followed PRISMA-ScR guidelines. A systematic search was conducted in April 2024 (updated March 2025) in Medline, Embase, CINAHL, and Web of Science. Eligible studies included literature from high-income countries reporting quality-of-care indicators. Indicators were extracted as reported and consolidated: those with aligned definitions were clustered into standardised indicators and subsequently grouped into broader constructs reflecting key dimensions of care. Indicators were grouped into four domains (access, structure, process, and outcome) and evaluated using a structured validity scoring system. Indicators were classified as high-validity if they included a clear definition (with specified numerator and denominator) and achieved a validity score of 3 or 4 on a 0–4 scale.

**Results:**

Eighty-four studies were included. In total, 981 individual indicators were identified; 546 were clearly defined and consolidated into 214 standardised indicators and 61 indicator constructs. Most focused on outcomes (n = 253, 46.3%) and processes (n = 243, 44.5%), with substantial heterogeneity in definitions. Fewer indicators addressed structure (n = 47, 8.6%) and access (n = 3, 0.6%). Nearly one third of all indicators demonstrated high-validity, mainly within outcome and process domains.

**Conclusions:**

This review identified a large set of quality-of-care indicators, reflecting sustained efforts to monitor care around childbirth. However, heterogeneity in definitions and variable validation limit comparability and routine use. These findings highlight the need for greater validation and standardisation of indicators, followed by the prioritisation of high-validity measures to support meaningful quality monitoring and improvement.

## Introduction

In high-income countries, nearly all births occur in maternity units and are attended by skilled health professionals, with overall good health outcomes [1]. Although maternal and neonatal mortality is rare [2–4], more than half of maternal deaths are considered preventable [5]. Maternal and neonatal morbidity, however, remain major concerns, particularly severe maternal morbidity related to postpartum haemorrhage and serious neonatal complications that continue to affect births even in high-income settings [6–9].

Interventions during childbirth play a crucial role in safeguarding maternal and neonatal health when appropriately indicated. Caesarean delivery is among the most frequently performed obstetric interventions worldwide [10,11]. However, its use illustrates a broader paradox in obstetric care: both underuse and overuse are associated with adverse outcomes. In line with World Health Organization (WHO) recommendations [12], very low caesarean rates are associated with increased maternal and neonatal mortality and morbidity [13,14], whereas excessively high rates are associated to higher maternal morbidity. Similar patterns have been observed for other common intrapartum interventions, with wide variations in use across high-income countries reflecting unwarranted differences in practice [15]. These findings highlight the need for regular monitoring of obstetric care.

To address these challenges, the concept of quality of care provides a comprehensive framework for evaluating and improving maternal and newborn health services. Perinatal quality of care refers to the degree to which health services during pregnancy, childbirth, and the postnatal period maximize the likelihood of optimal health outcomes and adhere to professional standards and evidence-based practice [16,17]. It encompasses five domains; access, structure, process, patient experience, and outcomes [18], in line with the Donabedian model [19].

Monitoring perinatal indicators is essential for developing effective strategies to improve quality of care. Indicators can be defined as “standardised, evidence-based measures of health care quality that can be used with readily available hospital inpatient administrative data to measure and track clinical performance and outcome“ [17]. In high-income settings, the widespread availability of large medico-administrative databases provides an opportunity to systematically monitor such indicators.

While quality indicators are relevant throughout pregnancy and the postpartum period [20], the time of childbirth is particularly critical for assessing care quality, as it concentrates the highest risks for both mother and newborn [6,21,22]. This has led to the development of numerous indicators intended to monitor care during labour and the immediate postnatal period.

However, beyond data availability, indicators must be validated before risk-adjusted standardisation and benchmarking. This requirement is particularly important for cross-country comparisons, which are valuable for rare but serious outcomes such as maternal and neonatal mortality, yet remain constrained by variations in data completeness and indicator validation across health systems [23]. At the local level, maternity units require specific indicators that more accurately reflect the quality of routine care. These should capture events that are sufficiently frequent to allow regular analysis, be reproducible across institutions, and be sensitive to changes in practice over time, making continuous quality monitoring both feasible and sustainable.

This study aimed to identify indicators developed to assess the quality of maternal and neonatal care during childbirth and the immediate postnatal period in high-income countries, and to assess their level of validity.

## Materials and methods

### Design

This scoping review was conducted in accordance with the Joanna Briggs Institute (JBI) methodology and is reported following the Preferred Reporting Items for Systematic reviews and Meta-Analyses extension for Scoping Reviews (PRISMA-ScR) [24] (S1 File).

### Conceptual framework

The study was guided by the Society for Maternal–Fetal Medicine (SMFM) [18] framework. Indicators were organised into four domains: access, structure, process, and outcome, to provide a clear taxonomy for classification and to support systematic analysis of indicators.

### Eligibility criteria and types of studies

We followed the JBI methodology and applied the Population–Concept–Context (PCC) framework to define eligibility criteria and guide the search strategy. The review focused on: (1) Population, covering maternal and neonatal care during labour, birth, and the early postnatal period; (2) Concept, defined as quality of care indicators; and (3) Context, limited to high-income countries as classified by the World Bank [25].

Studies reporting at least one indicator assessing the quality of maternal and/or neonatal care during labour, birth, or within the first seven days postpartum were eligible for inclusion. No restrictions were applied on sample size, year of publication, or data source, but only English-language full texts were considered. Studies were excluded if they focused on populations, outcomes, or time periods outside the scope of this review, or if the indicator related exclusively to phases of care other than childbirth and the immediate postpartum period. Publications were also excluded if the study design was not suitable for data extraction (commentaries, protocols, reviews, conference abstracts, and case reports or case series).

### Search strategy and screening processes

To identify all relevant studies, a systematic search was conducted on 19 April 2024, using the following electronic bibliographic databases: Medline (via PubMed), Cumulative Index to Nursing and Allied Health Literature (CINAHL), Embase, and Web of Science. The search strategy was developed in consultation with subject experts and the academic liaison librarian of the University of Applied Sciences and Arts Western Switzerland in Geneva, refined through multiple discussions, and then executed across the selected databases. The electronic search strategy was developed in accordance with the Peer Review of Electronic Search Strategies (PRESS) 2015 Guideline Statement. Controlled vocabulary (e.g., Medical Subject Headings) and free-text keywords, including truncations, were combined to capture each concept comprehensively (S2 File). Searches were conducted in titles, abstracts, and keywords using Boolean and proximity operators as appropriate. On 24 March 2025, an update was conducted to check for additional potential articles.

**Fig 1:**
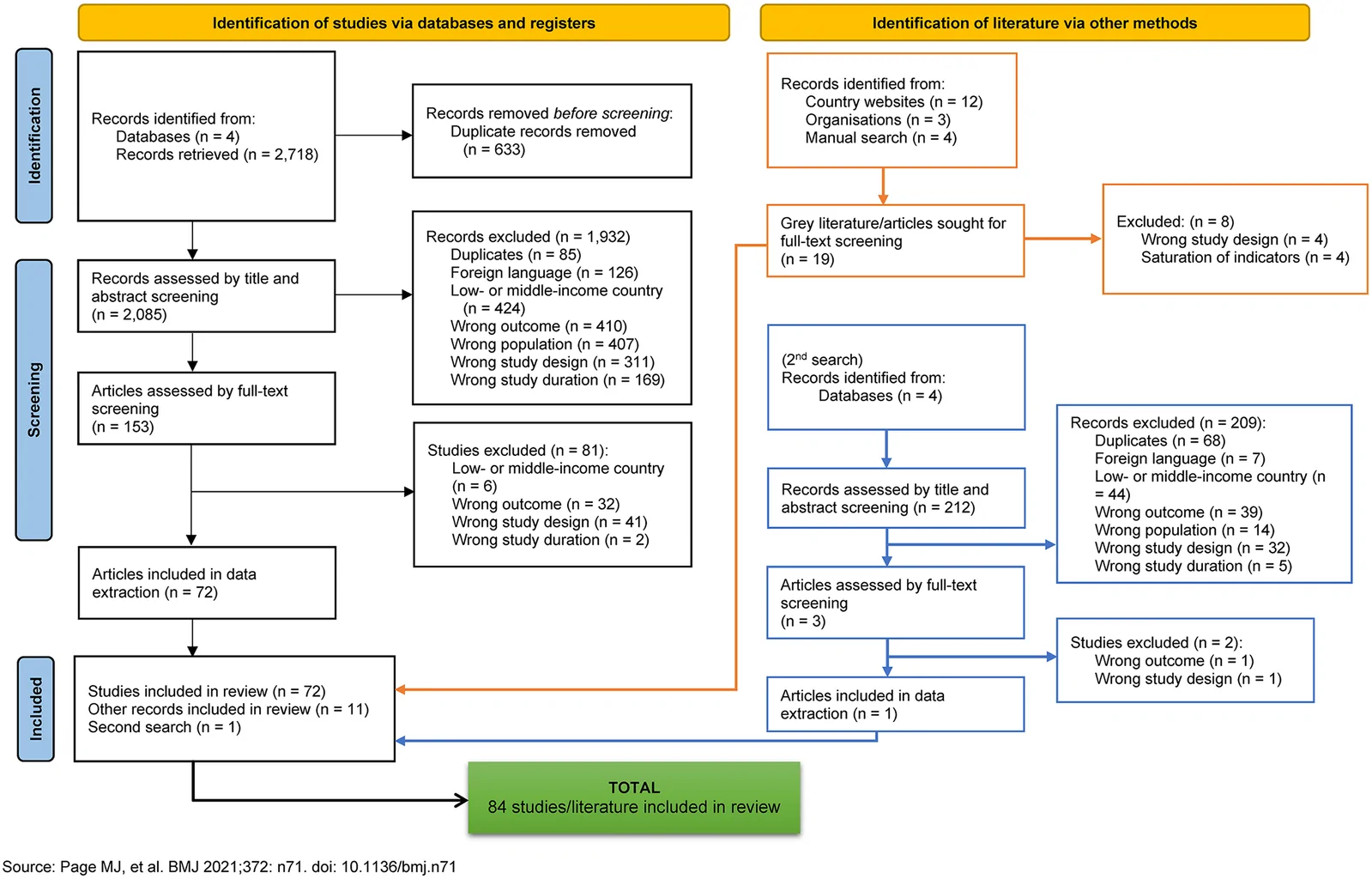
PRISMA 2020 flow diagram of the study selection process. Flow diagram illustrating the identification, screening, eligibility assessment, and inclusion of studies included in this scoping review.

All references were imported into EndNote library version X9 for de-duplication and exported to the Rayyan platform. Two researchers independently screened titles and abstracts against the eligibility criteria. Full texts of relevant studies were then retrieved and reviewed for eligibility. Discrepancies at any stage were resolved through discussion, with adjudication by a third reviewer when necessary. Studies were excluded according to the predefined eligibility criteria described above. Reasons for exclusion at the full-text screening stage are reported in the PRISMA flow diagram (Fig 1). Reference lists of included articles were also screened to identify additional relevant studies (Fig 1). Manual searches of national and international indicator databases from high-income countries were initially conducted in April 2024 and updated in March 2025, based on the World Bank classification [25]. Searches were performed using the Google search engine with the search term “maternal and neonatal quality-of-care health indicators”, with a particular focus on national organizations responsible for developing or reporting maternal and neonatal quality-of-care indicators. Article characteristics were summarised by country, data source (e.g., medico-administrative, medical records), and reported methodology (e.g., validation study, consensus panel).

### Data extraction, classification and validity of indicators

Indicators were systematically extracted from each resource, compiling all available data, including indicator name, definition, numerator, and denominator. Indicators based on patient-reported experience and outcome measures (PREM or PROM) were not extracted because they were outside the scope of this review. All extracted indicators were verified for accuracy and completeness. Only complete indicators (defined as those with a name, definition, numerator, and denominator) were included in the analysis.

To enable systematic analysis of underlying concepts, a stepwise funnel approach was used to organize the indicators. First, all individual indicators were extracted and reviewed as originally reported. Individual indicators with closely aligned definitions and overlapping terminology were then clustered into standardised indicator labels (hereafter referred to as standardised indicators), representing sets of indicators that measured essentially the same concept using comparable definitions. These standardised indicators constituted the primary unit of analysis.

Next, related standardised indicators were aggregated into indicator constructs, which were subsequently organized within broader conceptual categories reflecting major dimensions of care (e.g., indicators of third- or fourth-degree tears grouped under the construct *Perineal tears*).

To provide a structured assessment of the level of validity of the identified quality indicators, we adapted the validity scale from a previous systematic review [8]. Expert consensus methods (e.g., Delphi panels), which are widely used in quality indicator development, were not adequately represented in the original scoring system. We therefore expanded the scale to better reflect these approaches. Thus, the revised scoring system was as follows: 0 = no validation information; 1 = selected by authors with described methods; 2 = based on expert consensus with methods; 3 = used in prior research (e.g., a pilot study validating indicators from an expert panel); 4 = validated by national/international authority. This approach allowed reproducible assessment and comparison of indicator validity across studies. Complete indicators with validity scores ≥3 were classified as high-validity indicators and considered to have stronger measurement properties, allowing comparison of validity patterns across domains and publication periods.

## Results

### Search and screening outcomes

The initial search yielded 2,718 articles. After removing duplicates, 2,085 articles were screened in Rayyan, resulting in 153 articles retained at the title-abstract stage. Following full-text screening, 72 articles were included. Four additional articles were identified from bibliographies and 15 from national or international indicator databases, of which 11 were included. The updated search additionally identified 212 articles; 3 underwent full-text screening, with 1 included. In total, 84 articles were included for data extraction (Fig 1). These studies are cited in References [9,17,20,26–106].

### Characteristics of studies

The 84 included articles span 1989–2025, with 5 published before 2000 and 19 since 2020 (S1 Table). Studies covered 14 countries plus two multinational sources. Nearly one-third originated from the Unites States of America (USA), while European studies contributed the largest share with 36 articles providing 443 indicators – almost half of all extracted indicators, with the Netherlands (17%) and France (11%) providing a significant amount of the overall indicators. Most articles (52%, n = 44) used medico-administrative databases, 18% (n = 15) relied on data extracted from medical records, and 30% (n = 25) reported no patient data. Validation studies represented 21% (n = 18), while consensus studies accounted for 27% (n = 23), and 51% (n = 43) were observational studies (S1 Table).

### Overview of extracted indicators

From the 84 included articles, a total of 981 individual indicators were extracted and recorded as originally reported. After exclusion of socio-demographic (n = 26, 2.7%) (S2 Table) and incomplete indicators (n = 409, 41.7%) (S3 Table), 546 (55.7%) complete indicators were retained for analysis, of which 134 were reported only once across the included sources. Using the stepwise funnel approach, these indicators were organised into 61 indicator constructs and 214 standardised indicators.

By domain, indicators were classified as outcome (n = 253, 46.3%), process (n = 243, 44.5%), structure (n = 47, 8.6%), or access (n = 3, 0.6%) (See the complete list in S3 File: Perinatal Care Quality Indicators).

The 253 outcome indicators were grouped into 36 indicators constructs, and 94 standardised indicators (S4, S5, S6 Tables). Mortality was a prominent theme, comprising 46 indicators (18.2%) across maternal, neonatal, and foetal populations. In addition, maternal and neonatal morbidity outcome indicators were identified, including severe maternal morbidity (n = 19), obstetric trauma (n = 13), infection (n = 10), prematurity (n = 9), neonatal asphyxia (n = 4), and ICU (n = 7)/ NICU admission (n = 9). Indicators relating to perineal tears deserve special mention, as 42 indicators were extracted on this construct alone. Three composite indicators integrating maternal and neonatal outcomes were also reported, including the Adverse Outcome Index and related severity scores (S6 Table). Overall, significant variability in definitions and operationalisation was observed across outcome concepts.

The 243 process indicators were aligned under 20 indicator constructs, containing 82 standardised indicators (S7, S8, S9 Tables). Maternal process indicators mainly reflected intrapartum care, including labour onset (n = 13) and management (n = 19), pain relief (n = 5), and mode of delivery (n = 98), with caesarean section (n = 65) emerging as one of the most extensively developed process indicators. A substantial proportion also focused on postpartum management, particularly prevention (n = 11) and management (n = 27) of postpartum haemorrhage, blood transfusion (n = 7), and post-caesarean care (n = 7). Additional maternal process indicators addressed prematurity-related care (n = 6), and maternal screening practices (n = 4) (S7 Table).

Neonatal process indicators (n = 12) were all unique and mainly addressed immediate postnatal care, including delivery room resuscitation, respiratory support, and essential preventive treatments. Additional indicators covered evidence-based therapies for preterm or high-risk infants and neonatal screening practices (S8 Table).

Quality of care indicators relating to both mothers and their neonates focused on infant feeding (n = 14) and skin-to-skin contact (n = 2) (S9 Table).

The 47 structure and 3 access indicators were organised into 6 indicator constructs (Organisation, Evaluation, Resources, Communication, Labour management, and Counselling on mode of delivery (S10 Table)) and 39 standardised indicators, of which 32 were unique.

### Validity of extracted indicators

Overall, 278 indicators met the criteria for high-validity, defined as complete indicators with a validity score ≥3, representing 29.1% of all identified indicators (278/955). Among these, 132 indicators (13.8%) had a validity score of 3 and 146 (15.3%) had a score of 4.

Among complete indicators, the proportion of high-quality indicator was 50.9%. High-validity indicators were predominantly observed in the outcome and process domains. In contrast, only a small number of structure indicators and no access indicators reached the high-validity threshold (Table 1).

**Table 1:** Distribution of validity scores by domain in complete indicators.

Domain	Total number of indicators	Validity score, n (%)				
		4	3	2	1	0
**Outcome**	253	95 (17.4)	68 (12.5)	72 (13.2)	16 (2.9)	2 (0.4)
**Process**	243	47 (8.6)	58 (10.6)	119 (21.8)	17 (3.1)	2 (0.4)
**Structure**	47	4 (0.7)	6 (1.1)	35 (6.4)	2 (0.4)	0
**Access**	3	0	0	3 (0.6)	0	0
**Total**	546	146 (26.7)	132 (24.2)	229 (41.9)	35 (6.4)	4 (0.7)

## Discussion

In this scoping review, we identified a large and heterogeneous set of quality of care indicators developed to assess maternal and neonatal care during childbirth and the immediate postnatal period in high-income countries. Using a stepwise funnel approach, 546 complete individual indicators were grouped into 214 standardised indicators with closely aligned definitions and aggregated into 61 indicator constructs representing key dimensions of perinatal quality of care. Most indicators focused on health outcomes and care processes, whereas structure and access indicators were less frequently represented. Nearly one third of all indicators demonstrated a high level of validity, mainly within outcome and process domains. These findings reflect sustained efforts to monitor care during childbirth and early postpartum but also reveal substantial variation in indicator definitions, levels of validation and scope.

Our findings extend previous work by providing an updated synthesis specifically focused on the intrapartum and immediate postnatal periods. In 2018, a scoping review compiled current indicators from selected global multi-stakeholder initiatives to support harmonized monitoring of maternal and newborn health and identified 140 indicators [108]. A subsequent systematic review published in 2019 examined 87 indicators across the maternal and child (up to age 18) continuum of care using scientific literature, indicator sets, and grey literature, but was limited to publications from 2012 to 2016 [109]. By consolidating the evidence from high-income countries without time constraints, our review provides an updated and detailed overview of quality of care indicators at the time of birth.

In addition to the large number of available indicators, our results underscore the presence of major methodological challenges. Multiple operational definitions frequently coexist for the same underlying concept, affecting core measurement elements. This heterogeneity limits comparability across settings and limits the formation of broad agreement among stakeholders. Although consensus-based approaches have been proposed to address these issues [68,76,110], the growing number of indicators may hinder the feasibility of standardisation. Also, the breadth of high-validity indicators identified in this review highlights the need to move from compilation towards prioritisation. Establishing a core set of indicators requires explicit consideration of the level at which they are intended to be used – whether for international benchmarking, national monitoring, or centre-level quality improvement. It also requires assessing their feasibility, particularly when they are derived from existing or routinely collected data. These considerations are central to ensuring that consensus-driven indicator selection is both methodologically robust and operationally viable.

Regional differences in healthcare systems, policy priorities, and data infrastructures may also influence the development, selection, and implementation of quality indicators. Future research should explore these contextual differences to better understand their impact on the comparability and applicability of quality indicators across high-income countries.

Our findings also highlight the complexity of assessing perinatal quality of care. In many contexts, meaningful assessment requires the combined interpretation of complementary indicators, particularly when practices involve trade-offs between benefits and risks. For example, interpreting episiotomy policies alongside rates of severe perineal tears provides a more nuanced understanding of obstetric practice than either indicator alone [111]. Similarly, our indicator classification underscores the multidimensional nature of care quality for a given clinical topic. Postpartum haemorrhage illustrates this complexity, with indicators spanning care processes (e.g., prevention, obstetric and anaesthesiologic management), structural indicators (e.g., resource availability), and health outcomes (e.g., mortality, severe complications). Together, these dimensions reflect different but complementary facets of care quality that are rarely captured by a single measure. Furthermore, quality assessment at birth is further complicated by the dual consideration of maternal and neonatal outcomes, as interventions beneficial for one may not confer equivalent benefits for the other [107,112]. Future research should emphasise the joint collection and interpretation of complementary indicators within a given clinical theme to reflect the multidimensional nature of routine maternity care.

Indicator quality itself remains a major concern. A substantial proportion (42%) of indicators identified in this review were incompletely reported, lacking the basic elements required for meaningful measurement, including clear definitions and specified numerators or denominators. As a result, these indicators cannot be consistently interpreted or implemented across settings, highlighting important limitations in the current quality indicator literature. Among the indicators identified, assessment of validity highlighted additional limitations, with approximately one third demonstrating high validity. Consistent with previous work [113], these findings highlight the need for clearer validation standards and appropriate validation methods, as well as a shared terminology to support meaningful measurement and comparison of perinatal quality of care.

Finally, this synthesis highlights gaps in the current indicator landscape, as indicators regarding access and structure remain relatively underdeveloped and insufficiently validated. Although these indicators may capture important system-level dimensions of care, their measurement properties and relevance for routine monitoring require further investigation.

### Strengths and limitations

This scoping review applied a rigorous methodology to enhance the reliability, transparency, and reproducibility of its findings in accordance with PRISMA-ScR guidelines. To support a comprehensive synthesis, extracted indicators were subjected to a multi-dimensional analysis, including thematic grouping, and classification by both population and domain (access, structure, process, and outcome). This approach enabled a structured overview of a large and heterogeneous body of indicators.

Several limitations should nonetheless be acknowledged. Although no protocol was prospectively registered or published for this scoping review, all methodological steps, including the eligibility criteria, search strategy, study selection process, data extraction, and analytical approach, are reported in sufficient detail to support the transparency and reproducibility of the review. Despite a comprehensive search strategy covering four major electronic databases and grey literature sources, it remains possible that some relevant publications were not identified. However, the breadth of sources included and the convergence of indicators across studies suggest that most commonly used quality of care indicators were likely captured. No formal critical appraisal of the included studies was undertaken. Although this is consistent with current methodological guidance for scoping reviews when such an appraisal is not required to address the review objectives [114], it limits the ability to comment on the methodological quality of the included evidence. In addition, as this review also assessed the level of validity of the identified quality indicators, we applied an adapted validity scale based on prior work. This assessment focused on the reported methods used for the development and validation of the identified quality indicators and was therefore dependent on the completeness of reporting in the included publications. More broadly, this limitation reflects a well-recognised challenge in the field. As previously highlighted by Saturno-Hernández et al. [109], fewer than 10% of quality indicators met established criteria for scientific soundness, underscoring the need for more rigorous development, validation, and reporting of quality indicators. Methodologically, we chose to exclude PROMs and PREMs, despite their recognised importance for capturing care quality from the service user’s perspective. Future research should specifically focus on integrating these dimensions into perinatal quality measurement frameworks.

## Conclusion

This scoping review has identified a wide range of indicators developed to assess the quality of maternal and neonatal care during childbirth and the immediate postnatal period in high-income countries. Although many indicators focus on clinical outcomes and care processes, their comparability and routine use is limited by substantial heterogeneity in definitions and variable levels of validation. By organising existing indicators into a structured dataset, this review provides a foundation for more consistent and transparent assessment of care quality. Progress in this field will require greater standardisation of indicator definitions, more robust validation, and the prioritisation of high-validity measures. Their context-sensitive implementation in routine maternity care is essential to ensure meaningful and sustainable monitoring and to support the development of a coherent set of core indicators.

## Supporting information

S1 File Preferred Reporting Items for Systematic reviews and Meta-Analyses extension for Scoping Reviews (PRISMA-ScR) Checklist. JBI = Joanna Briggs Institute; PRISMA-ScR = Preferred Reporting Items for Systematic reviews and Meta-Analyses extension for Scoping Reviews. * Where *sources of evidence* (see second footnote) are compiled from, such as bibliographic databases, social media 7platforms, and Web sites. † A more inclusive/heterogeneous term used to account for the different types of evidence or data sources (e.g., quantitative and/or qualitative research, expert opinion, and policy documents) that may be eligible in a scoping review as opposed to only studies. This is not to be confused with *information sources* (see first footnote). ‡ The frameworks by Arksey and O’Malley [6] and Levac and colleagues [7] and the JBI guidance [4,5] refer to the process of data extraction in a scoping review as data charting. § The process of systematically examining research evidence to assess its validity, results, and relevance before using it to inform a decision. This term is used for items 12 and 19 instead of “risk of bias” (which is more applicable to systematic reviews of interventions) to include and acknowledge the various sources of evidence that may be used in a scoping review (e.g., quantitative and/or qualitative research, expert opinion, and policy document).
(DOCX)

S2 File Research strategy. (DOCX)

S3 File Final included articles Perinatal Care Quality Indicators. (HTML)

S1 Table Characteristics of included studies. (DOCX)

S2 Table Socio-demographic indicators. (DOCX)

S3 Table List of incomplete indicators. (DOCX)

S4 Table List of maternal outcome indicators (N = 132). (DOCX)

S5 Table List of foetal and neonatal outcome indicators (N = 118). (DOCX)

S6 Table List of maternal and neonatal outcome indicators (N=3). (DOCX)

S7 Table List of complete maternal process indicators (N = 210). (DOCX)

S8 Table List of complete neonatal process indicators (N = 12). (DOCX)

S9 Table List of complete maternal and neonatal process indicators (N = 21). (DOCX)

S10 Table List of complete structure and access indicators (N = 50). (DOCX)
