# Supplementary material for: Monitoring childbirth and immediate postpartum care quality: A scoping review of indicators in high-income countries
Source: PLoS One. 2026 Sep 15;21(9):e0350665. doi: 10.1371/journal.pone.0350665 (PMC13577461; doi:10.1371/journal.pone.0350665)
Supplement: Supplementary file 2 [file pone.0350665.s002.docx]

**S2 File. Research strategy**

*First search*

Search undertaken on 19.04.2024

**MEDLINE via PubMed**

(("Parturition"[Mesh] OR "Delivery, Obstetric"[Mesh] OR "Labor, Obstetric"[Mesh] OR "Peripartum Period"[Mesh] OR "Perinatal Care"[Mesh] OR "Postpartum Period"[Mesh] OR "Perinatology"[Mesh] OR "Pregnancy"[Mesh] OR pregnan*[Title/Abstract] OR obstetri*[Title/Abstract] OR prenatal*[Title/Abstract] OR peri-partum[Title/Abstract] OR peripartum[Title/Abstract] OR parturi*[Title/Abstract] OR birth*[Title/Abstract] OR childbirth*[Title/Abstract] OR postpartum*[Title/Abstract] OR post-partum*[Title/Abstract] OR postnatal*[Title/Abstract] OR post-natal*[Title/Abstract] OR puerper*[Title/Abstract] OR perinatal*[Title/Abstract] OR perinatol*[Title/Abstract] OR maternal-fetal medicine[Title/Abstract]) (("Quality Indicators, Health Care"[Mesh] OR clinical indicator*[Title/Abstract]) OR ((healthcare[Title/Abstract] OR health[Title/Abstract] OR care[Title/Abstract] OR core[Title/Abstract]) AND (quality indicator*[Title/Abstract])))) **AND** ("Quality Assurance, Health Care"[Mesh] OR "Data Collection"[Mesh] OR "Research Report"[Mesh] OR monitoring[Title/Abstract] OR benchmarking[Title/Abstract] OR audit*[Title/Abstract] OR measurement*[Title/Abstract] OR health surveillance[Title/Abstract] OR report*[Title/Abstract] OR survey*[Title/Abstract] OR regist*[Title/ **AND** Abstract] OR statistic*[Title/Abstract] OR system*[Title/Abstract] OR record*[Title/Abstract] OR hospital discharge[Title/Abstract])

**CINAHL Complete**

( ( (MH "Delivery, Obstetric+") OR (MH "Labor+") OR (MH "Perinatal Period") OR (MH "Perinatal Care") OR (MH "Postnatal Period+") OR (MH "Perinatology") OR (MH "Pregnancy+") ) OR TI ( pregnan* OR obstetri* OR prenatal* OR peri-partum OR peripartum OR parturi* OR birth* OR childbirth* OR postpartum* OR post-partum* OR postnatal* OR post-natal* OR puerper* OR perinatal* OR perinatol* OR maternal-fetal medicine ) OR AB ( pregnan* OR obstetri* OR prenatal* OR peri-partum OR peripartum OR parturi* OR birth* OR childbirth* OR postpartum* OR post-partum* OR postnatal* OR post-natal* OR puerper* OR perinatal* OR perinatol* OR maternal-fetal medicine ) ) **AND** ( ( MH "Clinical Indicators" OR TI "clinical indicator*" OR AB "clinical indicator*" ) OR ( ( TI ( healthcare OR health OR core OR care ) OR AB ( healthcare OR health OR core OR care ) ) AND ( TI "quality indicator*" OR AB "quality indicator*" ) ) ) **AND** ( (MH "Data Collection") OR TI ( monitoring OR benchmarking OR audit* OR measurement* OR "health surveillance" OR report* OR survey* OR regist* OR statistic* OR system* OR record* OR "hospital discharge" ) OR AB ( monitoring OR benchmarking OR audit* OR measurement* OR "health surveillance" OR report* OR survey* OR regist* OR statistic* OR system* OR record* OR "hospital discharge" ) )

**Embase**

('birth'/exp OR 'obstetric delivery'/exp OR 'labor'/exp OR 'perinatal period'/exp OR 'perinatal care'/exp OR 'puerperium'/exp OR 'perinatology'/exp OR 'pregnancy'/exp OR pregnan*:ti,ab,kw OR obstetri*:ti,ab,kw OR prenatal*:ti,ab,kw OR 'peri partum':ti,ab,kw OR peripartum:ti,ab,kw OR parturi*:ti,ab,kw OR birth*:ti,ab,kw OR childbirth*:ti,ab,kw OR postpartum*:ti,ab,kw OR 'post partum*':ti,ab,kw OR postnatal*:ti,ab,kw OR 'post natal*':ti,ab,kw OR puerper*:ti,ab,kw OR perinatal*:ti,ab,kw OR perinatol*:ti,ab,kw OR 'maternal-fetal medicine':ti,ab,kw) AND ((healthcare:ti,ab,kw OR health:ti,ab,kw OR core:ti,ab,kw OR care:ti,ab,kw) **AND** 'quality indicator*':ti,ab,kw OR 'clinical indicator'/exp OR 'clinical indicator') **AND** ('information processing'/exp OR monitoring:ti,ab,kw OR benchmarking:ti,ab,kw OR audit*:ti,ab,kw OR measurement*:ti,ab,kw OR 'health surveillance':ti,ab,kw OR report*:ti,ab,kw OR survey*:ti,ab,kw OR regist*:ti,ab,kw OR statistic*:ti,ab,kw OR system*:ti,ab,kw OR record*:ti,ab,kw OR 'hospital discharge':ti,ab,kw)

**Web of science**

(TS=(pregnan* OR obstetri* OR prenatal* OR peri-partum OR peripartum OR parturi* OR birth* OR childbirth* OR postpartum* OR post-partum* OR postnatal* OR post-natal* OR puerper* OR perinatal* OR perinatol* OR maternal-fetal medicine)) AND (TS=("clinical indicator*") OR (TS=("quality indicator*") **AND** TS=(healthcare OR health OR core OR care ))) **AND** (TS=(monitoring OR benchmarking OR audit* OR measurement* OR "health surveillance" OR report* OR survey* OR regist* OR statistic* OR system* OR record* OR "hospital discharge"))

*Second search*

**Medline via PubMed**

**From 2024/4/15 to 2025/3/31**

(("Parturition"[Mesh] OR "Delivery, Obstetric"[Mesh] OR "Labor, Obstetric"[Mesh] OR "Peripartum Period"[Mesh] OR "Perinatal Care"[Mesh] OR "Postpartum Period"[Mesh] OR "Perinatology"[Mesh] OR "Pregnancy"[Mesh] OR pregnan*[Title/Abstract] OR obstetri*[Title/Abstract] OR prenatal*[Title/Abstract] OR peri-partum[Title/Abstract] OR peripartum[Title/Abstract] OR parturi*[Title/Abstract] OR birth*[Title/Abstract] OR childbirth*[Title/Abstract] OR postpartum*[Title/Abstract] OR post-partum*[Title/Abstract] OR postnatal*[Title/Abstract] OR post-natal*[Title/Abstract] OR puerper*[Title/Abstract] OR perinatal*[Title/Abstract] OR perinatol*[Title/Abstract] OR maternal-fetal medicine[Title/Abstract]) **AND** (("Quality Indicators, Health Care"[Mesh] OR clinical indicator*[Title/Abstract]) OR ((healthcare[Title/Abstract] OR health[Title/Abstract] OR care[Title/Abstract] OR core[Title/Abstract]) AND (quality indicator*[Title/Abstract])))) **AND** ("Quality Assurance, Health Care"[Mesh] OR "Data Collection"[Mesh] OR "Research Report"[Mesh] OR monitoring[Title/Abstract] OR benchmarking[Title/Abstract] OR audit*[Title/Abstract] OR measurement*[Title/Abstract] OR health surveillance[Title/Abstract] OR report*[Title/Abstract] OR survey*[Title/Abstract] OR regist*[Title/Abstract] OR statistic*[Title/Abstract] OR system*[Title/Abstract] OR record*[Title/Abstract] OR hospital discharge[Title/Abstract]) AND (2024/4/15:2025/3/31[pdat])

**CINAHL Complete**

**Publication Date: 20240415-20250331**

( ( ( (MH "Delivery, Obstetric+") OR (MH "Labor+") OR (MH "Perinatal Period") OR (MH "Perinatal Care") OR (MH "Postnatal Period+") OR (MH "Perinatology") OR (MH "Pregnancy+") ) OR TI ( pregnan* OR obstetri* OR prenatal* OR peri-partum OR peripartum OR parturi* OR birth* OR childbirth* OR postpartum* OR post-partum* OR postnatal* OR post-natal* OR puerper* OR perinatal* OR perinatol* OR maternal-fetal medicine ) OR AB ( pregnan* OR obstetri* OR prenatal* OR peri-partum OR peripartum OR parturi* OR birth* OR childbirth* OR postpartum* OR post-partum* OR postnatal* OR post-natal* OR puerper* OR perinatal* OR perinatol* OR maternal-fetal medicine ) ) AND ( ( MH "Clinical Indicators" OR TI "clinical indicator*" OR AB "clinical indicator*" ) OR ( ( TI ( healthcare OR health OR core OR care ) OR AB ( healthcare OR health OR core OR care ) ) **AND** ( TI "quality indicator*" OR AB "quality indicator*" ) ) ) **AND** ( (MH "Data Collection") OR TI ( monitoring OR benchmarking OR audit* OR measurement* OR "health surveillance" OR report* OR survey* OR regist* OR statistic* OR system* OR record* OR "hospital discharge" ) OR AB ( monitoring OR benchmarking OR audit* OR measurement* OR "health surveillance" OR report* OR survey* OR regist* OR statistic* OR system* OR record* OR "hospital discharge" ) ) ) AND DT 20240415-20250331

**Embase**

**Publication years: 2024-2025**

('birth'/exp OR 'obstetric delivery'/exp OR 'labor'/exp OR 'perinatal period'/exp OR 'perinatal care'/exp OR 'puerperium'/exp OR 'perinatology'/exp OR 'pregnancy'/exp OR pregnan*:ti,ab,kw OR obstetri*:ti,ab,kw OR prenatal*:ti,ab,kw OR 'peri partum':ti,ab,kw OR peripartum:ti,ab,kw OR parturi*:ti,ab,kw OR birth*:ti,ab,kw OR childbirth*:ti,ab,kw OR postpartum*:ti,ab,kw OR 'post partum*':ti,ab,kw OR postnatal*:ti,ab,kw OR 'post natal*':ti,ab,kw OR puerper*:ti,ab,kw OR perinatal*:ti,ab,kw OR perinatol*:ti,ab,kw OR 'maternal-fetal medicine':ti,ab,kw) **AND** ((healthcare:ti,ab,kw OR health:ti,ab,kw OR core:ti,ab,kw OR care:ti,ab,kw) **AND** 'quality indicator*':ti,ab,kw OR 'clinical indicator'/exp OR 'clinical indicator') AND ('information processing'/exp OR monitoring:ti,ab,kw OR benchmarking:ti,ab,kw OR audit*:ti,ab,kw OR measurement*:ti,ab,kw OR 'health surveillance':ti,ab,kw OR report*:ti,ab,kw OR survey*:ti,ab,kw OR regist*:ti,ab,kw OR statistic*:ti,ab,kw OR system*:ti,ab,kw OR record*:ti,ab,kw OR 'hospital discharge':ti,ab,kw) AND (2024:py OR 2025:py)

**Web of science**

**2024-04-15 to 2025-03-31 (publication date)**

(TS=(pregnan* OR obstetri* OR prenatal* OR peri-partum OR peripartum OR parturi* OR birth* OR childbirth* OR postpartum* OR post-partum* OR postnatal* OR post-natal* OR puerper* OR perinatal* OR perinatol* OR maternal-fetal medicine)) **AND** (TS=("clinical indicator*") OR (TS=("quality indicator*") AND TS=(healthcare OR health OR core OR care ))) **AND** (TS=(monitoring OR benchmarking OR audit* OR measurement* OR "health surveillance" OR report* OR survey* OR regist* OR statistic* OR system* OR record* OR "hospital discharge")) AND DOP=(2024-04-15/2025-03-31)
