# Supplementary material for: Monitoring childbirth and immediate postpartum care quality: A scoping review of indicators in high-income countries
Source: PLoS One. 2026 Sep 15;21(9):e0350665. doi: 10.1371/journal.pone.0350665 (PMC13577461; doi:10.1371/journal.pone.0350665)
Supplement: Supplementary file 3 [file pone.0350665.s004.docx]

**S1 Table: Characteristics of included studies.**

| First Author | Year | Country | Title | Data Source | Study Design | References |
| --- | --- | --- | --- | --- | --- | --- |
| Euro-Peristat | 2025 | Europe | Euro-Peristat: Indicators of Perinatal Health | Medico-admin Database | Observational | 9 |
| Agency for Healthcare Research and Quality | 2024 | USA | Agency for Healthcare Research and Quality (AHRQ) Quality Indicators for Maternal Health Indicators | Medico-admin Database | Observational | 17 |
| Nijagal | 2018 | USA | Standardized outcome measures for pregnancy and childbirth, an ICHOM proposal | No patient data | Consensus | 20 |
| Foglia | 2015 | USA | Accuracy of the Adverse Outcome Index: An Obstetrical Quality Measure | Medico-admin Database | Validation | 26 |
| Gregory | 2013 | USA | AHRQ patient safety indicators: time to include hemorrhage and infection during childbirth | Medico-admin Database | Observational | 27 |
| Holubkov | 1998 | USA | Analysis, assessment, and presentation of risk-adjusted statewide obstetrical care data: the StORQS  II study in Washington State. Statewide Obstetrics Review and Quality System | Medico-admin Database | Observational | 28 |
| El Haj Ibrahim | 2014 | USA | Anesthesia complications as a childbirth patient safety indicator | Medico-admin Database | Observational | 29 |
| Ueda | 2020 | Japan | Applicability of care quality indicators for women with low-risk pregnancies planning hospital birth: a retrospective study of medical records | Medical Records | Validation | 30 |
| Bottle | 2009 | UK | Application of AHRQ patient safety indicators to English hospital data | Medico-admin Database | Validation | 31 |
| Boesveld | 2018 | Netherlands | An approach to assessing the quality of birth centres results of the Dutch birth centre study | No patient data | Consensus | 32 |
| Grobman | 2006 | USA | Are the Agency for Healthcare Research and Quality obstetric trauma indicators valid measures of hospital safety? | Medico-admin Database | Observational | 33 |
| Mann | 2006 | USA | Assessing quality obstetrical care: development of standardized measures | Medical Records | Consensus/ Validation | 34 |
| Howell | 2014 | USA | Association between hospital-level obstetric quality indicators and maternal and neonatal morbidity | Medico-admin Database | Observational | 35 |
| Crenn-Hébert | 2018 | France | Can hospital discharge data be used for monitoring indicators associated with postpartum hemorrhages? The HERA multicenter observational study | Medico-admin Database | Observational | 36 |
| Sandin-Bojö | 2008 | Sweden | Care in labor: a Swedish survey using the Bologna Score | Medical Records | Observational | 37 |
| Baghurst | 2013 | Australia | The case for retaining severe perineal tears as an indicator of the quality of obstetric care | Medico-admin Database | Observational | 38 |
| Mesterton | 2016 | Sweden | Case mix adjustment of health outcomes, resource use and process indicators in childbirth care: a register-based study | Medico-admin Database | Observational | 39 |
| Goossens | 2015 | Netherlands | Clinical indicators associated with the mode of twin delivery: an analysis of 22,712 twin pairs | Medico-admin Database | Observational | 40 |
| Romano | 2005 | USA | Coding of perineal lacerations and other complications of obstetric care in hospital discharge data | Medico-admin Database | Validation | 41 |
| Thompson | 2018 | Netherlands | Creating an Optimality Index - Netherlands: a validation study | Medical Records | Consensus/ Validation | 42 |
| Andersson | 2016 | Denmark | The Danish national quality database for births | Medico-admin Database | Observational | 43 |
| Kritchevsky | 1999 | International | Definition and adjustment of Cesarean section rates and assessments of hospital performance | Medico-admin Database | Observational | 44 |
| Pyykönen | 2014 | Finland | Determining obstetric patient safety indicators: the differences in neonatal outcome measures between different-sized delivery units | Medico-admin Database | Observational | 45 |
| Boesveld | 2017 | Netherlands | Developing quality indicators for assessing quality of birth centre care: a mixed- methods study | No patient data | Consensus | 46 |
| Patel | 2019 | Oman | Development and Implementation of Maternity Dashboard in Regional Hospital for Quality Improvement at Ground Level: A Pilot Study | Medical Records | Observational | 47 |
| Melman | 2016 | Netherlands | Development and Measurement of Guidelines-Based Quality Indicators of Caesarean Section Care in the Netherlands: A RAND-Modified  Delphi Procedure and Retrospective Medical Chart Review | Medical Records | Consensus/ Validation | 48 |
| Dyess-Nugent | 2020 | USA | Development of a Quality Indicator to Measure Urgent Requests to the Bedside | No patient data | Validation | 49 |
| Shinjo | 2023 | Japan | Development of a set of quality indicators in paediatric and perinatal care in Japan with a modified Delphi method | Medico-admin Database | Consensus/ Validation | 50 |
| Soong | 1999 | Australia | The development of clinical indicators--the impact on midwifery practice in Queensland in the future | Medico-admin Database | Observational | 51 |
| Smit | 2013 | Netherlands | The development of quality indicators for the prevention and management of postpartum haemorrhage in primary midwifery care in the  Netherlands | No patient data | Consensus | 52 |
| Bookhart | 2023 | USA | A dose-response relationship found between the Ten Steps to Successful Breastfeeding indicators and in-hospital exclusive breastfeeding  in US hospitals | Medico-admin Database | Observational | 53 |
| Nair | 2016 | UK | Establishing a National Maternal Morbidity Outcome Indicator in England: A Population-Based Study Using Routine Hospital Data | Medico-admin Database | Observational | 54 |
| Srinivas | 2010 | USA | Evaluating risk-adjusted cesarean delivery rate as a measure of obstetric quality | Medico-admin Database | Observational | 55 |
| Kazandjian | 1989 | USA | Evaluating the appropriateness of care: a study of cesarean section rates | Medical Records | Consensus/ Validation | 56 |
| Friedman | 2015 | USA | Evaluation of third-degree and fourth-degree laceration rates as quality indicators | Medico-admin Database | Observational | 57 |
| Hutcheon | 2015 | Canada | Feasibility of Implementing a Standardized Clinical Performance Indicator to Evaluate the Quality of Obstetrical Care in British Columbia | Medical Records | Validation | 58 |
| de Bruin-Kooistra | 2012 | Netherlands | Finding the right indicators for assessing quality midwifery care | No patient data | Consensus | 59 |
| Korst | 2005 | USA | A Framework for the Development of maternal quality of care indicators | No patient data | Consensus | 60 |
| Woiski | 2015 | Netherlands | Guideline-based development of quality indicators for prevention and management of postpartum hemorrhage | No patient data | Consensus | 61 |
| Korst | 2005 | USA | Hospital rates of maternal and neonatal infection in a low-risk population | Medico-admin Database | Observational | 62 |
| Bamber | 2020 | UK | The identification of key indicators to drive quality improvement in obstetric anaesthesia: results of the Obstetric Anaesthetists' Association/National Perinatal Epidemiology Unit collaborative Delphi project | No patient data | Consensus | 63 |
| Alfirevic | 2004 | UK | The impact of delivery suite guidelines on intrapartum care in 'standard primigravida' | Medical Records | Observational | 64 |
| Gilbert | 2013 | USA | Improving recording accuracy, transparency, and performance for obstetric quality measures in a community hospital-based obstetrics department | Medical Records | Observational | 65 |
| Walker | 2010 | USA | In search of perinatal quality outcome measures: 1 hospital's in-depth analysis of the Adverse Outcomes Index | Medico-admin Database | Validation | 66 |
| Mesterton | 2019 | Sweden | Inter-hospital variations in health outcomes in childbirth care in Sweden: a register-based study | Medico-admin Database | Observational | 67 |
| Sibanda | 2013 | UK | Intrapartum care quality indicators: a systematic approach for achieving consensus | No patient data | Consensus | 68 |
| Maga | 2022 | Italy | The Italian midwifery core outcome set (M-COS) for healthy childbearing women and newborns: Development and initial validation study | No patient data | Consensus | 69 |
| Jayaratnam | 2016 | Australia | Maternal 'near miss' at Royal Darwin Hospital: An analysis of severe maternal morbidity at an Australian regional tertiary maternity unit | Medical Records | Observational | 70 |
| Kesmodel | 2011 | Denmark | Measuring and improving quality in obstetrics--the implementation of national indicators in Denmark | No patient data | Consensus | 71 |
| Sprague | 2013 | Canada | Measuring quality in maternal-newborn care: developing a clinical dashboard | No patient data | Consensus | 72 |
| Peoples-Sheps | 1998 | USA | Model indicators for maternal and child health: an overview of process, product, and applications | No patient data | Consensus | 73 |
| Korst | 2014 | USA | Monitoring childbirth morbidity using hospital discharge data: further development and application of a composite measure | Medico-admin Database | Observational | 74 |
| Boulkedid | 2010 | France | Monitoring healthcare quality in an obstetrics and gynaecology department using a CUSUM chart | Medical Records | Observational | 75 |
| Sauvegrain | 2019 | France | Monitoring quality of obstetric care from hospital discharge databases: A Delphi survey to propose a new set of indicators based on maternal health outcomes | No patient data | Consensus | 76 |
| Roberts | 2009 | Australia | Monitoring the quality of maternity care: how well are labour and delivery events reported in population health data? | Medico-admin Database | Validation | 77 |
| Kogan | 2015 | USA | A New Performance Measurement System for Maternal and Child Health in the United States | No patient data | Observational | 78 |
| Florea | 2010 | Canada | The Ottawa hospital quality incident notification system for capturing adverse events in obstetrics | No patient data | Consensus | 79 |
| Howell | 2013 | USA | Paradoxical trends and racial differences in obstetric quality and neonatal and maternal mortality | Medico-admin Database | Observational | 80 |
| Zeitlin | 2003 | Europe | PERISTAT: indicators for monitoring and evaluating perinatal health in Europe | No patient data | Consensus | 81 |
| Fabricant | 2023 | USA | The positive predictive value of hospital discharge data for identifying severe maternal morbidity with and without blood transfusion | Medico-admin Database | Validation | 82 |
| Smit | 2014 | Netherlands | Postpartum haemorrhage in midwifery care in the Netherlands: validation of quality indicators for midwifery guidelines | No patient data | Validation | 83 |
| Moser | 2022 | Switzerland | Quality aspects relating to giving birth in Switzerland: An analysis of quality indicators in inpatient obstetrics from 2013 to 2017 | Medico-admin Database | Observational | 84 |
| Pileggi | 2019 | Italy | Quality in perinatal care: applying performance measurement using joint commission on accreditation of healthcare organizations indicators in Italy | Medical Records | Observational | 85 |
| Guiguet-Auclair | 2023 | France | Quality Indicators during Delivery and the Immediate Postpartum Period: A Modified Delphi Study | No patient data | Consensus | 86 |
| Boulkedid | 2013 | France | Quality indicators for continuous monitoring to improve maternal and infant health in maternity departments: a modified Delphi survey of an international multidisciplinary panel | No patient data | Consensus | 87 |
| Bailit | 2006 | USA | Quality of obstetric care and risk-adjusted primary cesarean delivery rates | Medico-admin Database | Observational | 88 |
| Rich | 2021 | Canada | Selecting contextually appropriate performance indicators in a circumpolar context: a modified Delphi approach | No patient data | Consensus | 89 |
| Santos | 2013 | Europe | Should European perinatal indicators be revisited? | No patient data | Consensus | 90 |
| Voerman | 2013 | Netherlands | A systematic approach towards the development of a set of quality indicators for public reporting in community-based maternity care | No patient data | Consensus | 91 |
| Clapp | 2019 | USA | Unexpected term NICU admissions: a marker of obstetrical care quality? | Medical Records | Observational | 92 |
| Ueda | 2019 | Japan | Updating quality indicators for low-risk labour care in Japan using current clinical practice guidelines: a modified Delphi method | No patient data | Consensus | 93 |
| Havranek | 2023 | Switzerland | Validity of 16 AHRQ Patient Safety Indicators to identify in-hospital complications: a medical record review across nine Swiss hospitals | Medico-admin Database | Validation | 94 |
| Wagner | 2012 | USA | Comprehensive perinatal safety initiative to reduce adverse obstetric events | Medical Records | Observational | 95 |
| Knight | 2019 | UK | Establishing a composite neonatal adverse outcome indicator using English hospital administrative data | Medico-admin Database | Validation | 96 |
| Lain | 2012 | Australia | Incidence of Severe Adverse Neonatal Outcomes: Use of a Composite Indicator in a Population Cohort | Medico-admin Database | Validation | 97 |
| Fridman | 2021 | USA | Severe Maternal Morbidity in California Hospitals: Performance Based on a Validated Multivariable Prediction Model | Medico-admin Database | Observational | 98 |
| Pyykönen | 2013 | Finland | The rate of obstetric anal sphincter injuries in Finnish obstetric units as a patient safety indicator | Medico-admin Database | Observational | 99 |
| United Kingdom: National Maternity Dashboard | 2025 | UK | United Kingdom: National Maternity Dashboard | Medico-admin Database | Observational | 100 |
| Main | 2016 | USA | Measuring severe maternal morbidity: validation of potential measures | Medico-admin Database | Consensus | 101 |
| Australian Institute of Health and Welfare | 2024 | Australia | Australia: National Core Maternity Indicators | Medico-admin Database | Observational | 102 |
| Canada: Perinatal Health Indicators | 2024 | Canada | Canada: Perinatal Health Indicators | Medico-admin Database | Observational | 103 |
| New Zealand: Maternity Clinical Indicators | 2024 | New Zealand | New Zealand: Maternity Clinical Indicators | Medico-admin Database | Observational | 104 |
| The Joint Commission | 2022 | USA | Joint Commission National Quality Measures (V2022B2) | Medico-admin Database | Observational | 105 |
| Cundiff | 2024 | Canada | Vaginal Uncomplicated Delivery Rate as a Quality Indicator Compared to Cesarean Delivery Rate: A Quantitative Analysis of a Population Database. | Medico-admin Database | Observational | 106 |
