# Supplementary material for: Monitoring childbirth and immediate postpartum care quality: A scoping review of indicators in high-income countries
Source: PLoS One. 2026 Sep 15;21(9):e0350665. doi: 10.1371/journal.pone.0350665 (PMC13577461; doi:10.1371/journal.pone.0350665)
Supplement: Supplementary file 4 [file pone.0350665.s005.docx]

## **S2 Table. Socio-demographic indicators.**

| **Indicator Construct** | **Standardised Indicator** | **Name/ definition of indicator *per source*** | **Numerator *per source*** | **Denominator *per source*** | **References** |
| --- | --- | --- | --- | --- | --- |
| Births | Births | Numbers of babies born, and women delivered | N/A | This indicator is used as the denominator for the others in this article | 68 |
|  | Ethnic minority births | Percentage of deliveries to women from ethnic minority groups | Total number of episodes where the mother's ethnicity is recorded as any value other than 'A','B','C','X','Z','9', where the episode type is '2' (delivery episode) or '5' (other delivery event), and where the actual place of delivery is not '1' (at a domestic address), '5' (in a private hospital) or '6' (in another hospital or institution) or the actual place of delivery is null | Total number of episodes where the mother's ethnicity is recorded as any value other than 'A','B','C','X','Z','9', where the episode type is '2' (delivery episode) or '5' (other delivery event), and where the actual place of delivery is not '1' (at a domestic address), '5' (in a private hospital) or '6' (in another hospital or institution) or the actual place of delivery is null | 100 |
|  | Multiple births | Number of live births and stillbirths following a multiple gestation pregnancy, as a percentage of total births | Number of live births and stillbirths following a multiple gestation pregnancy, in Canada | Total number of hospital live births and stillbirths, in Canada. Excludes live births with unknown plurality. | 103 |
|  |  | Number of maternities where the outcome is a multiple birth expressed as a rate per 1,000 total maternities. | Number of maternities where the outcome is a multiple birth occurring in the respective calendar years. | Total number of maternities occurring in the respective calendar years. | 100 |
|  |  | The number of women in a multiple gestation pregnancy at delivery as a proportion of all women delivering live or stillborn babies by number of fetuses. | The number of women in a multiple gestation pregnancy at delivery | as a proportion of all women delivering live or stillborn babies by number of fetuses. | 81 |
|  |  |  | The number of women in a multiple gestation pregnancy at delivery as a proportion of all women delivering live or stillborn babies | number of fetuses | 9 |
|  |  | Women with multiple births | Women with multiple births | Numbers of babies born, and women delivered | 68 |
|  | Number of births | Number of live births | Number of hospital livebirths, in Canada | N/A | 103 |
|  | Preterm birth | Women delivered ≤37 and ≥34 weeks | Women delivered ≤37 and ≥34 weeks | Numbers of babies born, and women delivered | 68 |
|  | Teenage births | Live births in women aged under 18 per 1,000 females aged 15-17 | Number of live births registered in women aged under 18 years by area of usual residence | Number of women aged 15-17 living in the area | 100 |
| Gestational age | Gestational age | Number of live births and fetal deaths at each completed week of gestation (starting from 22 weeks) expressed as a proportion of all live and stillbirths for singletons and multiples. | Number of live births and fetal deaths at each completed week of gestation (starting  from 22 weeks) | all live and stillbirths | 9 |
|  | Gestational age <32 weeks | Gestational age <32 completed  weeks | N/A | N/A | 96 |
|  |  | Gestational age <32 weeks | N/A | N/A | 97 |
|  | Gestational age distribution | Number of live births and fetal deaths at each completed week of gestation (starting from 22 weeks) expressed as a proportion of all live and stillbirths for singletons and multiples. | Number of live births and fetal deaths at each completed week of gestation (starting from 22 weeks) | expressed as a proportion of all live and stillbirths for singletons and multiples | 81 |
| Parity | Parity | Distribution of parity (number of previous live or stillbirths) of women delivering a live or stillbirth. | N/A | N/A | 9 |
|  |  |  | number of previous live or stillbirths | women delivering a live or stillbirth. | 81 |
| Place of birth | Place of birth | Number of births occurring at home or in maternity units defined by the number of annual births, with the following groups: home, <300, 300-499, 500-999, 1000-1499, 1500-1999, 2000-2999, 3000-3999, 4000-4999, 5000+) | Number of births occurring at home or in maternity units | Number of annual births | 9 |
|  |  | Number of births occurring at home or in maternity units defined by the number of  annual births, with the following groups: home, <300, 300-499, 500-999, 1000-1499, 1500  1999, 2000-2999, 3000-3999, 4000-4999, 5000+ | N/A | N/A | 81 |
| Birthweight | Birthweight distribution | Number of births within each 500g weight interval expressed as a proportion of all live and stillbirths calculated by gestational age and vital status at birth for singletons and multiples. | Number of births within each 500g weight interval | all live and stillbirths | 9 |
|  |  |  |  | expressed as a proportion of all live and stillbirths calculated by gestational age and vital status at birth for singletons and multiples. | 81 |
| Maternal age | Maternal age | Distribution of age in years at delivery for women delivering a live or stillbirth. | Age in years at delivery | women delivering a live or stillbirth | 81 |
|  |  | Distribution of age in years at delivery for women delivering a live or stillbirth.  Recommended presentation, as follows: <15,15-17, 18-19,20-24,25-29,30-34,35-39,40-44,45-49,50+ | N/A | N/A | 9 |
| Smoking status at delivery | Smoking status at delivery | The number of mothers known to be smokers at the time of delivery as a percentage of all maternities with known smoking status. A maternity is defined as a pregnant woman who gives birth to one or more live or stillborn babies of at least 24 weeks gestation, where the baby is delivered by either a midwife or doctor at home or in a NHS hospital | Number of women known to smoke at time of delivery. | Number of maternities where smoking status is known. | 100 |
| Mother's education | Mother's education | The distribution of education level of women delivering live or stillborn babies, expressed as highest level of education of the mother. | Education level of women | all live or stillnborn babies | 9 |
| Household occupation | Household occupation | The distribution of the mother’s and father’s occupation for mothers and fathers of live or stillborn babies using the International Standard Classification of Occupations (ISCO08) major groups:  1-Managers  2-Professionals  3-Technicians and associate professionals  4-Clerical support workers  5-Service and sales workers  6-Skilled agricultural, forestry and fishery workers  7-Craft and related trades workers  8-Plant and machine operators, and assemblers  9-Elementary occupations  0-Armed forces occupations  99– no profession  88– student <http://www.ilo.org/public/english/bureau/stat/isco/>  If countries cannot use this classification, data are provided using local classifications used to  report on occupation in your system | The distribution of the mother’s and father’s occupation for mothers and fathers | all live or stillnborn babies | 9 |
| Mother country of origin | Mother country of origin | Distribution of the countries of birth of women delivering live or stillborn babies. | Distribution of the countries of birth of women | all women delivering live or stillborn babies | 9 |
