# Supplementary material for: Monitoring childbirth and immediate postpartum care quality: A scoping review of indicators in high-income countries
Source: PLoS One. 2026 Sep 15;21(9):e0350665. doi: 10.1371/journal.pone.0350665 (PMC13577461; doi:10.1371/journal.pone.0350665)
Supplement: Supplementary file 5 [file pone.0350665.s006.docx]

## **S3 Table. List of incomplete indicators.**

| Indicator construct | Standardised indicator | Indicator (per source) | References |
| --- | --- | --- | --- |
| Organisation | **(Integrated) ICT system with hospital and midwifery practices** | (Integrated) ICT system with hospital and midwifery practices | 46 |
|  | **(Integrated) IT system with hospital and midwife practices** | (Integrated) IT system with hospital and midwife practices | 32 |
|  | **24 /7 telephone accessibility birth centre** | 24 /7 telephone accessibility birth centre | 32, 46 |
|  | **Access to facilities** | Physical access to birth centre for clients (i.e. parking) | 32 |
|  |  | Physical access to birth centre for midwives and maternity care assistants (e.g. parking) | 32 |
|  |  | Physical access to birthing centre for clients (i.e. parking) | 46 |
|  |  | Physical access to birthing centre for midwives and maternity care assistants (e.g. parking) | 46 |
|  | **Admission agreement for professionals who use birth care facilities at the birth centre** | Admission agreement for professionals who use birth care facilities at the birth centre | 46 |
|  | **Agreements with ambulance service and nearest hospital about urgent referrals** | Agreements with ambulance service and nearest hospital about urgent referrals | 46 |
|  | **Birth centre has vision of birth care** | Birth centre has vision of birth care | 46 |
|  | **Births** | Deliveries | 47 |
|  | **Caesarean section, planned** | Whether there are elective caesarean section lists with dedicated (i.e. not expected to cover emergency work) obstetric, anaesthetic and theatre staff | 63 |
|  | **Care pathways formulated with chain partners** | Care pathways formulated with chain partners | 46 |
|  | **Continuous presence of a healthcare provider during labour** | Continuous presence of a healthcare provider during labour | 32 |
|  | **Cooperation with (almost) all relevant organizations in the region (such as midwifery practices and maternity care assistance organisations)** | Cooperation with (almost) all relevant organizations in the region (such as midwifery practices and maternity care assistance organisations) | 46 |
|  | **Cooperation with (almost) all relevant organizations in the region (such as midwives and maternity care assistance providers)** | Cooperation with (almost) all relevant organizations in the region (such as midwives and maternity care assistance providers) | 32 |
|  | **Coordination of discharge** | Step 10: Care at discharge: Coordination of discharge to ensure appropriate follow-up care | 53 |
|  |  | Step 10: Care at discharge: Coordination of discharge to ensure ongoing breastfeeding support | 53 |
|  | **Detailed business plan for the birth centre** | Detailed business plan for the birth centre | 46 |
|  | **Facilities at a birth centre in relation to emergency care (i.e. CPR resuscitation)** | Facilities at a birth centre in relation to emergency care (i.e. CPR resuscitation) | 32, 46 |
|  | **Facilities at a birth centre in relation to stay (i.e. possibilities for father to stay overnight, private shower and/or toilet in room, bath)** | Facilities at a birth centre in relation to stay (i.e. possibilities for father to stay overnight, private shower and/or toilet in room, bath) | 46 |
|  | **Formal partnership agreement with chain partners** | Formal partnership agreement with chain partners | 46 |
|  | **In case of referral from the birth centre durante partu: guaranteed access to the hospital with which agreements were made** | In case of referral from the birth centre durante partu: guaranteed access to the hospital with which agreements were made | 46 |
|  | **In case of referral from the birth centre durante partu: required time between decision to refer and treatment in hospital** | In case of referral from the birth centre durante partu: required time between decision to refer and treatment in hospital | 46 |
|  | **Independent or freestanding setting with a home-like, non-clinical atmosphere** | Independent or freestanding setting with a home-like, non-clinical atmosphere | 46 |
|  | **Independent supervisory board** | Independent supervisory board | 46 |
|  | **indicator to assess the appropriateness of CS based on clinical factors that justify the procedure.** | The indicator assesses the appropriateness of CS based on key clinical factors, including maternal and fetal health, labor progression, and prior cesarean history. The pilot test is conducted to evaluate the protocol's effectiveness in assessing CS practices, rather than to gather specific information about CS. | 56 |
|  | **Indoor connection between birth centre and hospital** | Indoor connection between birth centre and hospital | 46 |
|  | **Joint use of an electronic care record** | Joint use of an electronic care record | 32 |
|  | **Joint use of an electronic patient record** | Joint use of an electronic patient record | 46 |
|  | **Legal entity (i.e. foundation, association) of the birth centre** | Legal entity (i.e. foundation, association) of the birth centre | 46 |
|  | **Low birthweight** | % live births <1500 g who were delivered at a risk-appropriate facility | 73 |
|  | **Maternity care assistant present during labour** | Maternity care assistant present during labour | 32 |
|  | **Multidisciplinary composition of the board of the birth centre (e.g. midwives, maternity care organization and hospital)** | Multidisciplinary composition of the board of the birth centre (e.g. midwives, maternity care organization and hospital) | 46 |
|  | **Opportunities to stay in the birth centre after giving birth** | Opportunities to stay in the birth centre after giving birth | 46 |
|  | **Participation and representation of clients in organisation (i.e. in the board)** | Participation and representation of clients in organisation (i.e. in the board) | 46 |
|  | **Protocols on care aspects** | Protocols on care aspects | 32 |
|  |  |  | 46 |
|  | **System of quality improvement (i.e. accreditation)** | System of quality improvement (i.e. accreditation) | 32 |
|  | **There are written agreements about collaboration between midwives/ GPs and maternity care assistants** | There are written agreements about collaboration between midwives/ GPs and maternity care assistants | 91 |
|  | **There is a protocol for daily monitoring and reporting of body temperature, miction, and (mal)nutrition of the infant** | There is a protocol for daily monitoring and reporting of body temperature, miction, and (mal)nutrition of the infant | 91 |
|  | **There is a protocol for daily monitoring and reporting of maternal infections** | There is a protocol for daily monitoring and reporting of maternal infections | 91 |
|  | **There is a protocol for monitoring and promoting personal and household hygiene** | There is a protocol for monitoring and promoting personal and household hygiene | 91 |
|  | **There is a protocol for monitoring the emotional status of the mother** | There is a protocol for monitoring the emotional status of the mother | 91 |
|  | **There is a structural regional meeting with hospital care providers** | There is a structural regional meeting with hospital care providers | 91 |
|  | **Transfer from birthing room to a residence room when the mother of newly-born child wants/can/ has to stay a (part of her) childbed period** | Transfer from birthing room to a residence room when the mother of newly-born child wants/can/ has to stay a (part of her) childbed period | 46 |
|  | **Women switched to receive care provided primarily by obstetricians from midwifery ward** | Women switched to receive care provided primarily by obstetricians from midwifery ward | 93 |
|  | **Written agreements on care aspects (i.e. by hospital care, obstetricians)** | Written agreements on care aspects (i.e. by hospital care, obstetricians) | 32, 46 |
| Morbidity, neonatal | **Broncho-pulmonary dysplasia** | Broncho-pulmonary dysplasia | 97 |
|  | **Cerbral infarction** | Cerbral infarction | 97 |
|  | **Cerebral haemorrhage** | Cerebral hemorrhage 348.5,430,431,432,433,434, 436,671.5,674.0,780.01,03 | 74 |
|  | **Cerebral palsy** | Prevalence of Cerebral Palsy | 9 |
|  | **Clear amniotic fluid aspiration with respiratory symptoms** | Clear amniotic fluid aspiration with respiratory symptoms 770.14 | 74 |
|  | **Congenital syphilis** | Congenital syphilis incidence rate | 73 |
|  | **Down Syndrome** | Down Syndrome prevalence rate | 73 |
|  | **Encephalopathy** | Encephalopathy 348.3 | 74 |
|  | **Fetal alcohol syndrome (FAS)** | Fetal alcohol syndrome (FAS) prevalance rate | 73 |
|  | **Hypoxic-Ischemic Encephalopathy** | Hypoxic ischaemic encephalopathy | 96 |
|  |  | Hypoxic ischemic encephalopathy | 97 |
|  |  | Neonatal Birth Weight <2,500 g with Hypoxic-Ischemic Encephalopathy | 26 |
|  | **Intraventricular hemorrhage (grades 2, 3 and 4)** | Intraventricular hemorrhage (grades 2, 3 and 4) | 97 |
|  | **Meconium aspiration with respiratory symptoms** | Meconium aspiration with respiratory symptoms 770.32 | 74 |
|  | **Neonatal Asphyxia Rate** | Hypoxia/asphyxia. Composite morbidity calculated with: Congenital or infantile cerebral palsy 343; Hypoxic ischemic encephalopathy 768.7; Mild or moderate birth asphyxia with/without neurologic involvement 768.6; Severe birth asphyxia with neurologic involvement 768.5; Unspecified birth asphyxia 768.9; Shock, resuscitation, and complications; Disseminated intravascular coagulation 776.2; Necrotizingenterocolitis 777.5; Renal failure 584.5; Shock,hypotension 785.5 | 74 |
|  | **Neonatal morbidity** | Neonatal morbidity | 69 |
|  | **Other respiratory problems after birth** | Other respiratory problems after birth 770.81,2,3,4,6,7,8,9 | 74 |
|  | **Other respiratory: primary atelectasis, respiratory failure** | Other respiratory: primary atelectasis, respiratory failure | 97 |
|  | **Periventricular leukomalacia** | Periventricular leukomalacia | 97 |
|  | **Pneumothorax** | Pneumothorax 770.2 | 74 |
|  | **Pneumothorax requiring an intercostal catheter** | Pneumothorax requiring an intercostal catheter | 97 |
|  | **Respiratory distress syndrome** | Respiratory distress syndrome | 97 |
|  |  | Respiratory distress syndrome 769 | 74 |
|  | **Seizure** | Seizure | 96, 97 |
|  |  | Seizures 345.3,779.0 | 74 |
|  | **Severe fetal hypoxia** | Severe fetal hypoxia | 43 |
|  | **Subarachnoid hemorrhage** | Subarachnoid hemorrhage 772.2 | 74 |
|  | **Transient tachypnea of the newborn (TTN)** | Transient tachypnea of the newborn (TTN) | 92 |
|  |  | Transient tachypnea of the newborn infant 770.6 | 74 |
| Perineal tears | **First degree perineal tears** | First degree perineal tears | 77 |
|  | **Perineal tears** | International definition of perineal tears (first, second, third, and fourth degrees) | 9 |
|  |  | Women with perineal tear and no perineorrhaphy | 93 |
|  | **Perineum, intact** | Percentage of women with intact perineum after vaginal birth | 91 |
|  | **Second degree perineal tears** | Repair 1st, 2nd degree perineal tear | 77 |
|  |  | Second degree perineal laceration | 93 |
|  |  | Second degree perineal tears | 77 |
|  | **Third degree perineal tears** | Third degree perineal tears | 77 |
|  | **Third/fourth degree perineal tears** | 3°- or 4°-perineal tear | 34 |
|  |  | 3rd or 4th degree perineal tear | 58 |
|  |  | 3rd or 4th degree tear after instrumental delivery | 77 |
|  |  | 3rd or 4th degree tear after normal vaginal delivery | 77 |
|  |  | Any 3rd, 4th degree tear or repair | 77 |
|  |  | Lacerations, third and fourth degree | 43 |
|  |  | Obstetric anal sphincter injuries (OASIS) were studied for women who delivered vaginally | 67 |
|  |  | Perineal laceration, 3rd/4th degree 664.2,3 | 74 |
|  |  | Repair 3rd, 4th degree perineal tear | 77 |
|  |  | Severe tearing of the perineum during childbirth, extending to deeper layers such as muscles and tissues | 26 |
|  |  | Third and fourth degree perineal lacerations: Incidence of third- and fourth-degree perineal | 60 |
|  |  | Third or fourth degree perineal laceration | 93 |
|  |  | Third- or fourth-degree perineal laceration | 66 |
| Trauma, neonatal | **Birth trauma** | Birth trauma | 34 |
|  |  | Birth trauma (intracranial haemorrhage paralysis due to brachial plexus injury, skull or long bone fracture) | 96 |
|  |  | Birth trauma (intracranial hemorrhage paralysis due to brachial plexus injury, skull or long bone fracture) | 97 |
|  |  | Birth trauma/injuries | 74 |
|  |  | Inborn birth trauma | 66 |
|  |  | Injury to the infant during delivery | 58 |
|  |  | Injury to the infant during delivery, including nerve damage, fractures, or other physical injuries | 26 |
|  |  | Living infants with birth injuries | 93 |
|  | **Brachial plexus injury** | Brachial plexus injury 767.6 | 74 |
|  | **Clavicle fracture** | Clavicle fracture 767.2 | 74 |
|  | **Erb's paralysis** | Erb's paralysis | 45 |
|  | **Facial nerve injury** | Facial nerve injury 767.5 | 74 |
|  | **Fetus or newborn infant affected by other complications of labor and delivery** | Fetus or newborn infant affected by other complications of labor and delivery 763.0,1,2,3,4,5 | 74 |
|  | **Fracture of clavicula** | Fracture of clavicula | 45 |
|  | **Intraventricular haemorrhage (grades 3 and 4)** | Intraventricular haemorrhage (grades 3 and 4) | 96 |
|  | **Other cranial/peripheral nerves** | Other cranial/peripheral nerves 767.7 | 74 |
|  | **Other skeletal injuries** | Other skeletal injuries 767.3 | 74 |
|  | **Other specified birth trauma** | Other specified birth trauma 767.8 | 74 |
|  | **Pneumothorax requiring intercostal catheter** | Pneumothorax requiring intercostal catheter | 96 |
|  | **Spine/spinal cord injuries** | Spine/spinal cord injuries 767.4 | 74 |
|  | **Subdural/cerebral haemorrhage** | Subdural/cerebral hemorrhage 767.0 | 74 |
|  | **Subgaleal haemorrhage** | Subgaleal hemorrhage 767.11 | 74 |
| Morbidity, maternal | **Acute psychosis** | Acute psychosis 298.10 | 74 |
|  | **Maternal morbidity** | Maternal morbidity | 69 |
|  |  | Other maternal morbidity | 74 |
|  | **Other conditions which required immediate medical or anaesthetic assessment** | Any other conditions which required immediate medical or anaesthetic assessment, for example respiratory/cardiac compromise | 70 |
|  | **Patient experiencing severe shock or unexplained maternal collapse** | Any patient experiencing severe shock or unexplained maternal collapse | 70 |
|  | **Pulmonary embolism** | Pulmonary embolism 415.11,19,673.0,1,2,3,8 | 74 |
|  | **Pulmonary embolus** | Any pulmonary embolus | 70 |
|  | **Severe maternal morbidity** | Severe maternal morbidity based on 20 components, excluding blood transfusion | 98 |
|  |  | Severe maternal morbidity based on 21 components including blood transfusion | 98 |
|  |  | Severe maternal morbidity, with or without blood transfusion. | 82 |
|  | **Severe pre-eclampsia complicated by major morbidity** | Severe pre-eclampsia complicated by HELLP syndrome,eclampsia, acute renal failure (creatinine >300), disseminated intravascular coagulation (DIC) or pulmonary oedema or other major morbidity | 70 |
|  | **Uterine dehiscence** | Uterine dehiscence 674.1 | 74 |
|  | **Uterine rupture** | Uterine rupture | 26, 34, 66 |
|  |  | Uterine rupture 665.0,1 Rupture(allcodes) | 74 |
|  |  | Uterine rupture during labor | 58 |
| Infant feeding | **Breastfeeding** | Breastfeeding initiation | 69 |
|  | **Breastfeeding education/support** | Step 5: Support with breastfeeding: Education/support on assessing effective breastfeeding by observing their newborn’s elimination patterns ≥80% | 53 |
|  |  | Step 5: Support with breastfeeding: Education/support on assessing effective breastfeeding by observing their newborn's latch and the presence of audible swallowing ≥80% | 53 |
|  |  | Step 5: Support with breastfeeding: Education/support on hand expressing breast milk ≥80% | 53 |
|  |  | Step 6: Limited supplementation: Counseling on the risk of infant formula for breastfeeding dyads ≥80% | 53 |
|  |  | Step 8: Responsive feeding: Mothers are taught to breastfeed as long/often as newborn wants ≥80% | 53 |
|  |  | Step 8: Responsive feeding: Mothers are taught to respond to feeding cues ≥80% | 53 |
|  |  | Step 9: Bottles, nipples, and pacifiers: Education and support on risk of teats | 53 |
|  | **Exclusive breastfeeding/breastmilk** | Infants that were fed only breast milk at the time of discharge from the hospital | 93 |
|  |  | Step 1: Hospital policies: Hospital records exclusive breastfeeding throughout entire hospitalization | 53 |
|  | **Formula supplementation** | Infants given formula supplementation without medical rationale from birth to discharge in term infants, even though the woman intended to breastfeed | 93 |
|  | **Infant Formula Marketing and Procurement** | Step 1: Hospital policies: Acquisition of infant formula at fair market price | 53 |
|  |  | Step 1: Hospital policies: Gifts or free samples of infant formula, teats, coupons, educational materials from companies who make/sell infant formula, etc. are not provided | 53 |
|  | **Percent of healthy, term breastfed newborns who are fed infant formula** | Step 6: Limited supplementation: Percent of healthy, term breastfed newborns who are fed infant formula <20% | 53 |
| Mortality, neonatal | **Death (within 28 days of birth or before a discharge home from hospital)** | Death (within 28 days of birth or before a discharge home from hospital) | 97 |
|  | **Infant mortality** | Low birthweight-specific infant mortality rate | 73 |
|  | **Intrapartum or neonatal mortality >2,500 g** | Death of the fetus or neonate during labor or in the hospital if the newborn is at least 2,500 g, at or beyond 37 weeks’ gestation, with no congenital anomalies or fetal hydrops. | 58 |
|  |  | Death of the fetus or neonate during labor or in the neonatal period >2,500 g | 26 |
|  |  | Intrapartum neonatal death ≥2500 g | 66 |
|  |  | Intrapartum or neonatal death > 2,500 g | 34 |
|  | **Mortality Within 27 days** | Mortality Within 27 days (of all deliveries, of all deliveries and fetal deaths, of all deliveries at hospital with neonatal ICU) | 28 |
|  | **Neonatal death within 28 days** | Neonatal death within 28 days (includes deaths after 28 days if the infant was never discharged home) | 96 |
|  | **Neonatal mortality** | Neonatal death | 74 |
|  |  | Neonatal mortality | 69, 89 |
|  |  | Neonatal mortality rate | 73 |
| Resources | **24 h presence of a caregiver in birth centre (maternity care assistant or midwife)** | 24 h presence of a caregiver in birth centre (maternity care assistant or midwife) | 46 |
|  | **Care comprises an expert in the field of lactation** | Care comprises an expert in the field of lactation | 91 |
|  | **Coaching during labour** | Percentage of births during which support during labour was timely (maternity care assistant present within 1 hr after call) | 91 |
|  | **Continuous presence of a healthcare provider during labour** | Continuous presence of a healthcare provider during labour | 46 |
|  | **Continuous support for women in the delivery room** | Continuous support for women in the delivery room | 43 |
|  | **Maternity care assistant present during labour** | Maternity care assistant present during labour | 46 |
|  | **Patient focus** | Focusing on the patients (i.e. use of individual birth plan) | 46 |
|  | **Point‐of‐care testing for estimation of haemoglobin** | Whether point‐of‐care testing is available for estimation of haemoglobin (e.g. Hemocue®, Hemocue AB, Ängelholm, Sweden or blood gas analyser) | 63 |
|  | **Publication of annual report** | Publication of annual report | 46 |
|  | **Staff in the birth centre mirrors the population that (may) use the birth centre** | Staff in the birth centre mirrors the population that (may) use the birth centre | 46 |
|  | **Timeliness of a physician's response to a woman's bedside after a nurse's urgent request** | Timeliness of a physician's response to a woman's bedside after a nurse's urgent request | 49 |
| Apgar score | **Apgar score <4 at 5 minutes** | Apgar <4 at 5 min | 67 |
|  |  | Apgar below 4 at 5 minutes | 45 |
|  | **Apgar score <7 at 5 minutes** | 5-min Apgar score <7 for a non-anomalous infant ≥2500 g at or beyond 37 weeks with no fetal hydrops | 58 |
|  |  | APGAR < 7 at 5 minutes | 26, 34 |
|  |  | Apgar 5 <7 for bodyweight ≥2500 g | 66 |
|  |  | Apgar below 7 at 5 minutes | 45 |
|  |  | Percentage of neonates with APGAR score <7 at 5 mins after birth | 91 |
|  |  | Term infants with Apgar score less than 7 at 5 min after birth | 93 |
|  | **Apgar score at 5 minutes** | Apgar score at 5 min | 69 |
|  |  | Distribution of APGAR score at 5 minutes | 81 |
| Management, pph | **Hysterectomy** | Any emergency/unplanned peripartum hysterectomy, regardless of number of units transfused (includes all placenta accretas) | 101 |
|  |  | Hysterectomy 683,684,685,686,687,689 | 74 |
|  | **Obstetric haemorrhage management** | Obstetric hemorrhage with <4 units of blood products transfused and evidence of pulmonary congestion that requires >1 dose of Lasix | 101 |
|  |  | Obstetric hemorrhage with >=4 units of red blood cells transfused | 101 |
|  |  | Obstetric hemorrhage with 2 units of red blood cells and 2 units of fresh frozen plasma transfused (without other procedures or complications), if not judged to be “over- exuberant” transfusion | 101 |
|  |  | Obstetric hemorrhage with uterine artery embolization, regardless of number of units transfused | 101 |
|  |  | Obstetric hemorrhage with uterine balloon or uterine compression suture placed and 2-3 units of blood products transfused | 101 |
|  | **Postpartum haemorrhage management** | Any postpartum haemorrhage (PPH) transferred to OT | 70 |
|  |  | Obstetric tamponade of uterus or vagina 75.8 | 74 |
|  | **Prevention of postpartum haemorrhage** | Women receiving uterotonics for the prevention of postpartum haemorrhage during the third stage of labour | 93 |
| Icu admission | **Arrhythmia that requires intensive care unit with further treatments** | Arrhythmia that requires intensive care unit with further treatments | 101 |
|  | **ICU admission** | Additional potential maternal intensive care unit admission (diagnosis codes) | 74 |
|  |  | Additional potential maternal intensive care unit admission (procedure codes) | 74 |
|  |  | Admission of the mother to the ICU due to obstetrical complications (based on use of maternal intensive care procedures such as intubation or ventilation as a proxy) | 58 |
|  |  | Any intensive care unit admission that includes treatment or diagnostic or therapeutic procedure | 101 |
|  |  | Any patient transferred to ICU | 70 |
|  |  | Maternal admission to ICU | 26, 34 |
|  |  | Unplanned maternal admission to intensive care unit | 66 |
|  | **Obstetric haemorrhage admitted to intensive care unit for invasive monitoring or treatment (either medication or procedure; not just observed**  **overnight)** | Obstetric hemorrhage admitted to intensive care unit for invasive monitoring or treatment (either medication or procedure; not just observed  overnight) | 101 |
| Caesarean section | **Caesarean section rate** | Cesarean section rate | 47 |
|  |  | Lower segment caesarean section | 77 |
|  |  | Women with a term, singleton infant in vertex position delivered by caesarean section | 93 |
|  | **Caesarean section rate, during labour** | Classical cesarean section during labour | 77 |
|  | **Caesarean section, emergency** | Cesarean section – grade 1 with delivery within 15 minutes | 43 |
|  |  | Cesarean section – grade 2 with delivery within 30 minutes | 43 |
|  |  | Emergency cesarean section | 77 |
|  | **Caesarean section, planned** | Classical cesarean section before labour | 77 |
|  |  | Elective cesarean section | 77 |
| Trauma, maternal | **Bladder laceration** | Bladder laceration 665.5 | 74 |
|  | **High vaginal laceration** | High vaginal laceration 665.4 | 74 |
|  | **Obstetric haematoma** | Obstetric hematoma 664.5,665.7 | 74 |
|  | **Other lacerations** | Other lacerations 664.4,8,9,665.8,9 | 74 |
|  | **Repair of bladder/urethra** | Repair of bladder/urethra 75.61 | 74 |
|  | **Repair of cervical or uterine laceration** | Repair of cervical or uterine laceration 75.51,52 | 74 |
|  | **Repair of rectum or sphincter** | Repair of rectum or sphincter 75.62 | 74 |
|  | **Repair of small intestine** | Repair of small intestine 46.73,75 | 74 |
| Infection, neonatal | **Bacterial meningitis** | Bacterial meningitis | 96 |
|  | **Congenital pneumonia** | Congenital pneumonia 770.0 | 74 |
|  | **Pneumonia** | Pneumonia | 97 |
|  | **Sepsis** | Sepsis | 92 |
|  | **Sepsis/septicaemia** | Sepsis/septicaemia (streptococcus, staphylococcus, E. coli, unspecified Gram-negative) | 97 |
|  |  | Sepsis/septicaemia (Streptococcus, Staphylococcus, Escherichia coli, unspecified Gram negative) | 96 |
|  |  | Septicemia of newborn infant 771.81 | 74 |
|  | **Severe sepsis** | Severe sepsis 995.92 | 74 |
| Mortality, maternal | **Maternal mortality** | Death of the mother during delivery admission | 58 |
|  |  | death of the mother during or shortly after childbirth | 26 |
|  |  | Maternal death | 34, 66, 74 |
|  |  | Maternal mortality | 69, 89 |
| Labour, management | **Assessment during first stage labour** | Assessment during first stage labour: (1) 8-hourly temperature and blood pressure, (2) half‑hourly frequency of contractions and foetal heart rate, (3) vaginal examination 4-hourly or if there is concern about progress or in response to the woman’s wishes, (4) woman’s emotional and psychological needs, including her desire for pain relief | 93 |
|  | **Assessment during second stage labour** | Assessment during second stage labour: (1) 1-hourly blood pressure and woman’s heart rate, (2) half-hourly frequency of contractions, (3) half-hourly foetal heart rate, (4) frequency of passing urine, (5) vaginal examination 1-hourly or if there is concern about progress or in response to the woman's wishes, (6) woman’s emotional and psychological needs | 93 |
|  | **Augmentation of labour** | Any augmentation of labour | 77 |
|  |  | Augmentation of labour by artificial rupture of membranes | 77 |
|  |  | Augmentation of labour by oxytocin | 77 |
|  | **Comfortable positions throughout second stage labour** | Women having been encouraged and supported to adopt the most comfortable positions throughout second stage labour | 93 |
|  | **Initial assessment at admission** | Initial assessment of labour risk at admission: (1) measuring foetal heart rate more than 20 min, (2) vaginal examination, (3) frequency of construction, (4) woman’s emotional and psychological needs, (5) a part and level of pain including her desire for pain relief, (6) foetal movement | 93 |
|  | **Partogram use** | The percentage of births during which a partogram was used for monitoring and observation | 91 |
| Evaluation | **Participation of birth centre in local maternity care consultation and cooperation group (VSV)** | Participation of birth centre in local maternity care consultation and cooperation group (VSV) | 46 |
|  | **Participation of the birth centre in scientific research** | Participation of the birth centre in scientific research | 46 |
|  | **Peer review of severe adverse events with medical staff** | Peer review of severe adverse events with medical staff | 93 |
|  | **Structural evaluation of the care provided in the birth centre** | Structural evaluation of the care provided in the birth centre | 32 |
|  | **Structural research on client experiences** | Structural research on client experiences | 46 |
|  | **System of quality improvement (i.e. accreditation)** | System of quality improvement (i.e. accreditation) | 46 |
|  | **Women and infants having complete medical records based on all quality indicator** | Women and infants having complete medical records based on all quality indicator | 93 |
| Onset of labour | **Induction of labour** | Any induction of labour | 77 |
|  |  | Induction of labor | 47 |
|  |  | Induction of labour by artifical rupture of membranes | 77 |
|  |  | Induction of labour by oxytocin | 77 |
|  |  | Induction of labour by prostaglandins | 77 |
|  |  | Women with a term, singleton infant in vertex position delivered by labour induction | 93 |
|  | **Post-term deliveries** | Proportion of post-term deliveries | 45 |
| Resuscitation | **Cardiopulmonary resuscitation** | Cardiopulmonary resuscitation 99.60 | 74 |
|  | **Japanese Neonatal Resuscitation Algorithm** | Infants offered the necessary resuscitation in the first minutes after birth, evaluating their condition in line with the Japanese Neonatal Resuscitation Algorithm | 93 |
|  | **Respiratory support** | Respiratory support: Resuscitation for asphyxiated term neonate with low oxygen concentrations and oxygen saturation measured by pulse oximetry immediately after birth | 93 |
|  | **Resuscitation** | Resuscitation | 97 |
|  |  | Resuscitation (intubation and/or chest compression) | 96 |
|  | **Ventilatory support** | Ventilatory support (mechanical ventilation and/or CPAP) | 96 |
|  | **Ventilatory support (mechanical ventilation and/or CPAP)** | Ventilatory support (mechanical ventilation and/or CPAP) | 97 |
| Delivery, instrumental | **Failed forceps** | Failed forceps | 77 |
|  | **Failed vacuum** | Failed vacuum | 77 |
|  | **Instrument delivery** | Forceps | 77 |
|  |  | Instrumental deliveries | 47 |
|  |  | Vacuum | 77 |
|  |  | Women with a term, singleton infant in vertex position delivered by instrument delivery | 93 |
| Delivery, vaginal | **Vaginal birth after caesarean** | Trial of labor after cesarean delivery | 92 |
|  | **Vaginal delivery** | Delivery of a healthy child after an uncomplicated birth | 43 |
|  |  | Normal birth: low-risk pregnancy with spontaneous onset of labor that remaining so throughout labor and birth | 69 |
|  |  | Spontaneous vaginal delivery | 77 |
|  |  | Women with a term, singleton infant in vertex position delivered by vaginal delivery | 93 |
|  | **Vaginal delivery in MLU** | Women planning spontaneous vaginal birth in a midwifery ward, and being able to follow that plan | 93 |
| Blood transfusion | **Blood transfusion** | Blood transfusion | 34 |
|  |  | Blood transfusion for maternal hemorrhage during or after childbirth. | 58 |
|  |  | Maternal blood transfusions | 66 |
|  |  | The need for the mother to receive a blood transfusion due to excessive blood loss during or after childbirth | 26 |
|  |  | Transfusion (procedure codes) 99.0 99.00,03,04 (transfusion whole blood or packed red blood cells) | 74 |
|  |  | Transfusion of blood or blood products | 97 |
| Mortality, foetal | **Foetal mortality** | Fetal mortality rate | 73 |
|  | **Stillbirth** | Number of stillbirths | 103 |
|  |  | Stillbirth < 22 weeks’ gestation | 69 |
|  |  | Stillbirth ≥ 22 weeks’ gestation | 69 |
|  |  | Stillbirths | 89 |
| Infection, maternal | **Infection with hypotension with multiple liters of intravenous fluid or pressors used (septic shock)** | Infection with hypotension with multiple liters of intravenous fluid or pressors used (septic shock) | 101 |
|  | **Infection with pulmonary complications such as pulmonary edema or acute respiratory distress syndrome** | Infection with pulmonary complications such as pulmonary edema or acute respiratory distress syndrome | 101 |
|  | **Maternal infection** | Maternal infection 658.4,659.2,3,670,672.2 | 74 |
|  | **Maternal pneumonia** | Maternal pneumonia 481,482,485,486 | 74 |
|  | **Postpartum infection** | Infection were studied for women with 84 days follow-up | 67 |
| Birthweight | **Intrauterine growth restriction** | Intrauterine growth restriction (IUGR): The proportion of newborns whose birth weight by gestational age is <10th percentile (small for gestational age) undiagnosed prior to delivery | 60 |
|  | **Large for gestational age** | Birthweight more than 4500 g | 45 |
|  | **Low birthweight** | Low birth weight | 89 |
|  | **Low birthweight, <1,500 g** | Birth weight <1500 g | 96, 97 |
| Anaesthesia complication | **Anaesthesia complication** | Anesthesia complication 349.0,3,668 668.0,668.1,668.2 | 74 |
|  | **Aspiration pneumonia** | Aspiration pneumonia | 101 |
|  | **Dural puncture** | The percentage of women who had an epidural (or a combined spinal epidural) for labour analgesia who had an accidental dural puncture | 63 |
|  | **Epidural hematoma** | Epidural hematoma | 101 |
|  | **Total spinal anesthesia** | Total spinal anesthesia | 101 |
| Nicu admission | **NICU admission** | NICU admissions | 89 |
|  | **NICU admission >2,500g > 24 hours** | Admission to NICU >2,500 g & for > 24 hours | 34 |
|  | **NICU admission for ≥2 Days or Transfer within 24 Hours of Birth to a facility with a NICU** | Neonatal care unit admission (NICU) ≥2 days or transfer within 24 h of birth to a facility with a NICU for a non- anomalous infant ≥2500 g at or beyond 37 weeks with no fetal hydrops | 58 |
|  | **NICU admission, term** | Admission to neonatal intensive care unit >1 day, bodyweight ≥2500 g and gestational age ≥37 wks | 66 |
|  |  | Term NICU admissions: Rates and indications for term neonatal intensive care unit (Neonatal Intensive Care Unit) admissions | 60 |
| Treatment, maternal | **Return to OR** | Any postnatal patient transferred to OT following delivery | 70 |
|  |  | Maternal return or operating room/labor and delivery | 66 |
|  |  | Obstetric hemorrhage with return to operating room for any major procedure (excludes dilation) | 101 |
|  |  | Return to OR / labor & delivery | 34 |
|  |  | Return to OR for maternal complications | 26 |
| Early skin-to-skin contact | **Early skin-to-skin contact** | Establishment of skin-to-skin contact between the mother and the newborn infant | 43 |
|  |  | Women having early skin-to-skin contact with their babies if they wish, soon after birth in secure surroundings | 93 |
|  | **Uninterrupted skin-to-skin contact until the first breastfeeding is completed after Cesarean delivery ≥80%** | Step 4: Care right after birth: Uninterrupted skin-to-skin contact until the first breastfeeding is completed after Cesarean delivery ≥80% | 53 |
|  | **Uninterrupted skin-to-skin contact until the first breastfeeding is completed after vaginal delivery ≥80%** | Step 4: Care right after birth: Uninterrupted skin-to-skin contact until the first breastfeeding is completed after vaginal delivery ≥80% | 53 |
| Training | **Interdisciplinary training** | Joint (interdisciplinary) emergency care training | 32 |
|  |  | Multidisciplinary education as result of formulated points of improvement from perinatal audit | 32 |
|  |  |  | 46 |
|  | **Joint (interdisciplinary) emergency care training** | Joint (interdisciplinary) emergency care training | 46 |
| Postpartum haemorrhage (pph) | **Haemorrhage >1000 ml** | Haemorrhage was studied for women with 14 days follow-up | 67 |
|  | **Postpartum haemorrhage** | Postpartum haemorrhage more than 500 g within 2 h of birth | 93 |
|  |  | Postpartum hemorrhage 666.0,1,2,3 | 74 |
|  | **Postpartum haemorrhage ≥1000 ml** | Postpartum hemorrhage | 43 |
| Rooming-in practices | **Infants remain in mothers’ room during procedures such as pediatric exams, hearing screening, routine labs, etc.** | Step 7: Rooming-in: Infants remain in mothers’ room during procedures such as pediatric exams, hearing screening, routine labs, etc. | 53 |
|  | **Newborns who room with their mothers for at least 23 hours per day ≥80%** | Step 7: Rooming-in: Newborns who room with their mothers for at least 23 hours per day ≥80% | 53 |
|  | **Observation of mother-infant dyads to ensure safety** | Step 7: Rooming-in: Observation of mother-infant dyads to ensure safety | 53 |
|  | **Vaginally-delivered newborns separated from their mothers before starting rooming-in <20%** | Step 7: Rooming-in: Vaginally-delivered newborns separated from their mothers before starting rooming-in <20% | 53 |
| Cord blood analysis | **Arterial cord pH** | pH below 6.95 | 45 |
|  |  | pH below 7.00 | 45 |
|  |  | pH below 7.05 | 45 |
|  |  | pH below 7.10 | 45 |
| Anaesthesia/pain relief | **Anaesthesia/pain relief** | Anesthesia/pain relief | 43 |
|  | **Analgesia conversation** | Percentage of women with whom possibilities with regard to pain reduction during labour have been discussed | 91 |
|  | **Effective analgesia** | The percentage of epidurals for labour analgesia that provided adequate pain relief within 45 min of placement (from the start of epidural insertion) | 63 |
| Prematurity | **Preterm birth** | Percentage of very preterm babies delivered in units without a NICU | 9 |
|  |  | Percentage of very preterm births delivered in units without a NICU | 81 |
|  |  | Preterm birth | 89 |
| Intervention, neonatal | **Any intravenous fluids** | Any intravenous fluids | 97 |
|  | **Cardiac arrest of newborn infant** | Cardiac arrest of newborn infant 427.5,779.85 | 74 |
|  | **Non invasive mechanical ventilation without (delivery through) endotracheal tube or tracheostomy** | Non invasive mechanical ventilation without (delivery through) endotracheal tube or tracheostomy 93.90 | 74 |
| Hospitalization, neonatal | **Admission to paediatrics department within a week after birth** | Infants admission to paediatrics department within a week after birth | 93 |
|  | **Neonatal length of stay** | Neonatal length of stay | 69 |
|  | **Neonatal length of stay >5d** | Neonatal length of stay >5d | 74 |
| Mortality, perinatal | **Perinatal mortality** | Perinatal death rate: Incidence of stillbirth and neonatal death | 60 |
|  |  | Perinatal deaths | 89 |
|  |  | Perinatal mortality rate | 73 |
| Treatment, neonatal | **Any intravenous fluids** | Any intravenous fluids | 96 |
|  | **Therapeutic hypothermia** | Therapeutic hypothermia | 96 |
| Chorioamnionitis | **Chorioamnionitis** | Chorioamnionitis: Incidence of chorioamnionitis by hospital discharge code, or by histopathology | 60 |
|  |  | Presence of chorioamnionitis | 92 |
| Referral to hospital | **Percentage of neonates referred to hospital within 24 hrs after birth for other reasons than congenital abnormalities** | Percentage of neonates referred to hospital within 24 hrs after birth for other reasons than congenital abnormalities | 91 |
|  | **Percentage of women referred to hospital within 24 hrs after birth** | Percentage of women referred to hospital within 24 hrs after birth | 91 |
| Necrotising enterocolitis | **Necrotising enterocolitis** | Necrotising enterocolitis | 96, 97 |
| Pneumonia | **Pneumonia** | Pneumonia | 92, 96 |
| Intervention, complications | **Bowel or bladder injury during surgery beyond minor serosal tear** | Bowel or bladder injury during surgery beyond minor serosal tear | 101 |
|  | **Surgical, bladder, and bowel complications** | Surgical, bladder, and bowel complications | 101 |
| Complications, maternal | **Unanticipated Operative Procedure** | Unanticipated operative procedure for maternal complications | 58 |
|  | **Wound complication** | Wound complication 674.2,3 | 74 |
| Anaesthesia services | **Anaesthesia availability** | Anesthesia services: Availability of 24 h in-house anesthesia services | 60 |
|  |  | Facilities at a birth centre in relation to pain management (i.e. nitrous oxide) | 46 |
| Composite indicator | **Bologna Score** | A score of five infers that the birth has been managed according to the best available evidence for care in normal birth | 37 |
|  | **Optimality index** | Optimality, as a concept in perinatal health care, can be defined as the maximal perinatal outcome, with minimal intervention, placed within the context of the woman’s social, medical, and obstetric history. | 42 |
| Eclampsia | **Eclamptic seizure(s) or epileptic seizures** | Eclamptic seizure(s) or epileptic seizures that were “status | 101 |
|  | **Preeclampsia with difficult to control severe hypertension** | Preeclampsia with difficult to control severe hypertension (>160 systolic blood pressure or >110 diastolic blood pressure) that requires multiple intravenous doses and/or persistent >=48 hours after delivery | 101 |
| Hospitalization, maternal | **Length of stay >5d** | Length of stay >5d | 74 |
|  | **Maternal length of stay** | Maternal length of stay | 69 |
| Access to facilities | **Distance between birth centre and hospital** | Distance between birth centre and hospital | 46 |
|  | **Necessary transport time from birth centre to hospital** | Necessary transport time from birth centre to hospital | 46 |
| Newborn transfer | **Newborn transfer** | Neonatal transfer to other acute care hospital Disposition | 74 |
|  |  | Newborn transfer (inappropriate level of service for newborns): newborns < 1500 g transferred from the hospital of birth to an NICU | 60 |
| Arrhythmia | **Arrhythmia that requires >1 dose of intravenous medication but not intensive care unit admission** | Arrhythmia that requires >1 dose of intravenous medication but not intensive care unit admission | 101 |
| Surgical procedure | **Any body cavity surgical procedure** | Any body cavity surgical procedure | 97 |
| Respiratory support | **Respiratory support** | Respirator treatment | 45 |
| Central venous or arterial catheter | **Central venous or arterial catheter** | Central venous or arterial catheter | 97 |
| Anaemia | **Anaemia** | Anemia | 92 |
| Manual removal of the placenta | **Manual removal of the placenta** | Manual removal of the placenta | 77 |
| Respiratory complications | **Diagnosis of acute respiratory distress syndrome, pulmonary edema, or postoperative pneumonia** | Diagnosis of acute respiratory distress syndrome, pulmonary edema, or postoperative pneumonia | 101 |
| Nonresponsiveness or loss of vision | **Nonresponsiveness or loss of vision** | Nonresponsiveness or loss of vision, permanent or temporary (but not momentary), documented in physician’s progress note | 101 |
| Mortality, near miss | **Near miss/mortality** | Near miss/mortality | 47 |
| Obstetric shock | **Obstetric shock** | Obstetric shock 669.1,2 | 74 |
| Communication | **Women having a review of their childbirth experience and support with the midwives and other staff who assisted at the birth** | Women having a review of their childbirth experience and support with the midwives and other staff who assisted at the birth | 93 |
| Oliguria | **Oliguria treatment** | Oliguria treated with multiple doses of Lasix | 101 |
| Prolonged ileus for>=4 days | **Prolonged ileus for>=4 days** | Prolonged ileus for>=4 days | 101 |
| Maternal transfer | **Maternal transfer to other acute care hospital** | Maternal transfer to other acute care hospital Disposition on the discharge diagnosis record | 74 |
| Falls | **Women having a fall during their hospitalization** | Women having a fall during their hospitalization | 93 |
| Episiotomy | **Episiotomy** | Episiotomy | 77 |
| Respiratory distress syndrome (rds) | **Respiratory distress syndrome (RDS)** | Respiratory distress syndrome (RDS) | 92 |
| Meconium-stained amniotic fluid | **Meconium-stained amniotic fluid** | Presence of meconium-stained amniotic fluid | 92 |
| Haemorrhage | **Obstetrical haemorrhage** | Obstetrical hemorrhage: Incidence of obstetrical hemorrhage as defined by 1) 10% change in hematocrit between admission and the postpartum period; or 2) Need for erythrocyte transfusion | 60 |
| Peripartum cardiomyopathy | **Peripartum cardiomyopathy** | Peripartum cardiomyopathy | 101 |
| Screening, maternal | **Depression screening** | Women being screened for antenatal or postnatal depression using a validated questionnaire | 93 |
| Labour | **Spontaneous labour** | Spontaneous labour | 77 |
| Dvt or pulmonary embolism | **Deep vein thrombosis or pulmonary embolism** | Deep vein thrombosis or pulmonary embolism | 101 |
| Policies | **Structural evaluation of the care provided in the birth centre** | Structural evaluation of the care provided in the birth centre | 46 |
| Intervention, maternal | **Continuous infusion (intravenous drip) of an antihypertensive medication** | Continuous infusion (intravenous drip) of an antihypertensive medication | 101 |
| Coagulation abnormalities | **Multiple coagulation abnormalities or severe HELLP syndrome** | Multiple coagulation abnormalities or severe hemolysis, elevated liver enzymes, and low platelet count (HELLP) syndrome | 101 |
| Liver complications | **Liver or subcapsular hematoma or severe liver injury admitted to the intensive care unit (bilirubin >6 or liver enzymes >600)** | Liver or subcapsular hematoma or severe liver injury admitted to the intensive care unit (bilirubin >6 or liver enzymes >600) | 101 |
| Abruption | **Abruption (Intrapartum)** | Intrapartum abruption | 92 |
| Low risk mother | **Low risk at the end of pregnancy** | Definition of low risk at the end of pregnancy | 9 |
