# Supplementary material for: Monitoring childbirth and immediate postpartum care quality: A scoping review of indicators in high-income countries
Source: PLoS One. 2026 Sep 15;21(9):e0350665. doi: 10.1371/journal.pone.0350665 (PMC13577461; doi:10.1371/journal.pone.0350665)
Supplement: Supplementary file 6 [file pone.0350665.s007.docx]

**S4 Table. List of maternal outcome indicators (N=132).**

| **Indicator Constructs***  **Maternal** | **Standardised Indicators^$^** | **Name/ definition of indicator *per source*** | **Numerator *per source*** | **Denominator *per source*** | **References** |
| --- | --- | --- | --- | --- | --- |
| **Mortality, maternal** | Maternal mortality | Maternal mortality ratio by cause of death | Number of maternal deaths (during pregnancy or within 42 days following the end of pregnancy) | Per 100,000 live births | (9) |
|  |  | Number of maternal deaths per 1000 births | Number of maternal deaths | Per 1000 births | (91) |
|  |  | Maternal mortality ratio by maternal age | The number of maternal deaths (the death of a woman while pregnant or within 42 days of the termination of pregnancy, irrespective of the duration and site of the pregnancy, for any cause related to or aggravated by the pregnancy or its management, but not from accidental or incidental causes) | Per 100 000 live births | (9) |
|  |  | Maternal mortality ratio by age, mode of delivery | The number of maternal deaths (the death of a woman while pregnant or within 42 days of the termination of pregnancy, irrespective of the duration and site of the pregnancy, for any cause related to or aggravated by the pregnancy or its management, but not from accidental or incidental causes) | Per 100 000 live births | (81) |
|  |  | Death of a female from any cause related to or aggravated by pregnancy or its management (excluding accidental or incidental causes) | Number of maternal deaths | Total number of live births | (20) |
|  |  | Death of mother during or after childbirth | Maternal deaths | Total deliveries | (106) |
|  |  | Inpatient maternal mortality | Diagnosis-Related Group (DRG) 370–375 and disposition equal to died | All deliveries | (80) |
|  |  | Maternal death | Number of pregnancies complicated by this measure | Total number of deliveries for that time period | (95) |
| **Mortality, maternal**  *(continued)* | Maternal mortality  *(continued)* | Maternal mortality rate | Maternal mortality rate | All women | (76) |
|  |  | Maternal mortality rate among women at low risk of mortality (singleton at term, no abnormal placental insertion, no history of cesarean, no preeclampsia, and no chronic maternal disease) | Maternal mortality rate among women at low risk of mortality (singleton at term, no abnormal placental insertion, no history of cesarean, no preeclampsia, and no chronic maternal disease) | Women at low risk of mortality (singleton at term, no abnormal placental insertion, no history of cesarean, no preeclampsia, and no chronic maternal disease) | (76) |
|  |  | Maternal mortality ratio | Number deaths due to pregnancy or within 42 days of termination of pregnancy from any cause related to or aggravated by the pregnancy but not accidental or incidental causes | Number of live births | (73) |
|  |  | Maternal mortality by cause of death | Number of maternal deaths (during pregnancy or within 42 days following the end of pregnancy) | Per 100,000 live births by the following causes:  Ectopic pregnancy o00  Pregnancy with abortive outcome (excluding ectopic) o01- o08 hypertensive disorders o10- o16 haemorrhage o20 o44.1 o45 o46 o67 o72  Chorioamnionitis/sepsis o75.2 o75.3 o85 o86 o41.1  Other thromboembolic causes o22.2 o22.3 o22.5 o22.8 o22.9, o87.0 o87.1 o87.3 o87.8 o87.9 o88 (excl. O88.1)  Amniotic fluid embolism o88.1  Complications of anaesthesia o29,o74,o89  Uterine rupture o71.0, o71.2  Other direct causes  All direct causes in o chapter that are not listed above | (81) |
|  |  | Maternal mortality by cause of death | Number of maternal deaths (during pregnancy or within 42 days following the end of pregnancy) | Indirect causes: diseases of the circulatory system O99.4  Indirect causes:  Other O98, O99.1-3, 5-9  Unspecified obstetric cause/Unknown O95 | (81) |
| **Morbidity, maternal** | Acute Myocardial Infarction | Myocardial infarction during or after delivery | Discharges with acute myocardial infarction ICD-10 codes | All deliveries identified by ICD-10-CM/PCS codes for delivery | (17) |
|  | Acute renal failure | Acute kidney failure associated with delivery | Discharges with acute renal failure ICD-10 codes | All deliveries, identified by any listed  ICD-10-CM diagnosis code for delivery  ICD-10-PCS procedure code for delivery | (17) |
|  | Amniotic Fluid Embolism | Amniotic fluid embolism during labor or postpartum | Discharges with amniotic fluid embolism ICD-10 codes | All deliveries | (17) |
|  | Cardiac Arrest/Ventricular Fibrillation | Cardiac arrest or ventricular fibrillation during childbirth | Discharges with cardiac arrest/ventricular fibrillation ICD-10 codes | All deliveries | (17) |
|  | Postpartum pulmonary embolism rate | Postpartum pulmonary embolism rate | Postpartum pulmonary embolism rate | All women | (76) |
|  | Pulmonary Edema | Pulmonary edema/acute heart failure in childbirth | Discharges with pulmonary edema ICD-10 codes | All deliveries | (17) |
|  | Severe maternal morbidity | R6. Prevalence of severe maternal morbidity | Severe acute morbidity resulting during pregnancy, delivery or the puerperium period (<42 days) | All women delivering live or stillborn births | (9) |
|  |  | Rate of severe maternal morbidity | Number of disease-specific, intervention-specific, or organ dysfunction-based morbidities associated with delivery, in Canada | Total number of hospital deliveries for women, in Canada. | (103) |
|  |  | Rate of severe maternal morbidity cases | Severe maternal morbidity cases | Total births | (90) |
|  |  | Refined Severe Maternal Morbidity and mortality per 10,000 deliveries | Discharges with refined severe maternal morbidity ICD-10 codes or in-hospital death (DISP=20) | All deliveries identified by ICD-10-CM/PCS codes for delivery | (17) |
|  |  | Severe maternal morbidity | Severe maternal morbidity | Deliveries | (35) |
|  |  | Severe Maternal Morbidity and mortality per 10,000 deliveries | Discharges with severe maternal morbidity ICD-10 codes or in-hospital death (DISP=20) | All deliveries identified by ICD-10-CM/PCS codes for delivery | (17) |
|  |  | Severe Maternal Morbidity per 10,000 deliveries | Discharges with listed ICD-10-CM diagnosis or procedure codes for severe maternal morbidity | All deliveries identified by ICD-10-CM/PCS codes for delivery | (17) |
| **Morbidity, maternal**  *(continued)* | Uterine rupture | Instances of uterine rupture during labor, often associated with VBAC or prolonged labor | Number of uterine ruptures | Total deliveries involving VBAC | (79) |
|  |  | Tearing of uterine wall during labour | Cases of rupture (ICD code O71.1) | Total vaginal deliveries or vbacs | (106) |
|  |  | Uterine rupture | Number of pregnancies complicated by this measure | Total number of deliveries for that time period | (95) |
|  |  | Uterine rupture | Number of women with uterine rupture | Total number of women delivered | (87) |
|  |  | Uterine rupture rate among women with a previous cesarean | Uterine rupture rate | Women with a previous cesarean | (76) |
|  |  | Uterine ruptures | Number of complete or subperitoneal uterine ruptures (dehiscences excluded) | Total number of deliveries | (86) |
| **Composite indicator** | Maternal morbidity outcome indicator (EMMOI) | The EMMOI incorporates a range of relevant maternal morbidity, such as sepsis, eclampsia, and uterine rupture, derived from routine hospital data. The aim is to create a single, reliable measure to monitor maternal morbidity outcomes during childbirth. The indicator is adapted from an Australian model and refined to address specific maternal health concerns and data quality issues in the UK context. | Number of women experiencing at least one of the morbidity events defined in the EMMOI. | Total number of childbirth episodes recorded in the Hospital Episode Statistics (HES) database. | (54) |
| **Postpartum haemorrhage (PPH)** | Postpartum haemorrhage | Incidence of postpartum hemorrhage as a complication during or after delivery | Number of postpartum hemorrhage cases | Total deliveries | (79) |
|  |  | Post operative hemorrhage and hematoma | Number of cases of postpartum hemorrhage coded with appropriate ICD-9-CM (666, 674.3) | Number of all deliveries (vaginal or cesarean) | (27) |
|  |  | Severe maternal bleeding after birth | Number of PPH cases | Total number of deliveries | (20) |
| **Postpartum haemorrhage (PPH)** *(continued)* | Postpartum haemorrhage >500 ml | Postpartum haemorrhage more than 500 g within 2 h of birth | Postpartum haemorrhage more than 500 g within 2 h of birth | Women had vaginal deliveries | (30) |
|  |  | Postpartum hemorrhages >500 mL for vaginal delivery | Number of postpartum hemorrhages > 500 ml for vaginal delivery | Total number of vaginal deliveries | (86) |
|  |  | PPH incidence rate for vaginal deliveries acertained by the number of discharge summaries for deliveries with immediate PPH (blood loss > 500 mL in the 24 hours after delivery) according to CCAM procedure codes. | Number of discharge summaries for deliveries with immediate PPH (blood loss > 500 ml in the 24 hours after delivery) according to CCAM procedure codes. | Vaginal deliveries | (36) |
|  |  | Rate of significant postpartum blood loss | Number of cases with blood loss >500ml | Total standard primigravidae | (64) |
|  | Postpartum haemorrhage with retained products | Postpartum Hemorrhage (PPH) and Retained Products | Number of cases of PPH and retained products. | Total deliveries. | (41) |
|  | Postpartum haemorrhage ≥1000 ml | Incidence of hemorrhage greater than 1000 ml post-birth | Number of deliveries with hemorrhage > 1000 ml | Total number of deliveries | (39) |
|  |  | Mothers with primary PPH > 1000 ml | Mothers with primary PPH > 1000 ml | Numbers of babies born, and women delivered | (68) |
|  |  | Postpartum hemorrhage > 1000 mL for vaginal delivery | Number of postpartum hemorrhages > 1000 ml for vaginal delivery | Total number of vaginal deliveries | (86) |
|  |  | Postpartum hemorrhages > 1000 mL (regardless of mode of delivery) | Number of postpartum hemorrhages > 1000 ml for delivery | Total number of deliveries | (86) |
|  |  | Postpartum hemorrhages > 1000 mL for cesarean deliveries | Number of postpartum hemorrhages > 1000 ml for cesareans | Total number of cesarean deliveries | (86) |
|  |  | PPH incidence rate for cesarean deliveries acertained by the number of discharge summaries for deliveries with immediate PPH (blood loss > 1000 mL in the 24 hours after delivery), according to CCAM procedure codes. | The number of discharge summaries for deliveries with immediate PPH (blood loss > 1000 ml in the 24 hours after delivery), according to CCAM procedure codes. | Caesarean deliveries | (36) |
|  |  | Proportion of women with post-partum hemorrhage ≥1000ml within 2 hours of birth. Standard: Maximum 4% | Women with post-partum hemorrhage ≥1000ml | All deliveries | (71) |
| **Postpartum haemorrhage (PPH)** *(continued)* | Severe postpartum haemorrhage | Severe postpartum hemorrhage | Number of cases | Time period | (47) |
| **Infection, maternal** | Postpartum infection | Incidence of postpartum infection within 12 weeks of childbirth | Number of women diagnosed with infection (cystitis, endometritis, or other) post-birth | Total number of deliveries | (39) |
|  |  | Infection, focusing on postpartum complications. | Number of recorded cases of each infection type in postpartum hospital records. | Total postpartum women discharged from the hospital. | (41) |
|  |  | Rate of maternal infections postpartum, including genitourinary and wound infections | Number of mothers diagnosed with infections postpartum | Total number of mothers delivering in the hospital | (88) |
|  | Sepsis | PSI 13: postoperative sepsis | Number of cases of postpartum infection coded with ICD-9-CM (670, 659.3) | Number of all deliveries (vaginal or cesarean) | (27) |
|  |  | Sepsis during or after delivery | Discharges with sepsis ICD-10 codes | All deliveries | (17) |
|  |  | Severe infection during or after childbirth | Number of sepsis cases | Total number of deliveries | (20) |
|  | Infection, post-intervention | Nosocomial infection of the surgical site | Number of women with nosocomial infections | Number of women who had surgery | (87) |
|  |  | Rate of post-cesarean surgical site infections, including endometritis | Rate of post-cesarean surgical site infections, including endometritis | All women | (76) |
| **Trauma, maternal** | Haematomas | Hematomas | Number of cases | Time period | (47) |
| **Obstruction of labour** | Any obstruction of labour (among women who laboured) | Any obstruction of labour (among women who laboured) | Any obstruction of labour | Women who laboured | (77) |
|  | Obstruction of labour due to malposition (among women who laboured) | Obstruction of labour due to malposition (among women who laboured) | Obstruction of labour due to malposition | Women who laboured | (77) |
|  | Obstruction of labour due to malpresentation (among women who laboured) | Obstruction of labour due to malpresentation (among women who laboured) | Obstruction of labour due to malpresentation | Women who laboured | (77) |
| **Obstruction of labour**  *(continued)* | Shoulder dystocia | Obstruction of labour due to shoulder dystocia (among women who laboured) | Obstruction of labour due to shoulder dystocia | Women who laboured | (77) |
|  |  | Shoulder dystocia | Number of cases | Time period | (47) |
| **Anaesthesia complication** | Anesthesia-related pulmonary aspiration | Rate of anesthesia-related pulmonary aspiration | Rate of anesthesia-related pulmonary aspiration | All women | (76) |
|  |  | Rate of other anesthesia-related pulmonary complications | Rate of other anesthesia-related pulmonary complications | All women | (76) |
|  | Reaction to spinal/epidural | 349.0: reaction to spinal/epidural | Reaction to spinal or spinal headache | All delivery discharges as defined by MDC 14, stratified by delivery type. Cesarean deliveries were defined by MS-DRG 765, and vaginal deliveries were defined by MS-DRG 766, 767, 768, 774, and 775. | (30) |
|  | Spinal or epidural anesthesia headaches with blood patch | Rate of headaches induced by spinal or epidural anesthesia and for which a blood patch was performed | Rate of headaches induced by spinal or epidural anesthesia and for which a blood patch was performed | All women | (76) |
| **Eclampsia** | Eclampsia | Diagnosis of eclampsia at birth admission | Total number of people diagnosed with eclampsia during birth admission | Total number of people giving birth | (104) |
|  |  | Eclampsia | Number of cases | Time period | (47) |
|  |  | Eclampsia (of all deliveries) | Eclampsia | Of all deliveries | (28) |
| **Perineal tears** | Perineal tears | Prevalence of tears to the perineum | The number of women with first-, second-, third-, or fourth-degree of the perineum, separated by instrumental and non-instrumental deliveries. | All women with vaginal delivery | (9) |
|  |  | Perineal Lacerations (of all deliveries) | Perineal lacerations | Of all deliveries | (28) |
|  |  | Second degree perineal laceration | Second degree perineal laceration | Women had vaginal deliveries | (30) |
| **Perineal tears**  *(continued)* | Perineal tears  *(continued)* | Women with perineal tear and no perineorrhaphy | Women with perineal tear and no perineorrhaphy | Women had vaginal deliveries | (30) |
|  | Perineum, intact | Incidence of an intact lower genital tract for multiparous mothers in public accommodation aged between 20-34 with a SVD of a singleton, term infant | The number of multiparous mothers in public accommodation aged between 20-34 with an SVD of a singleton, term infant with an intact lower genital tract. | The number of multiparous mothers in public accommodation aged between 20-34 with an SVD of a singleton, term infant. | (51) |
|  |  | Incidence of an intact lower genital tract for primiparous mothers in public accommodation aged between 20-34 with a SVD of a singleton, term infant | The number of primiparous mothers in public accommodation aged between 20-34 with an SVD of a singleton, term infant with an intact lower genital tract. | The number of primiparous mothers in public accommodation aged between 20-34 with an SVD of a singleton, term infant. | (51) |
|  |  | Intact perineum | Number of women with an intact perineum (without suture) | Total number of deliveries | (86) |
|  |  | Intact perineum | Number of women with intact perineum | Total number of women who delivered vaginally | (87) |
|  |  | Standard primiparae with an intact lower genital tract (no first- to fourth-degree tear or episiotomy) | Total number of standard primiparae with an intact lower genital tract with vaginal birth | Total number of standard primiparae who give birth vaginally | (104) |
|  | Third/fourth degree perineal tears | 3rd or 4th degree tears following vaginal delivery | 3rd or 4th degree tears following vaginal delivery | Numbers of babies born, and women delivered | (68) |
|  |  | Incidence of third- or fourth-degree perineal tears in vaginal deliveries | Number of vaginal deliveries resulting in 3rd or 4th degree perineal tears | Total number of vaginal deliveries | (39) |
|  |  | Number of severe perineal tears | Number of third/fourth-degree tears | Total number of women | (75) |
|  |  | O70.2 OASIS 3rd degree sphincter rupture (all subclasses pooled in this code) | Women with 3rd degree OASIS | Total vaginal deliveries | (45) |
|  |  | O70.3 OASIS 4th degree sphincter rupture | Women with 4th degree OASIS | Total vaginal deliveries | (45) |
|  |  | Perineal laceration incidence for 3rd and 4th degrees in vaginal births | Births with 3rd/4th-degree tear | Total vaginal birth | (84) |
| **Perineal tears**  *(continued)* | Third/fourth degree perineal tears *(continued)* | Third and fourth degree tears for vaginal births | (a) The number of females who had a third or fourth degree perineal laceration after giving birth for the first time and birth vaginally. | (a) The number of females who gave birth for the first time and birth vaginally. | (102) |
|  |  | Third and fourth degree tears for vaginal births | (b) The number of females who had a third or fourth degree perineal laceration after giving birth vaginally. | (b) The number of females who gave birth vaginally. | (102) |
|  |  | Proportion of women with first time-deliveries having 3rd or 4th degree lacerations. Standard: Maximum 6% | Women with 3rd or 4th degree lacerations at first time deliveries | All first time vaginal deliveries | (71) |
|  |  | Instrument-Assisted Vaginal Delivery | Number of third- and fourth-degree lacerations or other trauma recorded per 1000 instrument-assisted vaginal deliveries | Total number of days while women admitted for birth | (33) |
|  |  | Spontaneous vaginal delivery | Number of third- and fourth-degree lacerations or other trauma recorded per 1000 spontaneous vaginal deliveries | Total number of spontaneous vaginal deliveries in a given population or hospital | (33) |
|  |  | Rate of severe perineal lacerations (third- and fourth-degree) | Rate of severe perineal lacerations (third- and fourth-degree) | Women with a spontaneous vaginal delivery | (76) |
|  |  | Rate of severe perineal lacerations (third- and fourth-degree) among all women | Rate of severe perineal lacerations (third- and fourth-degree) among all women | All women | (76) |
|  |  | Rate of severe tears that extend through the anal mucosa. | Number of women with fourth-degree perineal lacerations recorded in hospital discharge data. | Total number of vaginal deliveries recorded in the hospital data | (41) |
|  |  | Rate of severe tears that extend to the anal sphincter. | Number of women with third-degree perineal lacerations recorded in hospital discharge data. | Total number of vaginal deliveries recorded in the hospital data | (41) |
|  |  | Rate of third-degree perineal tears among vaginal deliveries | Number of vaginal deliveries resulting in third-degree tears | Total number of vaginal deliveries in the hospital | (88) |
|  |  | Rate of vaginal lacerations-tears that occur during childbirth involving the anal sphincter. | Number of vaginal deliveries with third-degree lacerations. | Total number of vaginal deliveries. | (57) |
|  |  | Rate of vaginal lacerations-tears that occur during childbirth involving the rectal mucosa | Number of vaginal deliveries with fourth-degree lacerations. | Total number of vaginal deliveries. | (57) |
|  |  | Severe perineal tears | Number of women with 3rd- and 4th-degree perineal tears in vaginal delivery | Total number of vaginal deliveries | (86) |
|  |  | Severe perineal tears for forceps delivery | Number of women with 3rd- and 4th-degree perineal tears in instrumental vaginal delivery using forceps | Total number of instrumental vaginal deliveries using forceps | (86) |
| **Perineal tears**  *(continued)* | Third/fourth degree perineal tears *(continued)* | Severe perineal tears for instrumental vaginal delivery | Number of women with 3rd- and 4th-degree perineal tears for instrumental vaginal delivery | Total number of instrumental vaginal deliveries (forceps, vacuum, spatula) | (86) |
|  |  | Severe perineal tears for non-instrumental vaginal delivery | Number of women with 3rd- and 4th-degree perineal tears in non-instrumental vaginal delivery | Total number of non-instrumental vaginal deliveries | (86) |
|  |  | Severe vaginal/perineal laceration during vaginal birth | Cases with 3° or 4° tears | All vaginal births | (106) |
|  |  | Standard primiparae sustaining a third- or fourth-degree perineal tear and no episiotomy | Total number of standard primiparae sustaining a third- or fourth-degree perineal tear and no episiotomy with vaginal birth | Total number of standard primiparae who give birth vaginally | (104) |
|  |  | Third and fourth degree perineal lacerations after instrumental delivery | Third and fourth degree perineal lacerations per 1000 deliveries with instrument | Vaginal deliveries with instrument | (80) |
|  |  | Third and fourth degree perineal lacerations after spontaneous delivery | Third and fourth degree perineal lacerations per 1000 deliveries without instrument | Vaginal deliveries without instrument | (80) |
|  |  | Third- and fourth degree tears | Women with third- and fourth degree tears | Women with vaginal births | (38) |
|  |  | Third- and fourth-degree lacerations during Cesarean delivery | Cases of third- or fourth-degree lacerations | Total Cesarean births | (31) |
|  |  | Third- and fourth-degree lacerations with instrument | Cases of third- or fourth-degree lacerations | Total vaginal births with instrument use | (31) |
|  |  | Third- and fourth-degree lacerations without instrument | Cases of third- or fourth-degree lacerations | Total vaginal births without instrument use | (31) |
|  |  | Third or fourth degree perineal laceration | Third or fourth degree perineal laceration | Women had vaginal deliveries | (30) |
|  |  | Third/fourth-degree perineal tear | Number of cases | Time period | (47) |
|  |  | Third/fourth-degree perineal tear (full-thickness tears) | Number of women with third/fourth-degree perineal tears | Total number of women who delivered vaginally | (87) |
| **Prematurity, maternal** | Spontaneous Premature Labor | Rate of premature labor cases among low-risk women | Number of premature labor cases | Total women followed from first trimester | (75) |
| **Prematurity, maternal**  *(continued)* | Iatrogenic prematurity | Iatrogenic prematurity | Number of pregnancies complicated by this measure | Total number of deliveries for that time period | (95) |
| **ICU admission, maternal** | ICU admission | Discharge from delivery unit by transfer to adult intensive care unit | Number of women discharged from the delivery unit by a transfer to an intensive care unit | Total number of deliveries | (86) |
|  |  | ICU admission due to pregnancy-related complications | Number of maternal ICU admissions | Total number of deliveries | (20) |
|  |  | Intensive care unit admission | Number of cases | Time period | (47) |
|  |  | Maternal admission to ICU during/after delivery | ICU admissions | All deliveries | (106) |
|  |  | Maternal ICU transfer and/or admission | Number of women transferred and/or admitted to ICU | Total number of women delivered | (87) |
|  |  | Number of women admitted to the ICU | Number of ICU admissions | Total number of women | (86) |
|  |  | Women admitted to ICU from obstetrics | Women admitted to ICU from obstetrics | Numbers of babies born, and women delivered | (68) |
| **Hospitalization** | Long Length of Stay (LOS) | Proportion of mothers experiencing a prolonged hospital stay post-delivery (vaginal: >3 days; cesarean: >5 days) | Number of mothers with prolonged LOS post-delivery | Total number of mothers delivering in the hospital | (88) |
|  | Admitted to higher level of care | Admitted to higher level of care | Number of pregnancies complicated by this measure | Total number of deliveries for that time period | (95) |
| **Treatment, maternal** | Return to operating room | Postpartum return to operating room or L&D for re-treatment | Readmissions to OR/L&D | All deliveries | (106) |
|  |  | Return to the operating room | Number of pregnancies complicated by this measure | Total number of deliveries for that time period | (95) |
| **Maternal readmission rate** | Maternal readmission rate | Hospital readmission within 42 days postpartum | Number of postpartum readmissions | Total number of deliveries | (20) |
|  |  | Maternal readmission rate after postpartum discharge, within 42 days | Maternal readmission rate after postpartum discharge, within 42 days | All women | (76) |
| **Complications, maternal** | Complications | Rate of other complications resulting from care | Rate of other complications resulting from care | All women | (76) |
|  | Severe Medical Illness | Severe maternal medical complications requiring urgent intervention during childbirth | Number of severe maternal medical illnesses | Total deliveries | (79) |
| **Falls** | Women having a fall during their hospitalization | Women having a fall during their hospitalization | Women having a fall during their hospitalization | Total number of days while women admitted for birth | (30) |
| **Drug Reactions** | Drug Reactions | Maternal reactions to drugs administered during labor, including anesthesia-related complications | Number of reported drug reactions | Total deliveries | (79) |
| **Thromboembolic events** | Thromboembolic events | Cases of deep vein thrombosis | Number of thromboembolic cases | Total postpartum women discharged from the hospital. | (41) |

***Indicator constructs:** Umbrella concepts aggregating multiple related indicator groups. **^$^Standardised indicator labels:** Primary analytical units clustering individual indicators with closely related definitions.
