# Supplementary material for: Monitoring childbirth and immediate postpartum care quality: A scoping review of indicators in high-income countries
Source: PLoS One. 2026 Sep 15;21(9):e0350665. doi: 10.1371/journal.pone.0350665 (PMC13577461; doi:10.1371/journal.pone.0350665)
Supplement: Supplementary file 7 [file pone.0350665.s008.docx]

**S5 Table. List of foetal and neonatal outcome indicators (N = 118).**

| **Indicator Constructs***  **Foetal, neonatal** | **Standardised Indicators^$^** | **Name/ definition of indicator *per source*** | **Numerator *per source*** | **Denominator *per source*** | **References** |
| --- | --- | --- | --- | --- | --- |
| **Mortality, foetal** | Foetal mortality | Fetal mortality rate by gestational age, birthweight, plurality | The number of fetal deaths at or after 22 completed weeks of gestation in a given year | Per 1000 live and stillbirths in the same year | (81) |
|  |  | Fetal mortality rate by gestational age, birth weight, plurality | The number of fetal deaths at or after 22 completed weeks of gestation in a given year | Per 1000 live and stillbirths in the same year calculated by gestational age, birth weight and plurality. | (9) |
|  |  | Fetal mortality rate | Crude number of registered stillbirths in Canada with definition based on: birth weight ≥500g or gestational age ≥20 weeks. Includes fetal deaths with missing birth weight or gestational age | Total number of live births and stillbirths in Canada | (103) |
|  |  | Fetal Mortality (of all deliveries and fetal deaths) | Fetal mortality | Of all deliveries and fetal deaths | (28) |
|  | Stillbirth | Fetal death after 28 weeks gestation | Number of stillbirths | Total number of births | (20) |
|  |  | Stillbirth | Number of cases | Time period | (47) |
|  |  |  | Number of pregnancies complicated by this measure | Total number of deliveries for that time period | (95) |
|  |  | Stillbirth rate | Total number of stillbirths (fetal deaths occurring after 24 weeks of gestation) for all maternal ages occurring in the respective calendar years. | Total number of live and stillbirths occurring in the respective calendar years | (100) |
|  |  | Stillbirth with diabetes | Number of cases | Time period | (47) |
|  |  | Stillbirths | Number of fetal or immediate neonatal deaths (including fetal deaths in utero, medical terminations of pregnancy, and intrapartum deaths) | Total number of births (live-born or stillborn infants) | (86) |
|  |  | Stillbirths (per 1000 births) | Number of stillbirths | Total number of births | (45) |
|  |  | Total stillbirths | Term intrapartum stillbirths | Numbers of babies born, and women delivered | (68) |
| **Mortality, neonatal** | Early neonatal mortality | Early neonatal death | Number of cases | Time period | (47) |
|  |  | Early neonatal death | Number of deaths in the first week of life | Number of live births | (45) |
|  | Neonatal mortality | Neonatal mortality rate by gestational age, birthweight, plurality | The number of neonatal deaths (day 0 through 27) after live birth in a given year | Per 1000 live births in the same year | (81) |
|  |  | Neonatal mortality rate by gestational age, birth weight, plurality | The number of neonatal deaths (day 0 through 27) after live birth in a given year | Per 1000 live births in the same year calculated by gestational age, birth weight and plurality. | (9) |
|  |  | Neonatal mortality | Number of deaths of live born infants in the first 0-27 days | Total number of live births, in Canada. | (103) |
|  |  | Death of a live-born neonate up to 28 days of life | Number of neonatal deaths | Total number of live births | (20) |
|  |  | Inpatient neonatal mortality | Inpatient neonatal mortality per 1000 newborn admissions. Identification of newborns admissions with disposition equal to died for newborns up to one year of life, which has been recommended to avoid bias from not including deaths who are hospitalized for long periods and for whom death is related to perinatal causes | Same algorithm as recommended by AHRQ for identification of live newborns | (80) |
|  |  | Intrapartum or neonatal death in infants >2500g | Deaths of neonates >2500g | Total live births >2500g | (106) |
|  |  | Neonatal death | Number of pregnancies complicated by this measure | Total number of deliveries for that time period | (95) |
|  |  | Neonatal mortality | Number of deaths in the 28 days of life | Number of live births | (45) |
|  |  | Number of neonatal deaths (>37 weeks of gestation) within 24 hrs after birth per 1000 births | Number of neonatal deaths (>37 weeks of gestation) within 24 hrs after birth | Per 1000 births | (91) |
|  |  | Neonatal mortality rate broken down by gestational age groups | Neonatal deaths by gestational age | Total live births | (90) |
| **Mortality, infant** | Infant mortality | Infant mortality rate by gestational age, birth weight, plurality | The number of deaths (day 0-364) after live birth in a given year | Per 1000 live births in the same year calculated by gestational age, birth weight and plurality. | (9) |
|  |  | Infant mortality rate by gestational age, birthweight, plurality | The number of deaths (day 0-364) after live birth in a given year | Per 1000 live births in the same year calculated by gestational age, birth weight and plurality. | (81) |
|  |  | Crude mortality rate | Infant mortality numerator: Number of deaths of live born infants in the first year after birth, in Canada. | Total number of live births, in Canada. | (103) |
|  |  | Extremely low birth weight-specific infant mortality rate | Number deaths of infants weighing < 1000 g at birth | Number of live births | (73) |
|  |  | Infant mortality rate | Number infant deaths <1 year of age | Number of live births | (73) |
| **Mortality, perinatal** | Perinatal mortality | Number of perinatal deaths in women starting labour in primary care | Number of perinatal deaths for births starting in primary care | Total births managed initially in primary care | (59) |
|  |  | Number of perinatal deaths reported to the multidisciplinary perinatal mortality audit | Number of perinatal deaths | Total number of live births | (59) |
|  |  | Perinatal mortality | Number of stillbirths and deaths in the first week of life | Total number of births | (45) |
|  |  | Perinatal deaths attributed to congenital anomalies subdivided by gestational age and plurality | The number of deaths in the fetal and neonatal period attributed to congenital anomalies and those attributed to remaining causes | All fetal and neonatal deaths | (9) |
| **Morbidity, neonatal** | Congenital anomalies | Prevalence of selected congenital anomalies | The number of neural tube defects, oral clefts, and Down’s syndrome | All live births, fetal deaths and induced abortions collected in the following categories:  Anencephaly and similar anomalies (icd10-q00)  Spina bifida (icd10-q05)  All congenital anomalies of the central nervous system (icd10-q00-q07)  Down's syndrome (icd10-q90)  Cleft lip (icd10-q36)  Cleft palate with cleft lip (icd10-q37)  Cleft palate (without cleft lip) (icd10-q35) | (9) |
| **Morbidity, neonatal**  *(continued)* | Congenital anomalies *(continued)* | Prevalence of selected congenital anomalies (Down’s syndrome, Neural tube defects) | The number of neural tube defects, oral clefts, and Down’s syndrome | As a proportion of all live births, fetal deaths and induced abortions collected in the following categories: Anencephaly and similar anomalies (ICD10-Q00)  Spina bifida (ICD10-Q05)  All congenital anomalies of the central nervous system (ICD10-Q00-Q07)  Down's syndrome (ICD10-Q90)  Cleft lip (ICD10-Q36)  Cleft palate with cleft lip (ICD10-Q37)  Cleft palate (without cleft lip) (ICD10-Q35) | (81) |
|  | Neural tube defects rate | NTD prevalence rate | Number live births with NTD | Number of live births | (73) |
|  | Severe neonatal morbidity, NICU | Severe health complications in neonates requiring NICU | Severe morbidity cases in NICU | Total NICU cases | (90) |
|  | Severe Neonatal morbidity, term | Severe neonatal morbidity in singleton, term, non-anomalous babies | Severe neonatal morbidity in singleton, term, non-anomalous babies | Deliveries of singleton, term, non-anomalous babies | (35) |
|  | Severe fetal hypoxia | Proportion of liveborn children with neonatal hypoxia Standard: Maximum 1% | Liveborn children with severe neonatal hypoxia | All deliveries | (71) |
|  | Hypoxic-Ischemic Encephalopathy | Birth trauma hypoxic-ischemic encephalopathy | Number of pregnancies complicated by this measure | Total number of deliveries for that time period | (95) |
|  | Neonatal Asphyxia Rate | Perinatal asphyxia | Number of cases | Time period | (47) |
|  |  | Rate of newborns diagnosed with asphyxia during delivery or hospital stay | Number of newborns diagnosed with asphyxia | Total number of newborns delivered in the hospital | (88) |
|  | Meconium aspiration syndrome | Meconium aspiration syndrome | Number of cases | Time period | (47) |
|  | Massive Aspiration Syndrome | Massive Aspiration Syndrome (of all deliveries) | Massive aspiration syndrome | Of all deliveries | (28) |
|  | Neonatal Near Miss | Incidents involving abnormal fetal heart rate or other critical conditions requiring urgent intervention | Number of neonates in near-miss events | Total deliveries | (79) |
| **Trauma, neonatal** | Birth trauma | Birth trauma | Discharges with ICD-10 codes for birth trauma (P10–15) and intraventricular nontraumatic haemorrhage (P52) | All deliveries with a newborn weighting more than 2000 g | (45) |
|  |  | Birth Trauma (of all deliveries) | Birth trauma | Of all deliveries | (28) |
|  |  | Birth Trauma | Cases with birth trauma | Total deliveries | (94) |
|  |  | Injury to neonate | ICD-9 codes: 7670, 7671, 7673, 7674, 7675, 7677, 7678 | All liveborn births (newborns) with these ICD-9 codes: V3000, V3001, V3100, V3101, V3200, V3201, V3300, V3301, V3400, V3401, V3500, V3501, V3600, V3601, V3700, V3701, V3900, V3901 or with specified newborn admission codes and age at admission equal to zero days | (80) |
|  |  | Instances of physical trauma to neonates during birth, such as injuries from assisted deliveries | Number of neonates experiencing birth trauma | Total deliveries | (79) |
|  |  | Living infants with birth injuries | Living infants with birth injuries | Living infants | (30) |
|  |  | Neonatal injury due to birth trauma | Number of neonates with birth injuries | Total number of live births | (20) |
|  |  | Physical injury to neonate during delivery | Cases with trauma (e.g., fractures, nerve injury) | Total births | (106) |
|  | Birth trauma without instrument | Obstetric trauma without instrument | Cases with obstetric trauma without instrument | Total vaginal deliveries without instrument | (94) |
|  | Brachial plexus injury | Number of neonates with brachial plexus injury | Number of brachial plexus injuries | Total number of neonates | (75) |
|  | Obstetric trauma with instrument | Obstetric trauma with instrument | Cases with obstetric trauma with instrument | Total vaginal deliveries with instrument | (94) |
|  | Pneumothorax or hemothorax | Pneumothorax or hemothorax due to the insertion of catheter for central intravenous injection | Number of children aged ≤18 who inserted catheter for central intravenous injection and had pneumothorax or hemothorax | Number of children aged ≤18 who inserted catheter for central intravenous injection | (50) |
| **Cord blood analysis** | Arterial pH < 7.0 | Arterial pH < 7.0 | Number of live-born infants with umbilical artery ph <7.0 | Total number of live-born infants with umbilical artery ph measured | (86) |
|  | Arterial pH < 7.10 | Arterial pH < 7.10 | Number of live-born infants with umbilical artery ph <7.10 | Total number of live-born infants with umbilical artery ph measured | (86) |
| **Birthweight** | Intrauterine growth restriction | Birth weights < 3rd percentile for gestational age | Number of infants with a birth weight < 3rd percentile for gestational age | Total number of newborns | (86) |
|  |  | Proportion of babies who are identified as having severe FGR amongst all babies born at or after 40 weeks gestation | Number of babies (live and stillborn) born at 40 to 43 weeks gestation who weighed less than the third centile for gestational age and sex at birth. | Total number of babies born at 40 to 43 weeks gestation | (102) |
|  |  | Proportion of babies with severe FGR who are born at or after 40 weeks gestation. | Number of babies (live and stillborn) born at 40 to 43 weeks gestation who weighed less than the third centile for gestational age and sex at birth. | Total number of babies born at 40 to 43 weeks gestation | (102) |
|  | Small-for-Gestational-Age | Proportion of Small-for-Gestational-Age | Number of live births in Canada, where birth weight is below the 10th percentile for sex and gestational-specific birth weights | Total number of hospital singleton live births, in Canada. Excludes live births with unknown gestational age or birth weight, live births with gestational age <22 weeks or >43 weeks, and multiple births. | (103) |
|  |  | Small babies at term (37–42 weeks’ gestation) | Total number of babies born at 37–42 weeks’ gestation with birthweight under the 10th centile for their gestation | Total number of babies born at 37–42 weeks’ gestation | (104) |
|  |  | Small babies at term born at 40–42 weeks’ gestation | Total number of babies born at 40–42 weeks’ gestation with birthweight under the 10th centile for their gestation | Total number of babies born at 37–42 weeks’ gestation with birthweight under the 10th centile for their gestation | (104) |
|  |  | Percentage of neonates small for gestational age | Number of neonates classified as small for gestational age | Total number of births | (59) |
|  | Low birthweight, <2,500 g  Low birthweight, <2,500 g  *(continued)* | Low birth weight of all babies | Number of live and still births occurring in the respective calendar year with birthweights under 2500 grams | Number of live and still births occurring in the respective calendar year with a stated birthweight. | (100) |
| **Birthweight**  *(continued)* |  | Low birth weight of term babies | Number of live births at term (>= 37 gestation weeks) with low birth weight (<2500g) | Number of live births at term (>= 37 weeks) with recorded birth weight | (100) |
|  |  | Low-Birthweight (< 2,500 grams) Infant Delivery by Medical Induction of Labor (of all induced procedures without medical indications) | Low-Birthweight (< 2,500 grams) Infant Delivery | All induced procedures without medical indications | (28) |
|  |  | Percent term low birth weight | Number live births weighing <2500 g and >37 completed weeks gestation at birth | Number of live births | (73) |
|  | Low birthweight, <1,800 g | Very Low-Birthweight (< 1,800 grams) Infant Delivery (of deliveries at hospitals without neonatal ICU) | Very Low-Birthweight (< 1,800 grams) Infant Delivery | Deliveries at hospitals without neonatal ICU | (28) |
|  | Low birthweight, <1,500 g | Very low birth weight of all babies | Number of live and still births occurring in the respective calendar year with birthweights under 1500 grams | Number of live and still births occurring in the respective calendar year with a stated birthweight. | (100) |
|  | Low birthweight, <1,000 g | Percent extremely low birth weight | Number live births weighing <1000 g at birth | Number of live births | (73) |
|  | Large for gestational age | Proportion of Large-for-Gestational-Age | Number of live births in Canada, where birth weight is above the 90th percentile for sex and gestational-specific birth weights | Total number of hospital singleton live births, in Canada. Excludes live births with unknown gestational age or birth weight, live births with gestational age <22 weeks or >43 weeks, and multiple births. | (103) |
|  | Low birthweight, <2,500 g, planned CS | Low-Birthweight (< 2,500 grams) Infant Delivery by Planned Repeat CSEC (of all planned repeat CSECs without medical indication) | Low-Birthweight (< 2,500 grams) Infant Delivery by Planned Repeat CSEC | All planned repeat csecs without medical indication | (28) |
| **Apgar score**            **Apgar score**  *(continued)* | Apgar score <4 at 5 minutes | Apgar Score of 4 or less (of all deliveries over 1,500 grams) | Apgar score of 4 or less | Of all deliveries over 1,500 grams | (28) |
|  | Apgar score <5 at 5 minutes | Neonates with low Apgar scores at 5 minutes (below threshold) | Number of neonates with Apgar < 5 at 5 minutes | Total deliveries | (79) |
|  | Apgar score <7 at 5 minutes         Apgar score <7 at 5 minutes  *(continued)* | 5 min APGAR <7 | Number of pregnancies complicated by this measure | Total number of deliveries for that time period | (95) |
|  |  | Apgar score < 7 at 5 min | Number of live-born infants with Apgar score < 7 at 5 min | Total number of live-born infants (stillborn infants excluded) | (86) |
|  |  | Apgar score <7 five minutes post-birth | Births with Apgar <7 at 5 min | All births | (106) |
|  |  | Birth >=37wk with Apgar <7 at 5 min | Number of births >=37 wk with Apgar <7 at 5 min | Total number of births 27 wk | (87) |
|  |  | Births >=37 wk with Apgar score < 7 at 5 min | Number of live-born infants with a birth >=37 wk and an Apgar score < 7 at 5 min | Total number of live-born infant >=37 wk (stillborn infants excluded) | (86) |
|  |  | Low five-minute Apgar score (< 7) | Number of cases | Time period | (47) |
|  |  | Percentage of neonates with an Apgar score <7 at 5 min | Number of neonates with Apgar score <7 at 5 minutes | Total number of neonates | (59) |
|  |  | PI 04–Apgar score of less than 7 at 5 minutes for births at or after term | The number of live born babies at or after term (from 37 completed weeks gestational age) with an Apgar score of less than 7 at 5 minutes. | The number of live born babies born at or after term (from 37 completed weeks gestational age). | (102) |
|  |  | Rate of newborns with Apgar score <7 at 5 minutes | Number of infants with Apgar <7 | Total live births | (64) |
|  |  | Term babies with 5 min APGAR scores < 7 | Term babies with 5 min APGAR scores < 7 | Numbers of babies born, and women delivered | (68) |
|  |  | Term infants with Apgar score less than 7 at 5 min after birth | Term infants with Apgar score less than 7 at 5 min after birth | Infants | (30) |
|  | Apgar score at 5 minutes | Apgar scores distribution at 5 minutes post-birth | Births with Apgar scores by category | Total live births | (90) |
|  |  | Distribution of APGAR score at 5 minutes | Apgar score at 5 minutes (0-10) | Live births | (9) |
|  | Apgar score <9 at 5 minutes | Rate of neonates <32 weeks with low Apgar at 5 mins | Number of low Apgar scores at <32 weeks | Total births at <32 weeks | (75) |
|  |  | Rate of neonates ≥37 weeks with low Apgar at 5 mins | Number of low Apgar scores at ≥37 weeks | Total births at ≥37 weeks | (7) |
|  |  | Rate of neonates 32-36 weeks with low Apgar at 5 mins | Number of low Apgar scores at 32-36 weeks | Total births at 32-36 weeks | (75) |
| **NICU admission** | NICU admission | Admission to neonatal intensive care unit | Number of neonates admitted to NICU | Total number of live births | (20) |
|  |  | NICU admissions of neonates >2.5 kg without birth defects | Number of NICU admissions | Total number of neonates | (7) |
|  | NICU admission >2,500g > 24 hours | Neonates >2500g admitted to NICU >24 hours | NICU stays meeting criteria | Total births >2500g | (106) |
|  | NICU admission for >2500g without birth defects | Rate of non-low-birth-weight neonates admitted to the NICU | Number of NICU admission of neonates >2500g without birth defects | Total number of neonates (all live born neonates including those with birth defects) | (87) |
|  | NICU admission, term | Immediate transfers to any neonatal unit for infants born >=37wk | Number of infants born >=37 wk transferred to any neonatal unit | N/Total number of live-born infants >=37 wk (admissions for congenital malformations excluded) | (86) |
|  |  | NICU admissions at term ≥ 37 weeks | NICU admissions at term ≥ 37 weeks | Numbers of babies born, and women delivered | (68) |
|  |  | Term Infant Admitted to Neonatal ICU (of all deliveries on or after 37 weeks of gestation) | Term infants admitted to NICU | Of all deliveries on or after 37 weeks of gestation | (28) |
|  |  | Term neonates unexpectedly admitted to NICU following delivery | Number of term neonates admitted to NICU unexpectedly | Total term deliveries | (79) |
|  | SCBU admission | Proportion of newborns admitted to SCBU following delivery | Number of SCBU admissions | Total live births | (64) |
| **Newborn transfer** | Newborn transfer | Neonatal Transfer to Hospital with Neonatal ICU (of deliveries at hospital without neonatal ICU) | Neonates transferred to hospital with NICU | Of deliveries at hospital without neonatal ICU | (28) |
|  |  | Neonatal transfers to intensive care or neonatology unit | Number of live-born infants transferred to an intensive care or neonatology unit | Total number of live-born infants (admissions for congenital malformations and transfer to kangaroo care unit excluded) | (86) |
|  |  | Neonatal transfers to intensive care unit | Number of live-born infants transferred to intensive care unit | Total number of live-born infants (admissions for congenital malformations excluded) | (86) |
| **Seizures** | Term Infant Seizures | Term Infant Seizures (of all deliveries on or after 37 weeks of gestation) | Term Infant Seizures (of all deliveries on or after 37 weeks of gestation) | Of all deliveries on or after 37 weeks of gestation | (28) |
| **Unexpected Complications in Term Newborns** | Unexpected Complications in Term Newborns | Unexpected Complications in Term Newborns | PC-06.0 Newborns with severe complications and moderate complications.  PC-06.1 Newborns with severe complications.  PC-06.2 Newborns with moderate complications. | Liveborn single term newborns 2500 gm or over in birth weight. | (105) |
| **Infection, neonatal** | Neonatal Infection Rate | Rate of confirmed bloodstream infections in neonates during hospital stay | Number of newborns with microbiologically confirmed bloodstream infections | Total eligible newborns | (85) |
|  |  | Rate of neonatal infections among low-risk obstetrical patients | Number of neonates with documented infection (ICD-9-CM codes 770.0, | Total number of term, low-risk neonates delivered in the facility | (62) |
| **Prematurity, neonatal** | Preterm birth | Live-born infants before < 28 wk gestation | Number of live-born infants before < 28 wk gestation | Total number of live-born infants | (86) |
|  |  | Live-born infants before < 32 wk gestation | Number of live-born infants before < 32 wk gestation | Total number of live-born infants |  |
|  |  | Live-born infants before < 34 wk gestation | Number of live-born infants before < 34 wk gestation | Total number of live-born infants |  |
|  |  | Live-born preterm neonate | Number of live-born infants before < 37 wk gestation | Total number of live-born infants |  |
|  |  | Premature births (less than 37 weeks gestation) | Number of premature live births (gestational age between 24-36 weeks) and all stillbirths | Number of total live births and stillbirths | (100) |
|  |  | Preterm birth | Total number of babies born under 37 weeks’ gestation | Total number of babies born (live births) | (104) |
|  |  | Proportion of preterm births | Number of live births in Canada with gestational age at birth of less than 37 weeks | Hospital live births in Canada. Excludes live births with unknown gestational age. | (103) |
| **Hospitalization** | Admission to paediatrics department within a week after birth | Infants admission to paediatrics department within a week after birth | Infants admission to paediatrics department within a week after birth | Infants without antenatally congenital anomalies | (30) |
| **Readmission rate** | Readmission rate | PS19 Unplanned readmission rate within 3 days after discharge | Number of children aged ≤18 who readmitted within 3 days after discharge | Number of children aged ≤18 | (50) |

***Indicator constructs:** Umbrella concepts aggregating multiple related indicator groups. **^$^Standardised indicator labels:** Primary analytical units clustering individual indicators with closely related definitions.
