# Supplementary material for: Monitoring childbirth and immediate postpartum care quality: A scoping review of indicators in high-income countries
Source: PLoS One. 2026 Sep 15;21(9):e0350665. doi: 10.1371/journal.pone.0350665 (PMC13577461; doi:10.1371/journal.pone.0350665)
Supplement: Supplementary file 8 [file pone.0350665.s009.docx]

**S6 Table: List of maternal and neonatal outcome indicators (N=3).**

| **Indicator Constructs* Maternal and Neonatal** | **Standardised Indicators^$^** | **Name/ definition of indicator *per source*** | **Numerator *per source*** | **Denominator *per source*** | **References** |
| --- | --- | --- | --- | --- | --- |
| **Composite indicator** | Adverse Outcome Index (AOI) | A composite of 10 maternal and newborn adverse events related to labour and delivery: maternal death, intrapartum or neonatal death, uterine rupture, maternal admission to the ICU, birth trauma, unanticipated operative procedures, NICU admission >24h, 5-min Apgar <7, blood transfusion, and 3rd or 4th degree perineal tear. | The number of deliveries with one or more of the 10 specified adverse outcomes (e.g., maternal death, uterine rupture, NICU admission >24 hours). | The total number of deliveries in the study population. | (58) |
|  | Severity Index (SI) | The SI measures the average severity of adverse events for cases with at least one adverse outcome. It's calculated by dividing total severity points by the number of deliveries with an adverse outcome. | Total severity points across all adverse outcomes | Total number of deliveries with at least one adverse event | (58) |
|  | Weighted Adverse Outcome Score (WAOS) | The WAOS weights each AOI component by severity, combining the frequency and severity of events. It’s calculated by assigning points to each adverse event based on severity, summing these, and dividing by the total number of deliveries | Total severity points across all adverse outcomes | Total number of deliveries | (58) |
