# Supplementary material for: Monitoring childbirth and immediate postpartum care quality: A scoping review of indicators in high-income countries
Source: PLoS One. 2026 Sep 15;21(9):e0350665. doi: 10.1371/journal.pone.0350665 (PMC13577461; doi:10.1371/journal.pone.0350665)
Supplement: Supplementary file 9 [file pone.0350665.s010.docx]

**S7 Table. List of complete maternal process indicators (N = 210).**

| **Indicator Constructs***  **maternal** | **Standardised Indicators^$^** | **Name/ definition of indicator *per source*** | **Numerator *per source*** | **Denominator *per source*** | **References** |
| --- | --- | --- | --- | --- | --- |
| **Onset of labour** | Elective delivery | Elective (non-medically indicated) deliveries at >= 37 and < 39 weeks of gestation | All deliveries that were associated with medical inductions of labor or cesarean delivery at >= 37 and < 39 weeks of gestation | Per 100 deliveries ≥37 and < 39 weeks gestation. | (35) |
|  |  | Elective Early Term Delivery performed between 37 and 39 weeks of gestation without medical necessity. | Number of elective deliveries at 37-39 weeks | Total eligible deliveries between 37-39 weeks | (85) |
|  |  | Elective deliveries | Patients with elective deliveries | Patients delivering newborns with >= 37 and < 39 weeks of gestation completed | (105) |
|  |  | Percentage of elective deliveries performed before 39 weeks of gestation without medical indication | Number of elective deliveries <39 weeks without indication | Total scheduled deliveries <39 weeks | (65) |
|  | Induced labour OR elective caesarean | Percentage of women with induced labor or undergoing elective cesarean section | Women with induced labor or undergoing elective cesarean section | All women giving birth | (37) |
|  | Induction of labour | Induction of labor among women who underwent a trial of labor | Women who had induction of labor | Women who had spontaneous labor | (92) |
|  |  | Inductions of labor | Number of inductions of labor | Total number of deliveries | (86) |
|  |  | Induction of labour for selected females giving birth for the first time | The number of selected females who gave birth for the first time and who had labour induced. | All selected females. | (102) |
|  |  | Proportion of women induced with an indication of post-dates who are at less than 41 weeks’ gestation at delivery | The number of women who were at less than 41 weeks of gestation at delivery | The total number of women who had labour induction with an indication for induction of “post-dates pregnancy” (in a given place and time). | (72) |
|  |  | Rate of labour inductions | Number of deliveries with induced labour | Total number of deliveries | (39) |
|  |  | Standard primiparae who undergo induction of labour | Total number of standard primiparae who undergo induction of labour | Total number of standard primiparae | (104) |
|  |  | Women with a term, singleton infant in vertex position delivered by labour induction | Women with a term, singleton infant in vertex position delivered by labour induction | Pregnant women | (30) |
|  |  | Women with induced labour | Women with induced labour | Numbers of babies born, and women delivered | (68) |
|  | Births, onset of labour | Number of deliveries associated with each of the following modes of labour onset, as a proportion of deliveries resulting in a live or stillbirth:  -Spontaneous onset  -Caesarean section prior to the onset of labour  -Labour induction (initiation of uterine contractions) by medical or surgical means prior to the onset of labour. | Number of deliveries associated with each of the following modes of labour onset:  -Spontaneous onset  -Caesarean section prior to the onset of labour  -Labour induction (initiation of uterine contractions) by medical or surgical means prior to the onset of labour. | Deliveries resulting in a live or stillbirth  For all births and by gestational age, using the following groups: 22-27, 28-31, 32-36, 37, 38, 39, 40, 41, 42+ | (9) |
| **Onset of labour**  *(continued)* | Births, onset of labour *(continued)* | Number of deliveries associated with each of the following modes of labour onset, as a proportion of deliveries resulting in a live or stillbirth:  -Spontaneous onset  -Caesarean section prior to the onset of labour  -Labour induction (initiation of uterine contractions) by medical or surgical means prior to the onset of labour. | The number of deliveries associated with each of the following modes of labour onset, as a proportion of deliveries resulting in a live or stillbirth:  - Spontaneous onset  -Caesarean section prior to the onset of labour-Labour induction (initiation of uterine contractions) by medical or surgical means prior to the onset of labour. | For all births and by gestational age, using the following groups: 22-27, 28-31, 32-36, 37, 38, 39, 40, 41, 42+ | (81) |
| **Labour, management** | Assessment during first stage labour | Assessment during first stage labour: (1) 8-hourly temperature and blood pressure, (2) half hourly‑ frequency of contractions and foetal heart rate, (3) vaginal examination 4-hourly or if there is concern about progress or in response to the woman’s wishes, (4) woman’s emotional and psychological needs, including her desire for pain relief | Assessment during first stage labour | Pregnant women admitted during first labour | (30) |
|  | Assessment during second stage labour | Assessment during second stage labour: (1) 1-hourly blood pressure and woman’s heart rate, (2) half-hourly frequency of contractions, (3) half-hourly foetal heart rate, (4) frequency of passing urine, (5) vaginal examination 1-hourly or if there is concern about progress or in response to the woman's wishes, (6) woman’s emotional and psychological needs | Assessment during second stage labour | Pregnant women admitted for delivery at hospital | (30) |
|  | Augmentation of labour | Rate of labor augmentation with Syntocinon | Number of Syntocinon administrations | Total standard primigravidae | (64) |
|  | Comfortable positions throughout second stage labour | Women having been encouraged and supported to adopt the most comfortable positions throughout second stage labour | Comfortable positions throughout second stage labour | Women confirmed the safety for baby in comfortable positions | (30) |
|  | Examination of uterus | Frequency of manual uterine exams post-birth | Number of women undergoing uterine exam | Total number of women | (75) |
|  | Initial assessment at admission | Initial assessment of labour risk at admission: (1) measuring foetal heart rate more than 20 min, (2) vaginal examination, (3) frequency of construction, (4) woman’s emotional and psychological needs, (5) a part and level of pain including her desire for pain relief, (6) foetal movement | Initial assessment of labour risk at admission | Pregnant women admitted for delivery at hospital | (30) |
|  | Prolonged second stage | Prolonged second stage | Women with a prolonged second stage | Women who reached the second stage but had a normal second-stage length | (107) |
| **Labour, management**  *(continued)* | Diagnostics | Proportion of labor cases with fetal blood sampling | Number of fetal blood sampling cases | Total standard primigravidae | (64) |
|  |  | Unplanned CS: Application of additional diagnostics such as ST-analysis or fetal scalp blood sampling to all women with suspected fetal distress, if technically possible and no contraindications to the procedure | Number of women to whom the indicator applied and actual care was consistent with the indicator | Total number of women to whom the indicator applied | (48) |
|  | Non-progressive labour first stage | Unplanned CS: adequate contractions in case of non-progressive labor first stage | Number of women to whom the indicator applied and actual care was consistent with the indicator | Total number of women to whom the indicator applied |  |
|  |  | Unplanned CS: in case of non-progressive labor first stage, optimal situation (5 previous indicators)>2hrs before performing a CS | Number of women to whom the indicator applied and actual care was consistent with the indicator | Total number of women to whom the indicator applied |  |
|  |  | Unplanned CS: in case of non-progressive labor first stage, optimal situation (5 previous indicators)>4hrs before performing a CS | Number of women to whom the indicator applied and actual care was consistent with the indicator | Total number of women to whom the indicator applied | (48) |
|  |  | Unplanned CS: rupture of membranes in case of non-progressive labor first stage | Number of women to whom the indicator applied and actual care was consistent with the indicator | Total number of women to whom the indicator applied | (48) |
|  | Non-progressive labour first stage  *(continued)* | Unplanned CS: urinary catheterization in case of non-progressive labor first stage | Number of women to whom the indicator applied and actual care was consistent with the indicator | Total number of women to whom the indicator applied | (48) |
|  |  | Unplanned CS: use of epidural analgesia in case of non-progressive labor first stage | Number of women to whom the indicator applied and actual care was consistent with the indicator | Total number of women to whom the indicator applied | (48) |
|  |  | Unplanned CS: use of pain medication in case of non-progressive labor first stage | Number of women to whom the indicator applied and actual care was consistent with the indicator | Total number of women to whom the indicator applied |  |
|  | Non-progressive labour second stage | Unplanned CS: in case of non-progressive labor second stage in nulliparous women, active pushing recommended | Number of women to whom the indicator applied and actual care was consistent with the indicator | Total number of women to whom the indicator applied |  |
|  |  | Unplanned CS: in case of non-progressive labor second stage in nulliparous women, adequate contractions recommended | Number of women to whom the indicator applied and actual care was consistent with the indicator | Total number of women to whom the indicator applied |  |
|  |  | Unplanned CS: in case of non-progressive labor second stage in nulliparous women, consider vaccum extraction if the head is <1/5th palpable per abdomen | Number of women to whom the indicator applied and actual care was consistent with the indicator | Total number of women to whom the indicator applied |  |
| **Anaesthesia/pain relief** | Epidural analgesia | Epidural analegia use | Number of women with epidural analgesia use | Total number of women delivered | (87) |
|  |  | Epidural analgesia for vaginal delivery | Number of women receiving epidural analgesia for vaginal delivery | Total number of vaginal deliveries | (86) |
|  | Rate of epidural availability | Rate of epidural availability | Number of women receiving epidural analgesia | Total number of women | (75) |
|  | Rate of spinal/epidural within 1 hour of prescription | Proportion of birth epidural/birth spinal given within one hour from prescription. Standard: At least 95% | Birth epidural and birth spinal given within one hour | All birth epidurals and birth spinals given and requested for women with intended vaginal delivery | (71) |
| **Anaesthesia/pain relief**  *(continued)* | Spinal analgesia for caesareans | Spinal analgesia for cesareans | Number of women receiving spinal analgesia for cesareans | Total number of cesarean deliveries | (86) |
| **Management, prematurity** | Antibacterial drugs for premature rupture of membrane | Antibacterial drugs for premature rupture of membrane | Number of mothers with preterm prelabour rupture of membranes and offered antibacterial drugs | Number of mothers with preterm prelabour rupture of membranes | (50) |
|  | Avoiding combining different tocolytics | Avoiding combining different tocolytics | Number of mothers with pregnancy or give birth and avoided combining different tocolytics | Number of mothers with pregnancy or give birth | (50) |
|  | Avoiding tocolysis in patients with PPROM | Avoiding tocolysis in patients with PPROM | Number of mothers with preterm prelabour rupture of membranes (gestational week at admission < 35) who avoided tocolysis treatment | Number of mothers with preterm prelabour rupture of membranes (gestational week at admission < 35) | (50) |
|  | Avoiding tocolytic maintenance therapy | Avoiding tocolytic maintenance therapy | Number of mothers with preterm labour or threatened preterm labour who offered tocolytic treatment not more than 24 hours. | Number of mothers with preterm labour or threatened preterm labour who offered tocolytic treatment | (50) |
|  | Corticosteroids administration  Corticosteroids administration  *(continued)* | Corticosteroids administration for threatened preterm labour | Number of mothers with threatened preterm labour and give birth 24-33 gestational week (without ruptured membranes), and offered prenatal corticosteroids | Number of mothers with threatened preterm labour and give birth 24-33 gestational week (without ruptured membranes) | (50) |
|  |  | Corticosteroids administration for preterm labour with premature rupture of membrane | Number of mothers with threatened preterm labour, ruptured membranes, and give birth 24-33 gestational week, and offered prenatal corticosteroids | Number of mothers with threatened preterm labour, ruptured membranes, and give birth 24-33 gestational week | (50) |
| **Delivery, vaginal** | Births without obstetric intervention | Births without obstetric intervention | Number of deliveries without selected obstetric interventions | All deliveries resulting in a live birth or stillbirth. | (9) |
|  | Vaginal birth after caesarean (VBAC) | All vaginal births after cesarean delivery | All vaginal births after cesarean delivery per 1000 women with previous cesarean delivery | Previous cesarean | (80) |
|  |  | Attempted VBAC (of all deliveries with prior CSEC) | Attempted VBAC | Of all deliveries with prior CSEC | (28) |
|  |  | Females having their second birth vaginally whose first birth was by caesarean section | The number of females having their second birth vaginally whose first birth was by caesarean section. | The number of females having their second birth whose first birth was by caesarean section. | (102) |
|  |  | Uncomplicated vaginal birth after cesarean delivery (VBAC) | Uncomplicated vaginal birth after cesarean delivery (VBAC) per 1000 women with previous cesarean delivery | Previous cesarean | (80) |
|  |  | Vaginal birth after cesarean rate: 1) VBAC rate = #VBAC/ # women with prior cesarean; 2) Attempted VBAC rate = # women with prior cesarean who attempt VBAC/# women with prior cesarean; and 3) Successful VBAC = # VBAC/ # women with prior cesarean who attempt VBAC | 1) VBAC rate = #VBAC/ ; | 1) VBAC rate = / # women with prior cesarean; | (60) |
|  |  |  | 2) Attempted VBAC rate = # women with prior cesarean who attempt VBAC/; and | 2) Attempted VBAC rate = /# women with prior cesarean; and |  |
|  |  |  | 3) successful vbac = # vbac/ | 3) Successful VBAC = / # women with prior cesarean who attempt VBAC |  |
| **Delivery, vaginal**  *(continued)* | Vaginal delivery | Spontaneous vaginal deliveries (non-instrumental) | Number of non-instrumental spontaneous vaginal deliveries | Total number of vaginal deliveries | (86) |
|  |  | Successl Vaginal Delivery without Prior Cesarean (VBAC) (of all deliveries with prior CSEC, of all attempted VBAC) | Successl Vaginal Delivery without Prior Cesarean | Of all deliveries with prior CSEC, of all attempted VBAC | (28) |
|  |  | Vaginal deliveries | Number of vaginal deliveries | Total number of deliveries | (86) |
|  |  | Women with a term, singleton infant in vertex position delivered by vaginal delivery | Women with a term, singleton infant in vertex position delivered by vaginal delivery | Pregnant women | (30) |
|  | Vaginal delivery in MLU | Women planning spontaneous vaginal birth in a midwifery ward, and being able to follow that plan | Spontaneous vaginal birth in a midwifery unit | Women planning childbirth at midwifery unit in hospital during second trimester | (30) |
|  | Vaginal delivery, nulliparous | Non-instrumental vaginal birth for selected females giving birth for the first time | The number of selected females giving birth for the first time who gave birth by non-instrumental vaginal birth. | All selected females. | (102) |
|  |  | Proportion of standard primigravidae with spontaneous vaginal delivery | Number of spontaneous vaginal deliveries | Total standard primigravidae | (64) |
|  |  | Standard primiparae who have a spontaneous vaginal birth | Total number of standard primiparae who have a spontaneous vaginal birth at a maternity facility | Total number of standard primiparae | (104) |
| **Delivery, instrumental** | Instrument delivery | Forceps/Vacuum-Assisted Delivery (of all deliveries with trial of labor) | Forceps/vacuum-assisted delivery | Of all deliveries with trial of labor | (28) |
|  |  | Instrumental vaginal deliveries | Number of instrumental vaginal deliveries | Total number of vaginal deliveries | (86) |
|  |  | Instrumental vaginal delivery | Number of neonates delivered by instrumental extraction (using obstetric forceps or vacuum extractor) | Total number of vaginally births (all live born neonates including those with birth defects) | (87) |
|  |  | Instrumental vaginal delivery rate | Instrumental vaginal delivery rate | Numbers of babies born, and women delivered | (68) |
|  |  | Instrumental vaginal birth for selected females giving birth for the first time | The number of selected females giving birth for the first time who gave birth by instrumental vaginal birth. | All selected females. | (102) |
|  |  | Rate of births with instrumental assistance | Number of instrumental extractions | Total number of neonates | (75) |
|  |  | Standard primiparae who undergo an instrumental vaginal birth | Total number of standard primiparae who undergo an instrumental vaginal birth | Total number of standard primiparae | (104) |
|  |  | Women with a term, singleton infant in vertex position delivered by instrument delivery | Women with a term, singleton infant in vertex position delivered by instrument delivery | Pregnant women | (30) |
|  | Assisted Vacuum Deliveries | Frequency of assisted vacuum deliveries during labor | Number of assisted vacuum deliveries | Total deliveries | (79) |
| **Births** | Mode of delivery | Breakdown of delivery methods (e.g., vaginal, C-section) by parity and fetal presentation | Deliveries by mode (parity, presentation) | Total births by delivery type | (90) |
| **Births**  *(continued)* | Mode of delivery  *(continued)* | For a population of women and year: the number of births associated with each of the following modes:  Vaginal, non instrumental  Vaginal: forceps  Vaginal: ventouse  Vaginal (other, Thierry’s spatula, etc..)  Caesarean: before or at onset of labour/elective  Caesarean: during labour/emergency   As a proportion of all still and live births. Computed by parity, plurality, presentation and previous caesarean section and gestational age  (22-27, 28-31, 32-36, 37, 38, 39, 40, 41, 42+) | Number of births associated with each of  the following modes:- Vaginal, non instrumental- Vaginal: forceps- Vaginal: ventouse- Vaginal (other, Thierry’s spatula, etc..)- Caesarean: before or at onset of labour/elective- Caesarean: during labour/emergency | All still and live births | (81) |
|  |  | For a population of women and year: the number of births associated with each of  the following modes:- Vaginal, non instrumental- Vaginal: forceps- Vaginal: ventouse- Vaginal (other, Thierry’s spatula, etc..)- Caesarean: before or at onset of labour/elective- Caesarean: during labour/emergency as a proportion of all still and live births. | For a population of women and year: the number of births associated with each of  the following modes:- Vaginal, non instrumental- Vaginal: forceps- Vaginal: ventouse- Vaginal (other, Thierry’s spatula, etc..)- Caesarean: before or at onset of labour/elective- Caesarean: during labour/emergency | All still and live births | (9) |
| **Episiotomy** | Episiotomy | Episiotomies | Number of women with an episiotomy for vaginal delivery | Total number of vaginal deliveries | (86) |
|  |  | Episiotomy rate among all women | Episiotomy rate among all women | All women | (76) |
|  |  | Episiotomy rate among women with a spontaneous vaginal delivery | Episiotomy rate among women with a spontaneous vaginal delivery | Women with a spontaneous vaginal delivery | (31) |
|  |  | Frequency of episiotomy in vaginal births | Births with episiotomy | Total vaginal births | (84) |
|  |  | Percentage of Women with an Episiotomy | Number of episiotomies performed | Total number of vaginal births | (59) |
|  |  | Episiotomy rate | The number of women who had an episiotomy | All women who had a vaginal delivery (divided by instrumental and non-instrumental deliveries) | (9) |
|  |  | Rate of episiotomy in spontaneous vaginal births | The number of women who had spontaneous vaginal births with episiotomy | The total number of women who had spontaneous vaginal births at a given hospital | (72) |
|  |  | Rate of all episiotomies for both nulliparous and multiparous women who delivered vaginally and did not have shoulder dystocia | Episiotomy rate for nulliparous women | Total number of birth | (65) |
| **Episiotomy**  *(continued)* | Episiotomy, primiparous | Episiotomies in primiparas | Number of primiparas with an episiotomy in vaginal delivery | Total number of vaginal deliveries in primiparas | (86) |
|  |  | Incidence of an episiotomy for primiparous mothers in public accommodation aged between 20-34 with a SVD of a singleton, term infant | The number of primiparous mothers in public accommodation aged between 20-34 with an SVD of a singleton, term infant with an episiotomy. | The number of primiparous mothers in public accommodation aged between 20-34 with an SVD of a singleton, term infant. | (51) |
|  |  | Episiotomy for females giving birth for the first time and giving birth vaginally | (a) The number of females having their first baby who had an episiotomy during a non-instrumental vaginal birth. | (a) The number of females having their first baby who had a non-instrumental vaginal birth. | (102) |
|  |  |  | (b) The number of females having their first baby who had an episiotomy during an instrumental vaginal birth. | (b) The number of females having their first baby who had an instrumental vaginal birth. |  |
|  |  | Proportion of women undergoing episiotomy during delivery | Number of episiotomies | Total standard primigravidae | (64) |
|  |  | Standard primiparae undergoing episiotomy and no third- or fourth-degree perineal tear | Total number of standard primiparae undergoing episiotomy and no third- or fourth-degree perineal tear with vaginal birth | Total number of standard primiparae who give birth vaginally | (104) |
|  | Episiotomy with third and/or fourth degree perineal tears | Standard primiparae undergoing episiotomy and sustaining a third- or fourth-degree perineal tear | Total number of standard primiparae undergoing episiotomy and sustaining a third- or fourth-degree perineal tear with vaginal birth | Total number of standard primiparae who give birth vaginally | (104) |
|  | Episiotomy, multiparous | Incidence of an episiotomy for multiparous mothers in public accommodation aged between 20-34 with a SVD of a singleton, term infant | The number of multiparous mothers in public accommodation aged between 20-34 with an SVD of a singleton, term infant with an episiotomy. | The number of multiparous mothers in public accommodation aged between 20-34 with an SVD of a singleton, term infant. | (51) |
| **Caesarean section** | Caesarean section rate | An adjusted measurement of the rate of cesarean deliveries that accounts for individual patient risk factors, enabling fair comparison of cesarean rates across hospitals. | The difference between the observed number of cesarean events and the expected number of cesarean events (Observed−Expected). | Total number of deliveries. | (55) |
|  |  | Caesarean section % | Total number of deliveries where the episode type is '2' (delivery episode) or '5' (other delivery event), and where the actual place of delivery is not '1' (at a domestic address), '5' (in a private hospital) or '6' (in another hospital or institution) or the actual place of delivery is null with primary procedure (3 character) R17 or R18. In 2012/13, R25.1 (caesarean hysterectomy) was included. | Total number of episodes where the episode type is '2' (delivery episode) or '5' (other delivery event), and where the actual place of delivery is not '1' (at a domestic address), '5' (in a private hospital) or '6' (in another hospital or institution) or the actual place of delivery is null | (100) |
|  |  | Cesarean delivery | Cesarean delivery per 1000 deliveries | All deliveries | (80) |
|  |  | Cesarean delivery rate (cd rate) | Number of cesarean deliveries | Total deliveries | (106) |
|  |  | Cesarean Delivery Rate (CSEC) (% of deliveries w/o prior CSEC) | Cesarean delivery rate | Of all deliveries, of deliveries w/o prior CSEC | (28) |
|  |  | Cesarean delivery rates adjusted for maternal, fetal, and labor risk factors to ensure fair comparison across hospitals | Number of cesarean deliveries performed in the hospital | Total number of eligible deliveries in the hospital population | (88) |
|  |  | Cesarean rate among all women | Cesarean rate among all women | All women | (76) |
| **Caesarean section**  *(continued)* | Caesarean section rate *(continued)* | Cesarean rate: Primary (# cesarean / # women delivering without prior cesarean) and repeat (# cesarean/# women delivering with history of prior cesarean) | Primary (# cesarean) | Primary ( # women delivering without prior cesarean) | (60) |
|  |  |  | Repeat :# cesarean | Repeat (# women delivering with history of prior cesarean) |  |
|  |  | Cesarean section rate | Performance measurement system A : Cesarean sections, DRG 370, 371 | Deliveries, DRG 370–375 | (44) |
|  |  |  | Performance measurement system B  Cesarean sections, Procedure codes: 74.0, 74.1, 74.2, 74.4, 74.99 | Deliveries, Procedure codes: 72.0, 72.1, 72.21, 72.29, 72.31, 72.39, 72.4, 72.51, 72.53, 72.54, 72.6, 72.71, 72.79, 72.8, 72.9; 73.22, 73.51, 73.59, 73.6; 74.0, 74.1, 74.4, 74.99 | (44) |
|  |  | Cesarean section rate *(continued)* | Performance measurement system C: Cesarean sections, Procedure codes: 74.0, 74.1, 74.2, 74.4, 74.99 | Deliveries, Diagnosis codes: V27.0–V27.9 | (44) |
|  |  |  | Performance measurement system D: Cesarean sections, Procedure codes: 74.0, 74.1, 74.2, 74.4, 74.99 | Deliveries, Diagnosis codes: 640.81–669.92 or V27.0–V27.9 | (44) |
|  |  | Number of unplanned CS events in total births | Number of unscheduled CS | Total number of women | (75) |
|  |  | Primary cesarean | Primary cesarean per 1000 deliveries | All deliveries | (80) |
|  |  | Proportion of cesarean deliveries | Number of cesarean deliveries (primary cesarean, repeat cesarean, or either) in Canada | Total number of hospital deliveries for women (those without previous cesarean, those with a previous cesarean, or either) in Canada | (103) |
|  |  | Rate of Caesarean section deliveries | Number of CS deliveries | Total number of deliveries | (39) |
|  |  | Total Cesarean deliveries (% of all deliveries) | Total Cesarean deliveries | All deliveries | (28) |
|  |  | Women with a term, singleton infant in vertex position delivered by caesarean section | Women with a term, singleton infant in vertex position delivered by caesarean section | Pregnant women | (30) |
|  | Caesarean section under general anaesthesia (GA) | General anesthesia for cesarean delivery | Number of women receiving general anesthesia for cesareans | Total number of cesarean deliveries | (86) |
|  |  | People having a general anaesthetic for caesarean section | Total number of people having a general anaesthetic for caesarean section | Total number of people who undergo caesarean section | (104) |
|  |  | General anaesthetic for females giving birth by caesarean section | The number of females who gave birth by caesarean section and had a general anaesthetic. | Number of females who gave birth by caesarean section and recorded a permissible value for general anaesthetic. | (102) |
|  |  | Rate of cesarean deliveries under general anesthesia during labor | Rate of cesarean deliveries under general anesthesia during labor | All women | (76) |
|  | Caesarean section, planned | Caesarean section before labour | Number of caesarean sections before labour | Total number of women delivered | (87) |
|  |  | Cesareans before labor | Number of cesarean deliveries before labor | Total number of cesarean deliveries | (86) |
|  |  | Number of planned CS events in total births | Number of scheduled CS | Total number of women | (75) |
|  |  | Planned cesarean deliveries | Number of planned cesarean deliveries performed on or before the scheduled due date (surgical indication recorded 48h before performance) | Total number of cesarean deliveries | (86) |
|  |  | Planned cesareans 39 wk | Number of planned cesareans 39 wk | Total number of cesarean deliveries | (86) |
| **Caesarean section**  *(continued)* | Caesarean section, Robson | Cesarean sections according to Robson 10-group classification | Number of cesareans in:  1. Nulliparas, singleton cephalic fetus, 37 wk, in spontaneous labor  2. Nulliparas, singleton cephalic fetus 37 wk, induction or cesarean before labor  3. Multiparas (excluding previous cesarean), singleton cephalic fetus, 37 wk, in spontaneous labor  4. Multiparas (excluding previous cesarean), singleton cephalic fetus, 37 wk, induction or cesarean before labor  5. Previous cesarean, singleton cephalic fetus, 37 wk  6. All nulliparas with fetus in breech presentation  7. All multiparas with fetus in breech presentation (including previous cesarean)  8. All multiple pregnancies (including previous cesarean)  9. All abnormal lies (including previous cesarean)  10. All singleton cephalic fetuses, 36 wk (including previous cesarean) | Total number of deliveries 2 according to the description of each group (for example, for group 1: total number of deliveries in nulliparas, with a singleton cephalic fetus, 37 wk, in spontaneous labor) | (86) |
|  |  |  |  |  |  |
|  |  | CSR: Total, Robson groups 1, 2 and 5 | CSR: Total, Robson groups 1, 2 and 5 | Numbers of babies born, and women delivered | (68) |
|  | Obstetric trauma during caesarean section | Cesarean delivery | Number of trauma cases recorded per 1000 cesarean deliveries | Total number of cesarean deliveries in a given population or hospital | (33) |
|  | Caesarean section, multiples | A primary cesarean section performed without attempting vaginal delivery. | Number of twin pregnancies with planned CS. | Total number of twin pregnancies | (40) |
|  |  | A secondary cesarean section performed during labor after an initial plan for vaginal delivery. | Number of twin pregnancies with intrapartum CS. | Total number of twin pregnancies | (40) |
|  |  | Cesareans in non-singleton pregnancy | Number of cesareans in non-singleton pregnancy | Total number of non-singleton deliveries | (86) |
|  | Caesarean section, indicated | Cesarean Delivery for Failure of Labor to Progress (of all deliveries without prior CSEC, of all primary CSEC deliveries) | Cesarean Delivery for Failure of Labor to Progress | Of all deliveries without prior CSEC, of all primary CSEC deliveries | (28) |
|  |  | Caesarean Section for preterm birth and unstable lie | Number of mothers with preterm labour and unstable lie who underwent Caesarean Section | Number of mothers with preterm labour and unstable lie | (50) |
|  |  | Caesarean Section for placenta previa | Number of mothers with placenta praevia and offered selective Caesarean section within 2 days after from hospitalization by their gestational age week at admission < 39 | Number of mothers with placenta praevia and offered selective Caesarean section within 2 days after from hospitalization | (50) |
| **Caesarean section**  *(continued)* | Caesarean section, indicated  *(continued)* | Planned Caesarean Section for vasa previa | Number of mothers with vasa previa and offered selective Caesarean section within 2 days after from hospitalization by their 34-36 gestational age week at admission | Number of mothers with vasa previa and offered selective Caesarean section within 2 days after from hospitalization | (50) |
|  |  | Global cesareans 37 wk with a singleton pregnancy in breech presentation | Number of cesareans 37 wk with a singleton pregnancy in breech presentation | Total number of deliveries | (86) |
|  |  | Unplanned cesareans after failure of induction | Number of unplanned cesareans after induction of labor | Total number of inductions of labor | (86) |
|  | Caesarean section rate, low-risk | Caesarean section during labour in low-risk woman | Number of caesarean sections during labour in low-risk women | Women aged between 18 and 40 years with a singleton pregnancy, cephalic presentation, and no underlying co-morbidities during the pregnancy (e.g. Diabetes or hypertension, uterine scarring, admission before 37 weeks' gestational age…), no aspirin use during pregnancy, and no non-routine investigations such as foetal imaging by magnetic resonance imaging or computed tomography | (87) |
|  |  | Cesarean delivery performed in low-risk mothers | Cesarean deliveries among nulliparous patients with singleton vertex deliveries of newborns at ≥37 weeks gestation | Per 100 low-risk deliveries to nulliparous women with a term, singleton baby in a vertex position. | (35) |
|  |  | Cesarean rate among women at low risk of cesarean (singleton in cephalic presentation at term, no abnormal placental insertion, no history of cesarean, and no preeclampsia) | Cesarean rate among women at low risk of cesarean (singleton in cephalic presentation at term, no abnormal placental insertion, no history of cesarean, and no preeclampsia) | Women at low risk of cesarean (singleton in cephalic presentation at term, no abnormal placental insertion, no history of cesarean, and no preeclampsia) | (76) |
|  |  | Cesareans in women at low risk | Number of cesareans in women at low risk | Total number of deliveries in women at low risk | (86) |
|  |  | Rate of cesarean deliveries among low-risk, first-birth women, 37+ weeks with a singleton, vertex presentation | Number of cesarean births in low-risk, first-birth women | Total births for low-risk, first-birth women | (78) |
|  |  | Rate of C-section among low-risk births | Low-risk births with C-section | Total low-risk births | (84) |
|  |  | Rate of unplanned CS events among low-risk women | Number of unscheduled CS in low-risk women | Total low-risk women | (75) |
|  | Caesarean section, emergency | Proportion of birthing women giving birth with cesarean section degree 1 <15 minutes from decision to delivery Standard: At least 95% | Cesarean section, grade 1 with delivery within 15 minutes | All ordinated grade 1 cesarean sections | (71) |
|  |  | Proportion of birthing women giving birth with cesarean section degree 2 <30 minutes from decision to delivery. Standard: At least 95% | Cesarean section, grade 2 with delivery within 30 minutes | All ordinated grade 2 cesarean sections | (71) |
|  | Caesarean section, planned, repeat | Planned Caesarean Section for gestation 38 weeks and more | Number of mothers with previous cesarean delivery and offered selective Caesarean section within 2 days after from hospitalization after 38+ gestational week | Number of mothers with previous cesarean delivery and offered selective Caesarean section within 2 days after from hospitalization | (50) |
| **Caesarean section**  *(continued)* | Caesarean section rate, repeat | Cesareans after one previous cesarean | Number of cesareans after only one previous cesarean delivery | Total number of deliveries with only one previous cesarean delivery | (86) |
|  |  | Rate of repeat Caesarean section in low-risk women* not in labour at term, with no medical or obstetrical complications, prior to 39 weeks’ gestation | The number of women with a Caesarean section performed before 39 weeks’ gestation | The total number of low-risk women who had a repeat Caesarean section at term (in a given place and time). | (72) |
|  | Caesarean section, planned, low risk | Caesarean section before labour in low-risk woman | Number of caesarean sections before labour in low-risk women | Women aged between 18 and 40 years with a singleton pregnancy, cephalic presentation, and no underlying co-morbidities during the pregnancy (e.g. Diabetes or hypertension, uterine scarring, admission before 37 weeks' gestational age…), no aspirin use during pregnancy, and no non-routine investigations such as foetal imaging by magnetic resonance imaging or computed tomography | (87) |
|  |  | Cesarean rate among women at low risk of cesarean (singleton in cephalic presentation at term, no abnormal placental insertion, no history of cesarean, and no preeclampsia) and with a cesarean before labor | Cesarean rate among women at low risk of cesarean (singleton in cephalic presentation at term, no abnormal placental insertion, no history of cesarean, and no preeclampsia) and with a cesarean before labor | Women at low risk of cesarean (singleton in cephalic presentation at term, no abnormal placental insertion, no history of cesarean, and no preeclampsia) and with a cesarean before labor | (76) |
|  |  | Number of planned CS events among low-risk women | Number of scheduled CS in low-risk women | Total low-risk women | (75) |
|  | Caesarean section rate, during labour | Caesarean section during labour | Number of caesarean sections during labour | Total number of women delivered | (87) |
|  |  | Cesareans during labor | Number of cesareans during labor (among planned cesareans or planned vaginal deliveries) | Total number of cesarean deliveries | (86) |
|  | Caesarean section rate, nulliparous | Cesareans 37 wk for nulliparas with a singleton pregnancy in cephalic presentation | Number of cesareans 37 wkfor nulliparas with a singleton pregnancy in cephalic presentation | Total number of deliveries 37 wk for nulliparas with a singleton pregnancy in cephalic presentation | (86) |
|  |  | Cesareans among nulliparas | Number of cesareans among nulliparas | Total number of deliveries for nulliparas | (86) |
|  |  | Cesarean birth | Patients with cesarean births | Nulliparous patients delivered of a live term singleton newborn in vertex presentation | (105) |
|  | Caesarean section rate, nulliparous | Caesarean section for selected females giving birth for the first time | The number of selected females giving birth for the first time who gave birth by caesarean section. | All selected females. | (102) |
|  |  | Rate of cesarean deliveries among standard primigravidae | Number of CS deliveries | Total standard primigravidae | (64) |
| **Caesarean section**  *(continued)* | Caesarean section rate, nulliparous  *(continued)* | Rate of cesarean sections in nulliparous women delivering a term, singleton baby in vertex position | Number of cesarean deliveries in NTSV cases | Total NTSV deliveries | (85) |
|  |  | Rate of cesareans for first-time mothers at term with a single, head-down fetus | Number of NTSV cesarean deliveries | Total NTSV deliveries | (65) |
|  |  | Standard primiparae who undergo caesarean section | Total number of standard primiparae who undergo caesarean section | Total number of standard primiparae | (104) |
| **Treatment, caesarean section-related** | Umbilical artery pH | Performing umbilical artery ph after Caesarean Section | Number of mothers offered Caesarean section and umbilical artery ph after Caesarean Section | Number of mothers offered Caesarean section | (50) |
|  | DVT prophylaxis | Pneumatic compression and/or Compression stockings after Caesarean Section | Number of mothers offered Caesarean section and pneumatic compression devices | Number of mothers offered Caesarean section | (50) |
|  |  | Proportion of cesarean deliveries with DVT prophylaxis using Sequential Compression Device (SCD) | Number of cesarean deliveries with SCD prophylaxis | Total cesarean deliveries | (65) |
|  | Antiemetics | Offering antiemetics for Caesarean Section | Number of mothers offered selective Caesarean section and antiemetics | Number of mothers offered selective Caesarean section | (50) |
|  | Oxytocin injection | Oxytocin intravenous injection for Caesarean Section | Number of mothers offered selective Caesarean section and Oxytocin intravenous injection | Number of mothers offered selective Caesarean section | (50) |
|  | Antimicrobials | Appropriate use of antimicrobials for caesarean section | Number of patients who underwent cesarean surgery with recommended antibiotic prophylaxis | Number of patients who underwent cesarean surgery | (50) |
|  |  | Appropriate use of antimicrobials for caesarean section (Cefazolin sodium hydrate) | Number of patients who underwent cesarean with first-line (CEZ) antibiotic prophylaxis | Number of patients who underwent cesarean surgery | (50) |
| **Management, PPH** | Hemostatic surgery (other than hysterectomy) | Hemostatic surgery (other than hysterectomy) | Number of hemostatic surgeries other than hysterectomies | Total number of deliveries | (86) |
|  | Hysterectomy | Hemostatic hysterectomies | Number of hemostatic hysterectomies | Total number of deliveries | (86) |
|  |  | People having a peripartum hysterectomy | Total number of postpartum people having an abdominal hysterectomy within six weeks after birth | Total number of people giving birth | (104) |
|  |  | Peripartum hysterectomy | Number of pregnancies complicated by this measure | Total number of deliveries for that time period | (95) |
|  |  | Postpartum hysterectomy | Number of cases | Time period | (47) |
|  | Postpartum haemorrhage management | Appropriate care provided for patients with PPH of 500–1000 ml and no signs of shock | Number of PPH patients receiving appropriate management | Total number of patients with PPH (500-1000ml) | (61) |
|  |  | Care provided to patients with severe PPH (1000-2000 ml or signs of shock) | Number of severe PPH patients receiving appropriate care | Total number of patients with severe PPH | (61) |
|  |  | In case of blood loss >500 ml without signs of shock, homebirth, and blood loss not ceasing: refer to secondary care | The number of women in whom a specific test or intervention has been performed | The number of women in whom this test or intervention should have been performed | (52) |
| **Management, PPH**  *(continued)* | Postpartum haemorrhage management  *(continued)* | In case of blood loss >500 ml without signs of shock, homebirth, and retained placenta: refer to secondary care after 30 minutes | The number of women in whom a specific test or intervention has been performed | The number of women in whom this test or intervention should have been performed |  |
|  |  | In case of blood loss >500 ml without signs of shock, midwifery supervised hospital birth, and blood loss not ceasing: refer to secondary care | The number of women in whom a specific test or intervention has been performed | The number of women in whom this test or intervention should have been performed |  |
|  |  | In case of blood loss >500 ml without signs of shock, midwifery supervised hospital birth, and retained placenta: refer to secondary care after 30 minutes | The number of women in whom a specific test or intervention has been performed | The number of women in whom this test or intervention should have been performed |  |
|  |  | In case of blood loss >500 ml without signs of shock, post placental, and if blood loss is not ceasing despite administration of uterotonics: examine for vaginal and perineal lesions | The number of women in whom a specific test or intervention has been performed | The number of women in whom this test or intervention should have been performed |  |
|  |  | In case of blood loss >500 ml without signs of shock, treat PPH as uterine atony (and apply bladder catheterization, uterine massage and oxytocin) until proven otherwise | The number of women in whom a specific test or intervention has been performed | The number of women in whom this test or intervention should have been performed |  |
|  |  | In case of blood loss >500 ml, without signs of shock, measure blood loss by weighing | The number of women in whom a specific test or intervention has been performed | The number of women in whom this test or intervention should have been performed |  |
|  |  | In case of persisting PPH of > 1000 ml with signs of shock and/or >2000 ml blood loss, perform uterine and/ or aortal compression | The number of women in whom a specific test or intervention has been performed | The number of women in whom this test or intervention should have been performed |  |
|  |  | In case of PPH of >1000 ml and/or signs of shock, monitor vital signs frequently (pulse, blood pressure, respiratory frequency) | The number of women in whom a specific test or intervention has been performed | The number of women in whom this test or intervention should have been performed |  |
|  |  | In case of PPH of >1000 ml and/or signs of shock, regardless of oxygen saturation, provide patient with 10–15 litre oxygen via non-rebreathing mask | The number of women in whom a specific test or intervention has been performed | The number of women in whom this test or intervention should have been performed |  |
|  |  | In case of PPH of >1000 ml and/or signs of shock, start an intravenous line and supply with fluids, using 0, 9% sodium chloride | The number of women in whom a specific test or intervention has been performed | The number of women in whom this test or intervention should have been performed |  |
|  |  | In case of PPH of >1000 ml and/or signs of shock: the midwife should inform the secondary caregiver (obstetrician) | The number of women in whom a specific test or intervention has been performed | The number of women in whom this test or intervention should have been performed | (52) |
|  |  | In case of PPH of > 1000 ml with signs of shock and/or >2000 ml blood loss, if the patient has reduced consciousness due to hypovolemic shock: call for (paramedic) assistance in order to establish an open airway | The number of women in whom a specific test or intervention has been performed | The number of women in whom this test or intervention should have been performed | (52) |
| **Management, PPH**  *(continued)* | Postpartum haemorrhage management  *(continued)* | In case of PPH of > 1000 ml with signs of shock and/or >2000 ml blood loss, immediately transfer patient to secondary care | The number of women in whom a specific test or intervention has been performed | The number of women in whom this test or intervention should have been performed | (52) |
|  |  | In case of PPH of > 1000 ml with signs of shock and/or >2000 ml blood loss, secure a second intravenous line (14 gauge) | The number of women in whom a specific test or intervention has been performed | The number of women in whom this test or intervention should have been performed | (52) |
|  |  | Non-pharmaceutical curative second line procedures performed for PPH after cesareans. | Non-pharmaceutical curative second line procedures (intrauterine balloon tamponade, radiologic arterial embolization, surgical procedures, and transfusion of packed red blood cells) performed for PPH | Caesarean deliveries | (36) |
|  |  | Non-pharmaceutical curative second-line procedures performed for PPH after vaginal deliveries | Non-pharmaceutical curative second-line procedures (intrauterine balloon tamponade, radiologic arterial embolization, surgical procedures, and transfusion of packed red blood cells) performed for PPH | Vaginal deliveries | (36) |
|  |  | Number of women given sulprostone to manage PPH | Number of women given sulprostone | Total number of women | (75) |
|  |  | Rate of postpartum hemorrhage treated by one or more of embolization, surgery, or transfusion of more than four units of packed red blood cells among all women | Rate of postpartum hemorrhage treated by one or more of embolization, surgery, or transfusion of more than four units of packed red blood cells | All women | (76) |
|  |  | Rate of postpartum hemorrhage treated by one or more of embolization, surgery, or transfusion of more than four units of packed red blood cells, among women at low risk of PPH | Rate of postpartum hemorrhage treated by one or more of embolization, surgery, or transfusion of more than four units of packed red blood cells | Women at low risk of PPH: singleton at term, with no abnormal placental insertion, no history of cesarean, and no preeclampsia | (76) |
|  |  | Accurate monitoring of blood loss during labor and after delivery | Accurate monitoring of blood loss during labor and after delivery | Total number of patients | (61) |
|  |  | Administer 10–15 liters oxygen via non-rebreather mask regardless of saturation level | Women receiving oxygen | Women with PPH > 1000 ml | (83) |
|  |  | Administer uterine massage alongside catheter use and oxytocin | Women receiving uterine massage | Women with uterine atony | (83) |
|  |  | Administration of at least 5 IU oxytocin intramuscular within 3 minutes post-birth | Women given oxytocin | Women eligible for oxytocin | (83) |
|  |  | Ensure an IV line during labor for patients at high risk of PPH | Number of high-risk patients with IV line | Total number of high-risk PPH patients | (61) |
|  |  | Measurement by weighing blood loss, especially in cases over 500 ml | Cases with blood weighed | Total PPH cases | (83) |
|  |  | Prevention of PPH: antenatally, the midwife should identify elevated- or high risk of PPH and agree on preventive strategies | The number of women in whom a specific test or intervention has been performed | The number of women in whom this test or intervention should have been performed | (52) |
| **Management, PPH**  *(continued)* | Postpartum haemorrhage management  *(continued)* | Prevention of PPH: at birth, the midwife should identify elevated- or high risk of PPH and agree (or adjust) preventive strategies | The number of women in whom a specific test or intervention has been performed | The number of women in whom this test or intervention should have been performed | (52) |
|  |  | Prevention of PPH: if high risk of PPH is assessed, have birth occur in hospital supervised by the obstetrician. | The number of women in whom a specific test or intervention has been performed | The number of women in whom this test or intervention should have been performed | (52) |
|  |  | Prevention of PPH: routinely administer uterotonics (at least 5 IU oxytocin intramuscular). | The number of women in whom a specific test or intervention has been performed | The number of women in whom this test or intervention should have been performed | (52) |
|  |  | Women receiving uterotonics for the prevention of postpartum haemorrhage during the third stage of labour | Women receiving uterotonics for the prevention of postpartum haemorrhage during the third stage of labour | Women had vaginal deliveries | (30) |
| **Blood transfusion** | Blood transfusion | Blood transfusion during and/or after delivery | Number of women given blood transfusions during and/or after delivery | Total number of women delivered | (87) |
|  |  | Blood transfusions in delivery or operating room | Number of blood transfusions in the delivery or operating room | Total number of deliveries | (86) |
|  |  | Need for blood transfusion postpartum | Patients receiving transfusions | Total deliveries | (106) |
|  |  | Number of women receiving a blood transfusion | Number of transfused women | Total number of women | (75) |
|  |  | People requiring a blood transfusion with caesarean section | People requiring a blood transfusion with caesarean section | Total number of people who undergo caesarean section | (104) |
|  |  | People requiring a blood transfusion with vaginal birth | Total number of people requiring a blood transfusion with vaginal birth | Total number of people who give birth vaginally | (104) |
|  | Maternal transfusion incident rate | Maternal transfusion incident rate | Maternal transfusion incident rate | All women | (76) |
| **Screening, maternal** | Depression screening | Maternal mental health postpartum | Number of positive screens | Total number of postpartum women screened | (20) |
|  |  | Postpartum depression screening: Proportion of women who were screened for postpartum depression after a live birth | Proportion of women who were screened for postpartum depression | Women after a live birth | (60) |
|  |  | Women being screened for antenatal or postnatal depression using a validated questionnaire | Women being screened for antenatal or postnatal depression using a validated questionnaire | Pregnant women | (30) |
|  | Proportion of women delivering at term who had GBS screening at 35 to 37 weeks’ gestation | Proportion of women delivering at term who had Group B Streptococcus (GBS) screening at 35 to 37 weeks’ gestation | The number of women having an unplanned Caesarean section in labour who deliver at term and have GBS screening at 35 to 37 weeks’ gestation | The total number of labouring women delivering at term (in a given place and time) | (72) |

***Indicator constructs:** Umbrella concepts aggregating multiple related indicator groups. **^$^Standardised indicator labels:** Primary analytical units clustering individual indicators with closely related definitions.
