# Supplementary material for: Monitoring childbirth and immediate postpartum care quality: A scoping review of indicators in high-income countries
Source: PLoS One. 2026 Sep 15;21(9):e0350665. doi: 10.1371/journal.pone.0350665 (PMC13577461; doi:10.1371/journal.pone.0350665)
Supplement: Supplementary file 10 [file pone.0350665.s011.docx]

**S8 Table. List of complete neonatal process indicators (N=12).**

| **Indicator Constructs***  **Neonatal** | **Standardised Indicators^$^** | **Name/ definition of indicator *per source*** | **Numerator *per source*** | **Denominator *per source*** | **References** |
| --- | --- | --- | --- | --- | --- |
| **Resuscitation** | Japanese Neonatal Resuscitation Algorithm | Infants offered the necessary resuscitation in the first minutes after birth, evaluating their condition in line with the Japanese Neonatal Resuscitation Algorithm | Infants offered the necessary resuscitation in the first minutes after birth, evaluating their condition in line with the Japanese Neonatal Resuscitation Algorithm | Infants evaluated to offer the necessary resuscitation in the first minutes after birth | (30) |
| **Treatment, neonatal** | Avoiding administration of dexamethasone with non-steroidal anti-inflammatory drugs (NSAIDs). | Avoiding administration of dexamethasone with non-steroidal anti-inflammatory drugs (NSAIDs). | Number of premature neonates (born at <37 gestational week) who offered steroids without nonsteroidal anti-inflammatory drugs at the same day | Number of premature neonates (born at <37 gestational week) who offered steroids | (50) |
|  | Budesonide inhalation for neonates at very high risk of Bronchopulmonary Dysplasia (BPD) | Budesonide inhalation for neonates at very high risk of Bronchopulmonary Dysplasia (BPD) | Number of neonates at very high risk of BPD and offered Budesonide inhalation | Number of neonates at very high risk of BPD (required respiratory support or Oxygen at 28 days after birth) | (50) |
|  | Caffeine administration to facilitate weaning from mechanical ventilation | Caffeine administration to facilitate weaning from mechanical ventilation | Number of neonates who used and weaned from mechanical ventilation at <33 adjusted gestational week and offered Caffeine when weaning | Number of neonates who used and weaned from mechanical ventilation at <33 adjusted gestational week | (50) |
|  | Caffeine citrate for preterm neonates born at <31 gestational weeks | Caffeine citrate for preterm neonates born at <31 gestational weeks | Number of premature neonates (born at <31 gestational week) and offered Caffeine citrate within 3 days after birth | Number of premature neonates (born at <31 gestational week) | (50) |
|  | Continuous positive airways pressure (CPAP) at admission for infants at 32-36 weeks' postmenstrual age | Continuous positive airways pressure (CPAP) at admission for infants at 32-36 weeks' postmenstrual age | Number of premature neonates (born at 32-36 gestational week) who required respiratory support and offered CPAP instead of mechanical ventilation | Number of premature neonates (born at 32-36 gestational week) who required respiratory support | (50) |
|  | Culture test in broad spectrum antibacterial agents | Culture test in broad spectrum antibacterial agents | Number of children aged ≤18 who administrated broad spectrum antibacterial agents with culture test | Number of children aged ≤18 who administrated broad spectrum antibacterial agents | (50) |
|  | Vitamin K administration | Infants administered vitamin K three times by one month after birth | Infants administered vitamin K | Infants who didn’t admit to paediatrics department | (30) |
| **Respiratory support** | Oxygen Dependence | Oxygen required beyond 24 hours of life | Number of neonates requiring oxygen support | Total number of live births | (20) |
|  | Respiratory support | Respiratory support: Resuscitation for asphyxiated term neonate with low oxygen concentrations and oxygen saturation measured by pulse oximetry immediately after birth | Respiratory support | Infants with asphyxia after birth | (30) |
|  | Respiratory support at term | Babies born at 37+ weeks’ gestation requiring respiratory support | Total number of babies born at 37+ weeks’ gestation requiring over 4 hours of respiratory support | Total number of babies born at 37+ weeks’ gestation | (104) |
| **Screening, neonatal** | Proportion of newborn screening samples that are unsatisfactory for testing | Proportion of newborn screening samples that are unsatisfactory for testing | The number of newborn screening samples that were unsatisfactory for testing | Total number of newborn screening samples submitted to Newborn Screening Ontario (NSO) from a given hospital | (72) |

***Indicator constructs:** Umbrella concepts aggregating multiple related indicator groups.

**^$^Standardised indicator labels:** Primary analytical units clustering individual indicators with closely related definitions.
