# Supplementary material for: Monitoring childbirth and immediate postpartum care quality: A scoping review of indicators in high-income countries
Source: PLoS One. 2026 Sep 15;21(9):e0350665. doi: 10.1371/journal.pone.0350665 (PMC13577461; doi:10.1371/journal.pone.0350665)
Supplement: Supplementary file 11 [file pone.0350665.s012.docx]

**S9 Table.** **List of complete maternal and neonatal process indicators (N= 21).**

| **Indicator Constructs*** | **Standardised Indicators^$^** | **Name/ definition of indicator *per source*** | **Numerator *per source*** | **Denominator *per source*** | **References** |
| --- | --- | --- | --- | --- | --- |
| **Infant feeding** | Breastfeeding | Breastfeeding has significant health benefits for infants, and initiation rates are key for public health outcomes. | Number of infants breastfed at any point and exclusively at six months | Total number of live births | (78) |
|  |  | Decision to breastfeed at discharge | Number of women who decided to breastfeed at discharge | Total number of women discharged home with a live baby | (87) |
|  |  | Percentage of babies whose first feed is breastmilk | Number of live babies born in the period whose mothers are resident within the local authority (residence based on mother's postcode), whose first feed is known to be breastmilk, including expressed and donor milk | Number of live babies born in the period whose mothers are resident within the local authority (residence based on mother's postcode) whose first feed status is known | (100) |
|  |  | Percentage of breastfed babies at the end of the midwifery care | Number of neonates being breastfed at the end of midwifery care | Total number of neonates under midwifery care | (59) |
|  |  | The number of babies who are partially breastfed (infant receives breast milk and the infant is allowed any food or liquid including non-human milk) and the number who are exclusively breastfed (infant receives breast milk and is allowed to receive drops and syrups) throughout the first 48 hrs of age as a proportion of all newborn babies. (Definitions from WHO Indicators for Assessing Breastfeeding Practices. Report from meeting 11-12 June 1991. Geneva, 1991.) | The number of babies who are partially breastfed (infant receives breast milk and the infant is allowed any food or liquid including non-human milk) and the number who are exclusively breastfed (infant receives breast milk and is allowed to receive drops and syrups) throughout the first 48 hrs of age | All newborn babies | (9) |
|  |  | The number of babies who are partially breastfed (infant receives breast milk and the infant is allowed any food or liquid including non-human milk) and the number who are exclusively breastfed (infant receives breast milk and is allowed to receive drops and syrups) throughout the first 48 hrs of age as a proportion of all newborn babies. (Definitions from WHO Indicators for Assessing Breastfeeding Practices. Report from meeting 11-12 June 1991. Geneva, 1991.) | The number of babies who are partially breastfed (infant receives breast milk and the infant is allowed any food or liquid including non-human milk) and the number who are exclusively breastfed (infant receives breast milk and is allowed to receive drops and syrups) throughout the first 48 hrs of age | As a proportion of all newborn babies | (81) |
| **Infant feeding**  *(continued)* | Breastfeeding *(continued)* |  |  |  |  |
|  | Exclusive breastfeeding/breastmilk | Exclusive breast milk feeding during the newborn's entire hospitalization. The measure is reported as an overall rate which includes all newborns that were exclusively fed breast milk during the entire hospitalization. | Newborns that were fed breast milk only since birth | Single term newborns discharged alive from the hospital | (105) |
|  |  | Infants that were fed only breast milk at the time of discharge from the hospital | Feeding only breast milk at the time of discharge from the hospital | Infants who didn’t admit to paediatrics department or didn’t need to supply formula with medical evidence | (30) |
|  |  | Mothers who report exclusive breastfeeding for 7 days | Number of mothers exclusively breastfeeding | Total number of postpartum women | (20) |
|  |  | Percentage of infants exclusively breastfed from birth until discharge | Number of newborns exclusively breastfed | Total live births | (65) |
|  |  | Percentage of newborns exclusively breastfed at discharge | Number of newborns exclusively breastfed | Total eligible newborns | (85) |
|  |  | Proportion of women who gave birth to a live-born child and exclusively breastfed the child with no other liquid or solid food, for at least the first six or more months of life, among women who gave birth in the last five years. | Number of women aged 15-55 years who gave birth in the last five years and have exclusively breastfed their child with no other liquid or solid food, for six or more months. | Number of women aged 15-55 years who gave birth in the last five years. | (103) |
|  | Formula supplementation | Infants given formula supplementation without medical rationale from birth to discharge in term infants, even though the woman intended to breastfeed | Formula supplementation without medical rationale during hospitalization | Infants who didn’t admit to paediatrics department or didn’t need to supply formula with medical evidence | (30) |
|  |  | Rate of formula supplementation in term infants whose mothers intended to breastfeed | Number of term live babies receiving formula supplementation | The total number of term babies whose mothers intended to breastfeed (in a given place and time). | (72) |
| **Delivery, vaginal** | Planned vaginal delivery (twins) | Twin deliveries where the intention was vaginal birth based on clinical assessment. Includes both successful vaginal deliveries and cases that shifted to intrapartum CS. | Number of of twin pregnancies initially intended for vaginal delivery. | Total number of twin pregnancies | (40) |
|  | Vaginal delivery (twins) | Twin deliveries where both twins were successfully delivered vaginally as initially intended | Number of twin pregnancies with successful vaginal delivery of both twins | Total number of twin pregnancies | (40) |
|  | Vaginal delivery, nulliparous | First time vaginal deliveries without cesarean section, vacuum extraction or forceps, episiotomy, 3rd or 4th degreee lacerations, post-partum Hemorrhage >1000ml and Apgar score ≥9/5 | First time vaginal deliveries without cesarean section, vacuum extraction or forceps, episiotomy, 3rd or 4th degreee lacerations, post-partum Hemorrhage >1000ml and Apgar score ≥9/5 | All first time intended vaginal deliveries | (71) |
|  | Vaginal Uncomplicated Delivery Rate (VUD Rate) | All vaginal deliveries without maternal or neonatal adverse outcomes | Vaginal deliveries with no AOI-defined events | Total deliveries by obs | (106) |
| **Early skin-to-skin contact** | Early skin-to-skin contact | A definition of one hour of skin-to-skin contact within the first two hours post-partum was chosen. Live, singleton births with Apgar score ≥ 9 | Deliveries where skin-to-skin contact between mother and the newborn child is established | All deliveries | (71) |
|  |  | Women having early skin-to-skin contact with their babies if they wish, soon after birth in secure surroundings | Women having early skin-to-skin contact with their babies if they wish, soon after birth in secure surroundings | Pregnant women wished to make early skin-to-skin contact with their babies soon after birth in secure surroundings | (30) |
| **Delivery, combined** | Combined Delivery of twins (Twin A Vaginal, Twin B by Intrapartum CS) | Rate of twin deliveries where twin A is delivered vaginally and twin B by intrapartum cesarean | Number of deliveries where twin A was delivered vaginally and twin B by intrapartum cesarean | Total number of twin pregnancies with planned vaginal delivery | (40) |
