# Supplementary material for: Monitoring childbirth and immediate postpartum care quality: A scoping review of indicators in high-income countries
Source: PLoS One. 2026 Sep 15;21(9):e0350665. doi: 10.1371/journal.pone.0350665 (PMC13577461; doi:10.1371/journal.pone.0350665)
Supplement: Supplementary file 12 [file pone.0350665.s013.docx]

**S10 Table. List of complete structure and access indicators (N = 50).**

| **Indicator Constructs*** | **Standardised Indicators^$^** | **Name/ definition of indicator *per source*** | **Numerator *per source*** | **Denominator *per source*** | **References** |
| --- | --- | --- | --- | --- | --- |
| **Organisation** | Postpartum haemorrhage management | A regional PPH protocol should be the basis of regular audits | The number of women in whom a specific test or intervention has been performed | The number of women in whom this test or intervention should have been performed | (52) |
|  |  | % of hospitals with protocols in place and being used | Number of hospitals with active PPH protocols | Total number of hospitals surveyed | (61) |
|  |  | Within every regional obstetric collaboration, a regional PPH protocol should be present, based on national guidelines | The number of women in whom a specific test or intervention has been performed | The number of women in whom this test or intervention should have been performed | (52) |
|  |  | The midwife is aware that ambulance transportation in case of PPH or retained placenta is always of the highest urgency category | The number of women in whom a specific test or intervention has been performed | The number of women in whom this test or intervention should have been performed | (52) |
|  | Women and infants having complete medical records based on all quality indicator | Women and infants having complete medical records based on all quality indicator | Women and infants having complete medical records based on all quality indicator | Women and infants who didn’t admitting within 24 h | (30) |
|  | Quality system availability in midwifery practice | Availability of a quality system in the midwifery practice | Number of practices with a formal quality system | Total number of midwifery practices | (59) |
|  | Participation in Regional Organization of Midwives | Active participation in the regional organization of midwives | Number of midwives actively participating in a regional organization | Total number of midwives in the region | (59) |
|  | Regional Obstetric Collaboration Group participation rate | Active participation in the regional Obstetric Collaboration Group of professionals involved in obstetrics (OCG) | Number of professionals actively participating in collaboration group | Total eligible professionals in the region | (59) |
|  | Access to care | In a homebirth situation, anticipation on possible ambulance transport is necessary; make sure the patient is at an accessible place for (all) caregivers in time | The number of women in whom a specific test or intervention has been performed | The number of women in whom this test or intervention should have been performed | (52) |
|  | 24/7 backup procedure | A procedure for backup duty 7x24h a week | Number of practices with 24/7 backup procedures in place | Total number of midwifery practices | (59) |
|  | Protocol for Child Health Center Referral | Availability of a protocol for referral to the Child Health Centre | Number of practices with a referral protocol to Child Health Centers | Total number of midwifery practices | (59) |
|  | Women switched to receive care provided primarily by obstetricians from midwifery ward | Women switched to receive care provided primarily by obstetricians from midwifery ward | Women switched to receive care provided primarily by obstetricians from midwifery ward | Pregnant women | (30) |
|  | Compliance with the requirements for a safe delivery (proxy measurement) | Percentage of women with planned vaginal birth attended by a skilled attendant during labor | Women with planned  vaginal birth attended by a skilled attendant during labor | All women giving birth | (37) |
|  | Number of births and postpartum stays | Number of births and postpartum stays that occur per year in the birth centre | Number of births and postpartum stays that occur | Per year in the birth centre | (46) |
| **Evaluation** | Evaluation of care in case of (near) accidents | Evaluation of midwifery care in the case of (near) accidents | Number of near-accidents reviewed and evaluated | Total number of near-accidents reported | (59) |
|  | Accreditation of midwifery practice | Accreditation of Midwifery Practice | Number of accredited midwifery practices | Total number of midwifery practices | (59) |
|  | Missing or unavailable patients records, such as test results, during delivery | Missing or unavailable patients records, such as test results, during delivery | Number of cases with missing records | Total deliveries | (79) |
|  | Postpartum haemorrhage management | Reliable records to track adherence to PPH protocols and guidelines | Cases with complete records | Total PPH cases | (83) |
|  | Availability of complaint regulation | Methods of complaint regulation | Number of practices with a formal complaint procedure | Total number of midwifery practices | (59) |
|  | Client experience surveys | Percentage received filled-in questionnaires to explore client experiences of midwifery care | Number of clients providing feedback through surveys | Total number of clients under midwifery care | (59) |
|  | Peer review of severe adverse events with medical staff | Peer review of severe adverse events with medical staff | Peer review of severe adverse events with medical staff | Women of infant with severe adverse events | (30) |
|  | Postpartum haemorrhage >2000 mL with debrief | After each PPH with >2000 mL blood loss, the multidisciplinary team should debrief the situation | The number of women in whom a specific test or intervention has been performed | The number of women in whom this test or intervention should have been performed | (52) |
|  | Prevention of planned CS by offering external cephalic version in case of non-cephalic position | Adherence to recommendations: prevention of planned CS by offering external cephalic version in case of non-cephalic position | Number of women to whom the indicator applied and actual care was consistent with the indicator | Total number of women to whom the indicator applied | (48) |
|  | Partogram use | Percentage of deliveries in midwifery care, recorded by means of a partogram | Number of labors monitored using a partogram | Number of labors monitored using a partogram | (59) |
|  |  | Unplanned CS: use of partogram | Number of women to whom the indicator applied and actual care was consistent with the indicator | Total number of women to whom the indicator applied | (48) |
| **Resources** | Workforce: Supervisor/midwife ratio | Supervisor/midwife ratio | Supervisors | Midwifes | (47) |
|  | Workforce: Midwife/patient ratio | Midwife/patient ratio | Midwifes | Patients | (47) |
|  | Number of registered midwives in quality register | Number of midwives registered in the quality register of the professional group | Number of midwives (gps) working in the practice concerned and registered in the quality register | Number of midwives (gps) working in the practice concerned | (59) |
|  | Physician response to an urgent request within 30 minutes | % of urgent requests associated with a response time of less than 30 minutes | Number of urgent requests associated with a response time of less than 30 minutes | Overall number of urgent requests | (49) |
|  | Continuous support for women in the delivery room | Proportion of women who have a healthcare professional continuously in the delivery room during the active phase of labor.  Standard: At least 90% | Women with healthcare professional continuously present in the delivery room | All women with intended vaginal delivery | (71) |
|  | Unassisted births | Percentage of unassisted births in the case of too late arrival of the attending midwife or GP | Number of unassisted births due to late arrival of professional | Total births planned with professional attendance | (59) |
| **Resources**  *(continued)* | Home birth | Percentage of home deliveries with attendance of a maternity assistant | Number of home deliveries attended by a professional (e.g., midwife, GP) | Total number of home deliveries | (59) |
|  | Annual PPH training | Ensures readiness and updated practices in handling PPH | Midwives attending training | Total midwives | (83) |
|  | Caesarean section, unplanned | Unplanned CS: continuous support during labor for women with or without prior training | Number of women to whom the indicator applied and actual care was consistent with the indicator | Total number of women to whom the indicator applied | (48) |
| **Communication** | Staff Communication Issues | Tracks breakdowns in staff communication potentially affecting care quality | Number of reported communication issues | Total QIN reports or deliveries | (79) |
|  | Debrief with maternity staff | Women having a review of their childbirth experience and support with the midwives and other staff who assisted at the birth | Women having a review of their childbirth experience and support with the midwives and other staff who assisted at the birth | Pregnant women | (30) |
| **Counselling on mode of delivery** | CS not mentioned during counseling on the mode of delivery | Adherence to recommendations: CS not mentioned during counseling on the mode of delivery for cases of fetal macrosomia (<4.5kg in maternal diabetes, <5kg no maternal diabetes) | Number of women to whom the indicator applied and actual care was consistent with the indicator | Total number of women to whom the indicator applied | (48) |
|  |  | Adherence to recommendations: CS not mentioned during counseling on the mode of delivery for cases of preterm labour and cephalic position | Number of women to whom the indicator applied and actual care was consistent with the indicator | Total number of women to whom the indicator applied |  |
|  |  | Adherence to recommendations: CS not mentioned during counseling on the mode of delivery for cases of previous shoulder dystocia without impaired perinatal outcome | Number of women to whom the indicator applied and actual care was consistent with the indicator | Total number of women to whom the indicator applied |  |
|  |  | Adherence to recommendations: CS not mentioned during counseling on the mode of delivery for cases of small for gestational age without fetal distress | Number of women to whom the indicator applied and actual care was consistent with the indicator | Total number of women to whom the indicator applied |  |
|  |  | Adherence to recommendations: CS not mentioned during counseling on the mode of delivery for cases of twin pregnancy and first child cephalic position | Number of women to whom the indicator applied and actual care was consistent with the indicator | Total number of women to whom the indicator applied |  |
|  | VD preferred during counseling on the mode of delivery | Adherence to recommendations: VD preferred during counseling on the mode of delivery for cases of placental position at 1-2cm of the internal OS | Number of women to whom the indicator applied and actual care was consistent with the indicator | Total number of women to whom the indicator applied |  |
|  |  | Adherence to recommendations: VD preferred during counseling on the mode of delivery for cases of preterm breech delivery (frank, complete breech) | Number of women to whom the indicator applied and actual care was consistent with the indicator | Total number of women to whom the indicator applied |  |
| **Counselling on mode of delivery**  *(continued)* | VD preferred during counseling on the mode of delivery  *(continued)* | Adherence to recommendations: VD preferred during counseling on the mode of delivery for cases of request for CS without medical grounds (explore reason for request, discuss (dis)advantages to CS delivery, offer psychological counseling if extreme fear) | Number of women to whom the indicator applied and actual care was consistent with the indicator | Total number of women to whom the indicator applied |  |
|  | VD and CS mentioned as equal options during counseling on the mode of delivery for cases of previous CS and medical reason for induction of labour | Adherence to recommendations: VD and CS mentioned as equal options during counseling on the mode of delivery for cases of previous CS and medical reason for induction of labour | Number of women to whom the indicator applied and actual care was consistent with the indicator | Total number of women to whom the indicator applied | (48) |
|  | VD and CS mentioned as equal options during counseling on the mode of delivery for cases of breech presentation at term | Adherence to recommendations: VD and CS mentioned as equal options during counseling on the mode of delivery for cases of breech presentation at term | Number of women to whom the indicator applied and actual care was consistent with the indicator | Total number of women to whom the indicator applied | (48) |
|  | VD and CS mentioned as equal options during counseling on the mode of delivery for cases of previous CS | Adherence to recommendations: VD and CS mentioned as equal options during counseling on the mode of delivery for cases of previous CS | Number of women to whom the indicator applied and actual care was consistent with the indicator | Total number of women to whom the indicator applied | (48) |
| **Access** | Intrapartum referral | Percentage of intrapartum referral | Number of intrapartum referrals | Total women in labor under midwifery care | (59) |
|  |  | Percentage of referrals due to slow progress of labour or need for pain relief | Number of referrals due to labor progress issues or need for pain relief | Total number of women under midwifery care during labor | (59) |
|  | Accessibility for non-urgent inquiries | Accessibility to midwifery advice and information for non-urgent matters | Total weekly hours during which midwifery services are available for non-urgent inquiries. | N/A (indicator (expressed as a total number of hours) | (59) |

***Indicator constructs:** Umbrella concepts aggregating multiple related indicator groups.

**^$^Standardised indicator labels:** Primary analytical units clustering individual indicators with closely related definitions.
