# Supplementary material for: Monitoring childbirth and immediate postpartum care quality: A scoping review of indicators in high-income countries
Source: PLoS One. 2026 Sep 15;21(9):e0350665. doi: 10.1371/journal.pone.0350665 (PMC13577461; doi:10.1371/journal.pone.0350665)
Supplement: Supplementary file 13 [file pone.0350665.s014.docx]

Dear Editor,

We sincerely thank the Editors and Reviewers for their careful evaluation of our manuscript. We have revised the article accordingly and addressed all the comments raised.

**Editor comments to Author:**

**When submitting your revision, we need you to address these additional requirements.**

**1.Please ensure that your manuscript meets PLOS ONE's style requirements, including those for file naming. The PLOS ONE style templates can be found at https://journals.plos.org/plosone/s/file?id=wjVg/PLOSOne_formatting_sample_main_body.pdf andhttps://journals.plos.org/plosone/s/file?id=ba62/PLOSOne_formatting_sample_title_au thors_affiliations.pdf**

We thank the Editor for this comment. We have carefully checked the manuscript against the PLOS ONE style requirements, including the formatting instructions and file naming guidelines.

All necessary modifications have been implemented to ensure full compliance with the journal’s requirements.

**2. Please note that funding information should not appear in any section or other areas of your manuscript. We will only publish funding information present in the Funding Statement section of the online submission form. Please remove any funding-related text from the manuscript.**

The funding section was removed as requested from the manuscript. Funding details are now provided exclusively in the Funding Statement section of the online submission form.

**3. We note that the grant information you provided in the ‘Funding Information’ and ‘Financial Disclosure’ sections do not match. When you resubmit, please ensure that you provide the correct grant numbers for the awards you received for your study in the ‘Funding Information’ section.**

Thank you for this comment. We have updated both the ‘Funding Information’ and ‘Financial Disclosure’ sections as requested to match and included the grant information.

**4. We note that your Data Availability Statement is currently as follows: All relevant data are within the manuscript and in Supporting Information files.**

**Please confirm at this time whether or not your submission contains all raw data required to replicate the results of your study. Authors must share the “minimal data set” for their submission. PLOS defines the minimal data set to consist of the data required to replicate all study findings reported in the article, as well as related metadata and methods (**[**https://journals.plos.org/plosone/s/data-availability#loc-minimal-data-set-definition**](https://ita01.safelinks.protection.outlook.com/?url=https%3A%2F%2Fjournals.plos.org%2Fplosone%2Fs%2Fdata-availability%23loc-minimal-data-set-definition&data=05%7C02%7C%7Cb74c07a7044e4bad4f0708debbb6bce6%7C84df9e7fe9f640afb435aaaaaaaaaaaa%7C1%7C0%7C639154590688058321%7CUnknown%7CTWFpbGZsb3d8eyJFbXB0eU1hcGkiOnRydWUsIlYiOiIwLjAuMDAwMCIsIlAiOiJXaW4zMiIsIkFOIjoiTWFpbCIsIldUIjoyfQ%3D%3D%7C0%7C%7C%7C&sdata=CM3bNa09%2F%2FbJiIadsor1lqtjUNm8MbUlyZYq%2BS3%2BC54%3D&reserved=0)**).**

**For example, authors should submit the following data:**

**- The values behind the means, standard deviations and other measures reported;**

**- The values used to build graphs;**

**- The points extracted from images for analysis.**

**Authors do not need to submit their entire data set if only a portion of the data was used in the reported study.**

**If your submission does not contain these data, please either upload them as Supporting Information files or deposit them to a stable, public repository and provide us with the relevant URLs, DOIs, or accession numbers. For a list of recommended repositories, please see** [**https://journals.plos.org/plosone/s/recommended-repositories**](https://ita01.safelinks.protection.outlook.com/?url=https%3A%2F%2Fjournals.plos.org%2Fplosone%2Fs%2Frecommended-repositories&data=05%7C02%7C%7Cb74c07a7044e4bad4f0708debbb6bce6%7C84df9e7fe9f640afb435aaaaaaaaaaaa%7C1%7C0%7C639154590688079283%7CUnknown%7CTWFpbGZsb3d8eyJFbXB0eU1hcGkiOnRydWUsIlYiOiIwLjAuMDAwMCIsIlAiOiJXaW4zMiIsIkFOIjoiTWFpbCIsIldUIjoyfQ%3D%3D%7C0%7C%7C%7C&sdata=V2D2La0T5IxmmUN8jtJYfJgrD%2FCei5Ie03ZjPAnbd0s%3D&reserved=0)**.**

**If there are ethical or legal restrictions on sharing a de-identified data set, please explain them in detail (e.g., data contain potentially sensitive information, data are owned by a third-party organization, etc.) and who has imposed them (e.g., an ethics committee). Please also provide contact information for a data access committee, ethics committee, or other institutional body to which data requests may be sent. If data are owned by a third party, please indicate how others may request data access.**

We thank the Editor for this comment.

In accordance with PLOS ONE’s data availability requirements, we confirm that all materials necessary to reproduce our scoping review findings are provided within the manuscript and Supporting Information files. Specifically, we have amended the included studies list (S1 Table. Characteristics of included studies) to include the study design and reported data sources.

As this study is a scoping review, the minimal dataset consists of the complete list of included studies and the extracted study-level information used to identify, map, and classify childbirth and immediate postpartum care quality indicators.

The supplementary materials include the complete search strategy, the full list of included studies, and all extracted information required to reproduce the results, tables, and figures reported in the manuscript.

Consequently, we have amended the Data Availability Statement to better specify the minimal dataset supporting this scoping review. The revised statement now reads:

*“All data required to replicate the findings of this scoping review are available within the manuscript and Supporting Information files. The minimal dataset consists of the complete list of included studies and the extracted study-level information used to identify, map, and classify childbirth and immediate postpartum care quality indicators (S1 Table. Characteristics of included studies).”*

**5. Thank you for stating the following in the Competing Interests section:**

**“NO authors have competing interests”**

**We note that one or more of the authors are employed by a commercial company: Digital Workplace Squared, IT Skills and Solutions, Geneva, Switzerland**

**1.        Please provide an amended Funding Statement declaring this commercial affiliation, as well as a statement regarding the Role of Funders in your study. If the funding organization did not play a role in the study design, data collection and analysis, decision to publish, or preparation of the manuscript and only provided financial support in the form of authors' salaries and/or research materials, please review your statements relating to the author contributions, and ensure you have specifically and accurately indicated the role(s) that these authors had in your study. You can update author roles in the Author Contributions section of the online submission form.**

**Please also include the following statement within your amended Funding Statement.**

**“The funder provided support in the form of salaries for authors [insert relevant initials], but did not have any additional role in the study design, data collection and analysis, decision to publish, or preparation of the manuscript. The specific roles of these authors are articulated in the ‘author contributions’ section.”**

**If your commercial affiliation did play a role in your study, please state and explain this role within your updated Funding Statement.**

We thank the Editor for this comment. We have amended the Funding Statement to clarify the funding sources, the role of the funders, and the commercial affiliation of RM with Digital Workplace Squared, IT Skills and Solutions, Geneva, Switzerland.

The Funding Statement has been updated as follows:

*“TD received funding from the Fonds de Recherche Impulsion HES-SO (Grant number 127974) and the Swiss National Science Foundation (grant number PT0043_222882). The funders provided support in the form of salary support for author TD but did not have any additional role in the study design, data collection and analysis, decision to publish, or preparation of the manuscript.*

*“RM is employed by Digital Workplace Squared, IT Skills and Solutions, Geneva, Switzerland. The company provided support in the form of salary for author RM but had no additional role in the study design, data collection and analysis, decision to publish, or preparation of the manuscript. RM contributed to this work independently of her commercial affiliation. The specific roles of these authors are articulated in the ‘Author Contributions’ section”.*

**2. Please also provide an updated Competing Interests Statement declaring this commercial affiliation along with any other relevant declarations relating to employment, consultancy, patents, products in development, or marketed products, etc.**

**Within your Competing Interests Statement, please confirm that this commercial affiliation does not alter your adherence to all PLOS ONE policies on sharing data and materials by including the following statement: "This does not alter our adherence to  PLOS ONE policies on sharing data and materials.” (as detailed online in our guide for authors** [**http://journals.plos.org/plosone/s/competing-interests**](https://ita01.safelinks.protection.outlook.com/?url=http%3A%2F%2Fjournals.plos.org%2Fplosone%2Fs%2Fcompeting-interests&data=05%7C02%7C%7Cb74c07a7044e4bad4f0708debbb6bce6%7C84df9e7fe9f640afb435aaaaaaaaaaaa%7C1%7C0%7C639154590688098516%7CUnknown%7CTWFpbGZsb3d8eyJFbXB0eU1hcGkiOnRydWUsIlYiOiIwLjAuMDAwMCIsIlAiOiJXaW4zMiIsIkFOIjoiTWFpbCIsIldUIjoyfQ%3D%3D%7C0%7C%7C%7C&sdata=VF%2BMX8ji1HhChducfhL5iFC84692s9kVR%2BP10QBcZuY%3D&reserved=0)**). If this adherence statement is not accurate and there are restrictions on sharing of data and/or materials, please state these. Please note that we cannot proceed with consideration of your article until this information has been declared.**

**Please include both an updated Funding Statement and Competing Interests Statement in your cover letter. We will change the online submission form on your behalf.**

We thank the Editor for this important comment. We have the Competing Interests Statement to declare the commercial affiliation and to include the required statement regarding our adherence to PLOS ONE policies on sharing data and materials.

As requested, both revised statements have also been included in the cover letter.

*“Dear Editor,*

*We sincerely thank the Editors and Reviewers for their careful evaluation of our manuscript and for their constructive comments. We have carefully revised the manuscript accordingly and addressed all comments raised during the review process.*

*We are pleased to submit the revised version of our manuscript entitled “Monitoring Childbirth and Immediate Postpartum Care Quality: A Scoping Review of Indicators in High-Income Countries” for continued consideration in PLOS ONE.*

*In addition, we have addressed the editorial requests regarding the Funding Statement and Competing Interests Statement, including the commercial affiliation of RM with Digital Workplace Squared, IT Skills and Solutions, Geneva, Switzerland. The updated declarations are provided below.*

*Funding Statement*

*TD received funding from the Fonds de Recherche Impulsion HES-SO (Grant number 127974) and the Swiss National Science Foundation (grant number PT0043_222882). The funders provided support in the form of salary support for author TD but did not have any additional role in the study design, data collection and analysis, decision to publish, or preparation of the manuscript.*

*RM is employed by Digital Workplace Squared, IT Skills and Solutions, Geneva, Switzerland. The company provided support in the form of salary for author RM but had no additional role in the study design, data collection and analysis, decision to publish, or preparation of the manuscript. The specific roles of these authors are articulated in the "Author Contributions" section.*

*Competing Interests Statement*

*RM is the owner and operator of Digital Workplace Squared, IT Skills and Solutions, Geneva, Switzerland. This commercial affiliation did not influence the conduct, analysis, or reporting of this study. The authors declare that there are no other competing interests related to employment, consultancy, patents, products in development, or marketed products. This does not alter our adherence to PLOS ONE policies on sharing data and materials.*

*We hope that these revisions satisfactorily address the editorial requirements, and we thank you for your continued consideration of our manuscript.*

*Sincerely,*

*Thomas Desplanches”*

**6. Please include a caption for figure 1.**

We have added the caption for Figure 1 as requested (please see page 8 lines 185-187): **Fig 1. PRISMA 2020 flow diagram of the study selection process.** *Flow diagram illustrating the identification, screening, eligibility assessment, and inclusion of studies included in this scoping review.*

**7. Please include captions for your Supporting Information files at the end of your manuscript, and update any in-text citations to match accordingly. Please see our Supporting Information guidelines for more information:** [**http://journals.plos.org/plosone/s/supporting-information**](https://ita01.safelinks.protection.outlook.com/?url=http%3A%2F%2Fjournals.plos.org%2Fplosone%2Fs%2Fsupporting-information&data=05%7C02%7C%7Cb74c07a7044e4bad4f0708debbb6bce6%7C84df9e7fe9f640afb435aaaaaaaaaaaa%7C1%7C0%7C639154590688118638%7CUnknown%7CTWFpbGZsb3d8eyJFbXB0eU1hcGkiOnRydWUsIlYiOiIwLjAuMDAwMCIsIlAiOiJXaW4zMiIsIkFOIjoiTWFpbCIsIldUIjoyfQ%3D%3D%7C0%7C%7C%7C&sdata=jOcdpL4%2B8ZnWcmxyDxApsQc4UmHSbz45YtT%2FLL3FUsI%3D&reserved=0)**.**

**Captions**

**The supporting information name and number are required in a caption, and we highly recommend including a one-line title as well. You may also include a legend in your caption, but it is not required. Format your supporting information captions as follows:**

**S1 Text. Title is strongly recommended. Legend is optional.**

**In the published article, supporting information files are accessed only through a hyperlink attached to the captions. For this reason, you must list captions at the end of your manuscript file. You may include a caption within the supporting information file itself, as long as that caption is also provided in the manuscript file. Do not submit a separate caption file.**

We have added captions for all Supporting Information files at the end of the manuscript according to PLOS ONE formatting requirements (please page 80). We have also renamed the Supporting Information files and updated all in-text citations to ensure consistency with the required format.

Supporting information

S1 File: Preferred Reporting Items for Systematic reviews and Meta-Analyses extension for Scoping Reviews (PRISMA-ScR) Checklist.

S2 File. Research strategy.

S3 File. Final included articles Perinatal Care Quality Indicators.

S1 Table. Characteristics of included studies.

S2 Table. Socio-demographic indicators.

S3 Table. List of incomplete indicators.

**8. If the reviewer comments include a recommendation to cite specific previously published works, please review and evaluate these publications to determine whether they are relevant and should be cited. There is no requirement to cite these works unless the editor has indicated otherwise.**

This point is not applicable, as no reviewer recommendations to cite specific previously published works were provided.

*************************************

**Reviewer(s)' Comments to Author:**

**Reviewer: 1**

**Comments to the Author**

**Interesting study with presentation of a complex topic that requires thoughtful engagement with standardization of criteria and outcomes. Agree that it is difficult to compare outcomes across various healthcare systems especially with mixing of provided medical care versus private insurance.**

Thank you for your review, and your kind comment.

**Reviewer: 2**

**Comments to the Author**

**As the statistical reviewer I will focus on methods and reporting. There is not much to comment on in terms of statistics but I do have some suggestions, mainly to properly acknowledge the limitations.**

**Major**

**1) The paper largely follows the relevant checklist, but not fully. However, no protocol registration is reported, which limits transparency, and no formal risk‑of‑bias or quality assessment is conducted. The authors do use an adapted validity score, but that is not equivalent to standard critical appraisal. In addition, some reporting details (such as reasons for exclusion and study-level characteristics) are not as complete as they could be. Discuss the first two as limitations (to be fair the quality assessment already is) and be more clear in the text about exclusions.**

We thank the reviewer for these important comments which have helped us improve the manuscript.

We conducted and reported this scoping review in accordance with the PRISMA-ScR checklist, which recommends reporting whether a review protocol exists and, where applicable, providing registration details. As no protocol was prospectively registered or published for this scoping review, we have acknowledged this as a limitation in the revised manuscript. However, all methodological steps, including the eligibility criteria, search strategy, study selection process, data extraction, and analytical approach, are reported in sufficient detail to support the transparency and reproducibility of the review.

To address this comment, we have revised the Strengths and limitations section to include the following statement (please see pages 64, lines 314–318):

*"Although no protocol was prospectively registered or published for this scoping review, all methodological steps, including the eligibility criteria, search strategy, study selection process, data extraction, and analytical approach, are reported in sufficient detail to support the transparency and reproducibility of the review."*

To address the reviewer's comment regarding the absence of a formal critical appraisal and risk-of-bias assessment, we have revised the limitations section. As outlined in the updated methodological guidance for scoping reviews (Pollock, et al. 2024. how-to: scoping review? Available from: <https://doi.org/10.1016/j.jclinepi.2024.111572>), formal critical appraisal is not a mandatory component of a scoping review and should be undertaken only when it is relevant to the objectives of the review.

We have revised the Strengths and limitations section (please see page 64, lines 322-325):

*“No formal critical appraisal of the included studies was undertaken. Although this is consistent with current methodological guidance for scoping reviews when such an appraisal is not required to address the review objectives (114), it limits the ability to comment on the methodological quality of the included evidence”.*

We have also clarified the rationale for using an adapted validity scale, which addressed a different objective of the review (please see page 64, lines 325-329):

*"In addition, as this review also assessed the level of validity of the identified quality indicators, we applied an adapted validity scale based on prior work. This assessment focused on the reported methods used for the development and validation of the identified quality indicators and was therefore dependent on the completeness of reporting in the included publications."*

In addition, we updated the PRISMA-ScR checklist (S1 File) by marking Item 12 ("Critical appraisal of individual sources of evidence") as "Not applicable", reflecting that no formal critical appraisal was undertaken, consistent with current methodological guidance for scoping reviews (Pollock et al., 2024).

We have also revised the Methods section to clarify how the adapted validity scale was applied (please see page 9, lines 157–165):

*“To provide a structured assessment of the reported level of validity of the identified quality indicators, we adapted the validity scale from a previous systematic review (8)”.*

Finally, to improve the clarity of the study selection process, we expanded the description of the eligibility criteria in the Methods section by providing a more explicit explanation of the reasons for study exclusion (please see pages 6-7, lines 97-100):

*"Studies were excluded if they focused on populations, outcomes, or time periods outside the scope of this review, or if the indicator related exclusively to phases of care other than childbirth and the immediate postpartum period. Publications were also excluded if the study design was not suitable for data extraction (e.g. commentaries, protocols, reviews, conference abstracts, and case reports or case series)."*

We also revised the Search strategy and screening processes section to clarify that studies were excluded according to these predefined eligibility criteria and that the reasons for exclusion at the full-text screening stage are summarized in the PRISMA flow diagram (Fig 1) (please see page 8, lines 125-127):

*"Studies were excluded according to the predefined eligibility criteria described above. Reasons for exclusion at the full-text screening stage are reported in the PRISMA flow diagram (Fig 1)”.*

In addition, to improve the reporting of study-level characteristics, we expanded S1 Table to include the study design and data source for each included study.

**2) Please describe the manual search of indicator databases in enough detail to ensure reproducibility.**

We thank the reviewer for this helpful comment. To improve the transparency and reproducibility of the search strategy, we have expanded the description of the manual search in the Methods section (please see page 8, lines 132-137).

*“Manual searches of national and international indicator databases from high-income countries were initially conducted in April 2024 and updated in March 2025,* *based on the World Bank classification (25). Searches were performed using the Google search engine with the search term “maternal and neonatal quality-of-care health indicators”, with a particular focus on national organizations responsible for developing or reporting maternal and neonatal quality-of-care indicators.”*

**3) Another limitation to discuss is the fact around 40% of indicators were incomplete. This may introduce bias in the work.**

We thank the reviewer for this important comment. We agree that the high proportion of incompletely reported indicators deserves further discussion. We consider this finding to be an important result of the review, reflecting limitations in the reporting and development of published quality indicators, rather than a methodological limitation of the review itself.

We have therefore expanded the Discussion to better interpret this finding and its implications for future indicator development and validation (please see page 63, lines 293-298).

*“Indicator quality itself remains a major concern. A substantial proportion (42%) of the indicators identified in this review were incompletely reported, lacking the basic elements required for meaningful measurement, including clear definitions and specified numerators or denominators. As a result, these indicators cannot be consistently interpreted or implemented across settings, highlighting important limitations in the current quality indicator literature”.*

**4) Although studies are drawn from multiple high-income countries, regional differences in indicator development and use are not meaningfully explored.**

We thank the reviewer for this valuable comment. We agree that regional differences in the development and use of quality indicators are an important consideration. However, the primary objective of this scoping review was to identify quality indicators used in high-income countries and assess their level of validity, rather than to compare regional differences. Such comparisons are influenced by differences in healthcare systems, policy contexts, and data infrastructures and were therefore beyond the scope of this review. We agree that this is an important area for future research.

Accordingly, we have expanded the Discussion to highlight the need for future studies exploring regional variation in the development, implementation, and use of quality indicators across healthcare systems (please see page 62, lines 273–276).

*“Regional differences in healthcare systems, policy priorities, and data infrastructures may also influence the development, selection, and implementation of quality indicators. Future research should explore these contextual differences to better understand their impact on the comparability and applicability of quality indicators across high-income countries”.*

**Minor
1) it is unusual to introduce validity scoring in a scoping review, and this makes the work move towards evaluative synthesis. Perhaps the authors should expand on their reasoning for this.**

We thank the reviewer for this helpful comment. We agree that the use of a validity score is uncommon in scoping reviews. In our review, the adapted validity scale was used to provide a structured assessment of the validity of the identified quality indicators rather than to perform a formal critical appraisal of the included studies.

We have clarified this rationale in both the Methods and Strengths and limitations sections of the revised manuscript.

Methods section please see page 9, lines 157–165: *“To provide a structured assessment of the reported level of validity of the identified quality indicators, we adapted the validity scale from a previous systematic review (8)”.*

Limitation section: please see page 64, lines 325-329: *"In addition, as this review also assessed the level of validity of the identified quality indicators, we applied an adapted validity scale based on prior work. This assessment focused on the reported methods used for the development and validation of the identified quality indicators and was therefore dependent on the completeness of reporting in the included publications."*
