# Supplementary material for: Monitoring childbirth and immediate postpartum care quality: A scoping review of indicators in high-income countries
Source: PLoS One. 2026 Sep 15;21(9):e0350665. doi: 10.1371/journal.pone.0350665 (PMC13577461; doi:10.1371/journal.pone.0350665)
Supplement: Supplementary file 14 [file pone.0350665.s003.html]

Perinatal Care Quality Indicators


# Perinatal Care Quality Indicators

Extracted from scoping review written by Allison Warren, Dayana Hamparsoumian, Elsa Lorthe, Gilles Cattani, [list of co-author], Laurent Gaucher, Thomas Desplanches
