# Supplementary material for: Monitoring childbirth and immediate postpartum care quality: A scoping review of indicators in high-income countries
Source: PLoS One. 2026 Sep 15;21(9):e0350665. doi: 10.1371/journal.pone.0350665 (PMC13577461; doi:10.1371/journal.pone.0350665)
Supplement: Peer review history

# Peer review: Decision Letter 0

**DOI:** 10.1371/journal.pone.0350665.r001

**Authors:** Umberto Simeoni

27 May 2026

Dear Dr. Desplanches,

Please submit your revised manuscript by Jul 11 2026 11:59PM. If you will need more time than this to complete your revisions, please reply to this message or contact the journal office at plosone@plos.org. When you're ready to submit your revision, log on to https://www.editorialmanager.com/pone/ and select the 'Submissions Needing Revision' folder to locate your manuscript file.

- A letter that responds to each point raised by the academic editor and reviewer(s). You should upload this letter as a separate file labeled 'Response to Reviewers'.
- A marked-up copy of your manuscript that highlights changes made to the original version. You should upload this as a separate file labeled 'Revised Manuscript with Track Changes'.
- An unmarked version of your revised paper without tracked changes. You should upload this as a separate file labeled 'Manuscript'.

If you would like to make changes to your financial disclosure, please include your updated statement in your cover letter. Guidelines for resubmitting your figure files are available below the reviewer comments at the end of this letter.

If applicable, we recommend that you deposit your laboratory protocols in protocols.io to enhance the reproducibility of your results. Protocols.io assigns your protocol its own identifier (DOI) so that it can be cited independently in the future. For instructions see: https://journals.plos.org/plosone/s/submission-guidelines#loc-laboratory-protocols. Additionally, PLOS ONE offers an option for publishing peer-reviewed Lab Protocol articles, which describe protocols hosted on protocols.io. Read more information on sharing protocols at https://plos.org/protocols?utm_medium=editorial-email&utm_source=authorletters&utm_campaign=protocols.

As the corresponding author, your ORCID iD is verified in the submission system and will appear in the published article. PLOS supports the use of ORCID, and we encourage all coauthors to register for an ORCID iD and use it as well. Please encourage your coauthors to verify their ORCID iD within the submission system before final acceptance, as unverified ORCID iDs will not appear in the published article. *Only*  the individual author can complete the verification step; PLOS staff *cannot*  verify ORCID iDs on behalf of authors.

We look forward to receiving your revised manuscript.

Kind regards,

Umberto Simeoni

Academic Editor

PLOS One

Journal Requirements:

When submitting your revision, we need you to address these additional requirements.

1.Please ensure that your manuscript meets PLOS ONE's style requirements, including those for file naming. The PLOS ONE style templates can be found at

https://journals.plos.org/plosone/s/file?id=wjVg/PLOSOne_formatting_sample_main_body.pdf and

https://journals.plos.org/plosone/s/file?id=ba62/PLOSOne_formatting_sample_title_authors_affiliations.pdf

2. Please note that funding information should not appear in any section or other areas of your manuscript. We will only publish funding information present in the Funding Statement section of the online submission form. Please remove any funding-related text from the manuscript.

3. We note that the grant information you provided in the ‘Funding Information’ and ‘Financial Disclosure’ sections do not match.

When you resubmit, please ensure that you provide the correct grant numbers for the awards you received for your study in the ‘Funding Information’ section.

4. We note that your Data Availability Statement is currently as follows: All relevant data are within the manuscript and in Supporting Information files.

Please confirm at this time whether or not your submission contains all raw data required to replicate the results of your study. Authors must share the “minimal data set” for their submission. PLOS defines the minimal data set to consist of the data required to replicate all study findings reported in the article, as well as related metadata and methods (https://journals.plos.org/plosone/s/data-availability#loc-minimal-data-set-definition).

For example, authors should submit the following data:

- The values behind the means, standard deviations and other measures reported;

- The values used to build graphs;

- The points extracted from images for analysis.

Authors do not need to submit their entire data set if only a portion of the data was used in the reported study.

If your submission does not contain these data, please either upload them as Supporting Information files or deposit them to a stable, public repository and provide us with the relevant URLs, DOIs, or accession numbers. For a list of recommended repositories, please see https://journals.plos.org/plosone/s/recommended-repositories.

If there are ethical or legal restrictions on sharing a de-identified data set, please explain them in detail (e.g., data contain potentially sensitive information, data are owned by a third-party organization, etc.) and who has imposed them (e.g., an ethics committee). Please also provide contact information for a data access committee, ethics committee, or other institutional body to which data requests may be sent. If data are owned by a third party, please indicate how others may request data access.

5. Thank you for stating the following in the Competing Interests section:

“NO authors have competing interests”

We note that one or more of the authors are employed by a commercial company: Digital Workplace Squared, IT Skills and Solutions, Geneva, Switzerland

1.        Please provide an amended Funding Statement declaring this commercial affiliation, as well as a statement regarding the Role of Funders in your study. If the funding organization did not play a role in the study design, data collection and analysis, decision to publish, or preparation of the manuscript and only provided financial support in the form of authors' salaries and/or research materials, please review your statements relating to the author contributions, and ensure you have specifically and accurately indicated the role(s) that these authors had in your study. You can update author roles in the Author Contributions section of the online submission form.

Please also include the following statement within your amended Funding Statement.

“The funder provided support in the form of salaries for authors [insert relevant initials], but did not have any additional role in the study design, data collection and analysis, decision to publish, or preparation of the manuscript. The specific roles of these authors are articulated in the ‘author contributions’ section.”

If your commercial affiliation did play a role in your study, please state and explain this role within your updated Funding Statement.

2. Please also provide an updated Competing Interests Statement declaring this commercial affiliation along with any other relevant declarations relating to employment, consultancy, patents, products in development, or marketed products, etc.

Within your Competing Interests Statement, please confirm that this commercial affiliation does not alter your adherence to all PLOS ONE policies on sharing data and materials by including the following statement: "This does not alter our adherence to  PLOS ONE policies on sharing data and materials.” (as detailed online in our guide for authors http://journals.plos.org/plosone/s/competing-interests) . If this adherence statement is not accurate and  there are restrictions on sharing of data and/or materials, please state these. Please note that we cannot proceed with consideration of your article until this information has been declared.

Please include both an updated Funding Statement and Competing Interests Statement in your cover letter. We will change the online submission form on your behalf.

6. Please include a caption for figure 1.

7. Please include captions for your Supporting Information files at the end of your manuscript, and update any in-text citations to match accordingly. Please see our Supporting Information guidelines for more information: http://journals.plos.org/plosone/s/supporting-information.

8. If the reviewer comments include a recommendation to cite specific previously published works, please review and evaluate these publications to determine whether they are relevant and should be cited. There is no requirement to cite these works unless the editor has indicated otherwise.

Additional Editor Comments:

Please take into consideration all the remarks made by Reviewer n°2 about the study methods and statistical analysis.

[Note: HTML markup is below. Please do not edit.]

Reviewers' comments:

Reviewer's Responses to Questions

**Comments to the Author**

1. Is the manuscript technically sound, and do the data support the conclusions?

Reviewer #1: Yes

Reviewer #2: Yes

**********

2. Has the statistical analysis been performed appropriately and rigorously?

Reviewer #1: Yes

Reviewer #2: Yes

**********

3. Have the authors made all data underlying the findings in their manuscript fully available?

The PLOS Data policy

Reviewer #1: Yes

Reviewer #2: Yes

**********

4. Is the manuscript presented in an intelligible fashion and written in standard English?

Reviewer #1: Yes

Reviewer #2: Yes

**********

Reviewer #1: Interesting study with presentation of a complex topic that requires thoughtful engagement with standardization of criteria and outcomes. Agree that it is difficult to compare outcomes across various healthcare systems especially with mixing of provided medical care versus private insurance.

Reviewer #2: As the statistical reviewer I will focus on methods and reporting. There is not much to comment on in terms of statistics but I do have some suggestions, mainly to properly acknowledge the limitations.

Major

1) The paper largely follows the relevant checklist, but not fully. However, no protocol registration is reported, which limits transparency, and no formal risk‑of‑bias or quality assessment is conducted. The authors do use an adapted validity score, but that is not equivalent to standard critical appraisal. In addition, some reporting details (such as reasons for exclusion and study-level characteristics) are not as complete as they could be. Discuss the first two as limitations (to be fair the quality assessment already is) and be more clear in the text about exclusions.

2) Please describe the manual search of indicator databases in enough detail to ensure reproducibility.

3) another limitation to discuss is the fact around 40% of indicators were incomplete. this may introduce bias in the work.

4) Although studies are drawn from multiple high-income countries, regional differences in indicator development and use are not meaningfully explored.

Minor

1) it is unusual to introduce validity scoring in a scoping review, and this makes the work move towards evaluative synthesis. Perhaps the authors should expand on their reasonging for this.

**********

what does this mean?). If published, this will include your full peer review and any attached files.

If you choose “no”, your identity will remain anonymous but your review may still be made public.

**Do you want your identity to be public for this peer review?**  For information about this choice, including consent withdrawal, please see our Privacy Policy

Reviewer #1: No

Reviewer #2: No

**********

[NOTE: If reviewer comments were submitted as an attachment file, they will be attached to this email and accessible via the submission site. Please log into your account, locate the manuscript record, and check for the action link "View Attachments". If this link does not appear, there are no attachment files.]

To ensure your figures meet our technical requirements, please review our figure guidelines: https://journals.plos.org/plosone/s/figures

You may also use PLOS’s free figure tool, NAAS, to help you prepare publication quality figures: https://journals.plos.org/plosone/s/figures#loc-tools-for-figure-preparation.

NAAS will assess whether your figures meet our technical requirements by comparing each figure against our figure specifications.

# Author comment: Author response to Decision Letter 1

**DOI:** 10.1371/journal.pone.0350665.r002

9 Jul 2026

Dear Editor,

We sincerely thank the Editors and Reviewers for their careful evaluation of our manuscript. We have revised the article accordingly and addressed all the comments raised.

Editor comments to Author:

When submitting your revision, we need you to address these additional requirements.

1.Please ensure that your manuscript meets PLOS ONE's style requirements, including those for file naming. The PLOS ONE style templates can be found at https://journals.plos.org/plosone/s/file?id=wjVg/PLOSOne_formatting_sample_main_body.pdf and https://journals.plos.org/plosone/s/file?id=ba62/PLOSOne_formatting_sample_title_authors_affiliations.pdf

We thank the Editor for this comment. We have carefully checked the manuscript against the PLOS ONE style requirements, including the formatting instructions and file naming guidelines.

All necessary modifications have been implemented to ensure full compliance with the journal’s requirements.

2. Please note that funding information should not appear in any section or other areas of your manuscript. We will only publish funding information present in the Funding Statement section of the online submission form. Please remove any funding-related text from the manuscript.

The funding section was removed as requested from the manuscript. Funding details are now provided exclusively in the Funding Statement section of the online submission form.

3. We note that the grant information you provided in the ‘Funding Information’ and ‘Financial Disclosure’ sections do not match. When you resubmit, please ensure that you provide the correct grant numbers for the awards you received for your study in the ‘Funding Information’ section.

Thank you for this comment. We have updated both the ‘Funding Information’ and ‘Financial Disclosure’ sections as requested to match and included the grant information.

4. We note that your Data Availability Statement is currently as follows: All relevant data are within the manuscript and in Supporting Information files.

Please confirm at this time whether or not your submission contains all raw data required to replicate the results of your study. Authors must share the “minimal data set” for their submission. PLOS defines the minimal data set to consist of the data required to replicate all study findings reported in the article, as well as related metadata and methods (https://journals.plos.org/plosone/s/data-availability#loc-minimal-data-set-definition).

For example, authors should submit the following data:

- The values behind the means, standard deviations and other measures reported;

- The values used to build graphs;

- The points extracted from images for analysis.

Authors do not need to submit their entire data set if only a portion of the data was used in the reported study.

If your submission does not contain these data, please either upload them as Supporting Information files or deposit them to a stable, public repository and provide us with the relevant URLs, DOIs, or accession numbers. For a list of recommended repositories, please see https://journals.plos.org/plosone/s/recommended-repositories.

If there are ethical or legal restrictions on sharing a de-identified data set, please explain them in detail (e.g., data contain potentially sensitive information, data are owned by a third-party organization, etc.) and who has imposed them (e.g., an ethics committee). Please also provide contact information for a data access committee, ethics committee, or other institutional body to which data requests may be sent. If data are owned by a third party, please indicate how others may request data access.

We thank the Editor for this comment.

In accordance with PLOS ONE’s data availability requirements, we confirm that all materials necessary to reproduce our scoping review findings are provided within the manuscript and Supporting Information files. Specifically, we have amended the included studies list (S1 Table. Characteristics of included studies) to include the study design and reported data sources.

As this study is a scoping review, the minimal dataset consists of the complete list of included studies and the extracted study-level information used to identify, map, and classify childbirth and immediate postpartum care quality indicators.

The supplementary materials include the complete search strategy, the full list of included studies, and all extracted information required to reproduce the results, tables, and figures reported in the manuscript.

Consequently, we have amended the Data Availability Statement to better specify the minimal dataset supporting this scoping review. The revised statement now reads:

“All data required to replicate the findings of this scoping review are available within the manuscript and Supporting Information files. The minimal dataset consists of the complete list of included studies and the extracted study-level information used to identify, map, and classify childbirth and immediate postpartum care quality indicators (S1 Table. Characteristics of included studies).”

5. Thank you for stating the following in the Competing Interests section:

“NO authors have competing interests”

We note that one or more of the authors are employed by a commercial company: Digital Workplace Squared, IT Skills and Solutions, Geneva, Switzerland

1. Please provide an amended Funding Statement declaring this commercial affiliation, as well as a statement regarding the Role of Funders in your study. If the funding organization did not play a role in the study design, data collection and analysis, decision to publish, or preparation of the manuscript and only provided financial support in the form of authors' salaries and/or research materials, please review your statements relating to the author contributions, and ensure you have specifically and accurately indicated the role(s) that these authors had in your study. You can update author roles in the Author Contributions section of the online submission form.

Please also include the following statement within your amended Funding Statement.

“The funder provided support in the form of salaries for authors [insert relevant initials], but did not have any additional role in the study design, data collection and analysis, decision to publish, or preparation of the manuscript. The specific roles of these authors are articulated in the ‘author contributions’ section.”

If your commercial affiliation did play a role in your study, please state and explain this role within your updated Funding Statement.

We thank the Editor for this comment. We have amended the Funding Statement to clarify the funding sources, the role of the funders, and the commercial affiliation of RM with Digital Workplace Squared, IT Skills and Solutions, Geneva, Switzerland.

The Funding Statement has been updated as follows:

“TD received funding from the Fonds de Recherche Impulsion HES-SO (Grant number 127974) and the Swiss National Science Foundation (grant number PT0043_222882). The funders provided support in the form of salary support for author TD but did not have any additional role in the study design, data collection and analysis, decision to publish, or preparation of the manuscript.

“RM is employed by Digital Workplace Squared, IT Skills and Solutions, Geneva, Switzerland. The company provided support in the form of salary for author RM but had no additional role in the study design, data collection and analysis, decision to publish, or preparation of the manuscript. RM contributed to this work independently of her commercial affiliation. The specific roles of these authors are articulated in the ‘Author Contributions’ section”.

2. Please also provide an updated Competing Interests Statement declaring this commercial affiliation along with any other relevant declarations relating to employment, consultancy, patents, products in development, or marketed products, etc.

Within your Competing Interests Statement, please confirm that this commercial affiliation does not alter your adherence to all PLOS ONE policies on sharing data and materials by including the following statement: "This does not alter our adherence to PLOS ONE policies on sharing data and materials.” (as detailed online in our guide for authors http://journals.plos.org/plosone/s/competing-interests). If this adherence statement is not accurate and there are restrictions on sharing of data and/or materials, please state these. Please note that we cannot proceed with consideration of your article until this information has been declared.

Please include both an updated Funding Statement and Competing Interests Statement in your cover letter. We will change the online submission form on your behalf.

We thank the Editor for this important comment. We have the Competing Interests Statement to declare the commercial affiliation and to include the required statement regarding our adherence to PLOS ONE policies on sharing data and materials.

As requested, both revised statements have also been included in the cover letter.

“Dear Editor,

We sincerely thank the Editors and Reviewers for their careful evaluation of our manuscript and for their constructive comments. We have carefully revised the manuscript accordingly and addressed all comments raised during the review process.

We are pleased to submit the revised version of our manuscript entitled “Monitoring Childbirth and Immediate Postpartum Care Quality: A Scoping Review of Indicators in High-Income Countries” for continued consideration in PLOS ONE.

In addition, we have addressed the editorial requests regarding the Funding Statement and Competing Interests Statement, including the commercial affiliation of RM with Digital Workplace Squared, IT Skills and Solutions, Geneva, Switzerland. The updated declarations are provided below.

Funding Statement

TD received funding from the Fonds de Recherche Impulsion HES-SO (Grant number 127974) and the Swiss National Science Foundation (grant number PT0043_222882). The funders provided support in the form of salary support for author TD but did not have any additional role in the study design, data collection and analysis, decision to publish, or preparation of the manuscript.

RM is employed by Digital Workplace Squared, IT Skills and Solutions, Geneva, Switzerland. The company provided support in the form of salary for author RM but had no additional role in the study design, data collection and analysis, decision to publish, or preparation of the manuscript. The specific roles of these authors are articulated in the "Author Contributions" section.

Competing Interests Statement

RM is the owner and operator of Digital Workplace Squared, IT Skills and Solutions, Geneva, Switzerland. This commercial affiliation did not influence the conduct, analysis, or reporting of this study. The authors declare that there are no other competing interests related to employment, consultancy, patents, products in development, or marketed products. This does not alter our adherence to PLOS ONE policies on sharing data and materials.

We hope that these revisions satisfactorily address the editorial requirements, and we thank you for your continued consideration of our manuscript.

Sincerely,

Thomas Desplanches”

6. Please include a caption for figure 1.

We have added the caption for Figure 1 as requested (please see page 8 lines 185-187): Fig 1. PRISMA 2020 flow diagram of the study selection process. Flow diagram illustrating the identification, screening, eligibility assessment, and inclusion of studies included in this scoping review.

7. Please include captions for your Supporting Information files at the end of your manuscript, and update any in-text citations to match accordingly. Please see our Supporting Information guidelines for more information: http://journals.plos.org/plosone/s/supporting-information.

Captions

The supporting information name and number are required in a caption, and we highly recommend including a one-line title as well. You may also include a legend in your caption, but it is not required. Format your supporting information captions as follows:

S1 Text. Title is strongly recommended. Legend is optional.

In the published article, supporting information files are accessed only through a hyperlink attached to the captions. For this reason, you must list captions at the end of your manuscript file. You may include a caption within the supporting information file itself, as long as that caption is also provided in the manuscript file. Do not submit a separate caption file.

We have added captions for all Supporting Information files at the end of the manuscript according to PLOS ONE formatting requirements (please page 80). We have also renamed the Supporting Information files and updated all in-text citations to ensure consistency with the required format.

Supporting information

S1 File: Preferred Reporting Items for Systematic reviews and Meta-Analyses extension for Scoping Reviews (PRISMA-ScR) Checklist.

S2 File. Research strategy.

S3 File. Final included articles Perinatal Care Quality Indicators.

S1 Table. Characteristics of included studies.

S2 Table. Socio-demographic indicators.

S3 Table. List of incomplete indicators.

8. If the reviewer comments include a recommendation to cite specific previously published works, please review and evaluate these publications to determine whether they are relevant and should be cited. There is no requirement to cite these works unless the editor has indicated otherwise.

This point is not applicable, as no reviewer recommendations to cite specific previously published works were provided.

*************************************

Reviewer(s)' Comments to Author:

Reviewer: 1

Comments to the Author

Interesting study with presentation of a complex topic that requires thoughtful engagement with standardization of criteria and outcomes. Agree that it is difficult to compare outcomes across various healthcare systems especially with mixing of provided medical care versus private insurance.

Thank you for your review, and your kind comment.

*************************************

Reviewer: 2

Comments to the Author

As the statistical reviewer I will focus on methods and reporting. There is not much to comment on in terms of statistics but I do have some suggestions, mainly to properly acknowledge the limitations.

Major

1) The paper largely follows the relevant checklist, but not fully. However, no protocol registration is reported, which limits transparency, and no formal risk of bias or quality assessment is conducted. The authors do use an adapted validity score, but that is not equivalent to standard critical appraisal. In addition, some reporting details (such as reasons for exclusion and study-level characteristics) are not as complete as they could be. Discuss the first two as limitations (to be fair the quality assessment already is) and be more clear in the text about exclusions.

We thank the reviewer for these important comments which have helped us improve the manuscript.

We conducted and reported this scoping review in accordance with the PRISMA-ScR checklist, which recommends reporting whether a review protocol exists and, where applicable, providing registration details. As no protocol was prospectively registered or published for this scoping review, we have acknowledged this as a limitation in the revised manuscript. However, all methodological steps, including the eligibility criteria, search strategy, study selection process, data extraction, and analytical approach, are reported in sufficient detail to support the transparency and reproducibility of the review.

To address this comment, we have revised the Strengths and limitations section to include the following statement (please see pages 64, lines 314–318):

"Although no protocol was prospectively registered or published for this scoping review, all methodological steps, including the eligibility criteria, search strategy, study selection process, data extraction, and analytical approach, are reported in sufficient detail to support the transparency and reproducibility of the review."

To address the reviewer's comment regarding the absence of a formal critical appraisal and risk-of-bias asse

Attachment Submitted filename: Response_Reviewers_R1.docx

# Peer review: Decision Letter 1

**DOI:** 10.1371/journal.pone.0350665.r003

**Authors:** Umberto Simeoni

23 Aug 2026

Monitoring childbirth and immediate postpartum care quality: A scoping review of indicators in high-income countries

PONE-D-26-12819R1

Dear Dr. Desplanches,

We’re pleased to inform you that your manuscript has been judged scientifically suitable for publication and will be formally accepted for publication once it meets all outstanding technical requirements.

Within one week, you’ll receive an e-mail detailing the required amendments. When these have been addressed, you’ll receive a formal acceptance letter and your manuscript will be scheduled for publication.

An invoice will be generated when your article is formally accepted. Please note, if your institution has a publishing partnership with PLOS and your article meets the relevant criteria, all or part of your publication costs will be covered. Please make sure your user information is up-to-date by logging into Editorial Manager at Editorial Manager® and clicking the ‘Update My Information' link at the top of the page. For questions related to billing, please contact billing support.

If your institution or institutions have a press office, please notify them about your upcoming paper to help maximize its impact. If they’ll be preparing press materials, please inform our press team as soon as possible -- no later than 48 hours after receiving the formal acceptance. Your manuscript will remain under strict press embargo until 2 pm Eastern Time on the date of publication. For more information, please contact onepress@plos.org.

Kind regards,

Umberto Simeoni

Academic Editor

PLOS One

Additional Editor Comments (optional):

Reviewers' comments:

Reviewer's Responses to Questions

**Comments to the Author**

Reviewer #2: All comments have been addressed

**********

2. Is the manuscript technically sound, and do the data support the conclusions?

Reviewer #2: Yes

**********

3. Has the statistical analysis been performed appropriately and rigorously?

Reviewer #2: Yes

**********

4. Have the authors made all data underlying the findings in their manuscript fully available?

The PLOS Data policy

Reviewer #2: Yes

**********

5. Is the manuscript presented in an intelligible fashion and written in standard English?

Reviewer #2: (No Response)

**********

Reviewer #2: I am satisfied with the authors' responses and the resulting changes to the paper. nothing else to add.

**********

what does this mean?). If published, this will include your full peer review and any attached files.

If you choose “no”, your identity will remain anonymous but your review may still be made public.

**Do you want your identity to be public for this peer review?**  For information about this choice, including consent withdrawal, please see our Privacy Policy

Reviewer #2: No

**********

# Editor report: Acceptance letter

**DOI:** 10.1371/journal.pone.0350665.r004

**Authors:** Umberto Simeoni

PONE-D-26-12819R1

PLOS One

Dear Dr. Desplanches,

I'm pleased to inform you that your manuscript has been deemed suitable for publication in PLOS One. Congratulations! Your manuscript is now being handed over to our production team.

At this stage, our production department will prepare your paper for publication. This includes ensuring the following:

* All references, tables, and figures are properly cited

* All relevant supporting information is included in the manuscript submission,

* There are no issues that prevent the paper from being properly typeset

You will receive further instructions from the production team, including instructions on how to review your proof when it is ready. Please keep in mind that we are working through a large volume of accepted articles, so please give us a few days to review your paper and let you know the next and final steps.

Lastly, if your institution or institutions have a press office, please let them know about your upcoming paper now to help maximize its impact. If they'll be preparing press materials, please inform our press team within the next 48 hours. Your manuscript will remain under strict press embargo until 2 pm Eastern Time on the date of publication. For more information, please contact onepress@plos.org.

You will receive an invoice from PLOS for your publication fee after your manuscript has reached the completed accept phase. If you receive an email requesting payment before acceptance or for any other service, this may be a phishing scheme. Learn how to identify phishing emails and protect your accounts at https://explore.plos.org/phishing.

If we can help with anything else, please email us at customercare@plos.org.

Thank you for submitting your work to PLOS One and supporting open access.

Kind regards,

PLOS One Editorial Office Staff

on behalf of

Prof. Umberto Simeoni

Academic Editor

PLOS One
